# Improving the Accuracy of Progress Indication for Constructing Deep Learning Models

**DOI:** 10.1109/access.2022.3181493

**Published:** 2022-06-08

**Authors:** QIFEI DONG, XIAOYI ZHANG, GANG LUO

**Affiliations:** Department of Biomedical Informatics and Medical Education, University of Washington, Seattle, WA 98195, USA

**Keywords:** Progress indicator, deep learning, TensorFlow, model construction

## Abstract

For many machine learning tasks, deep learning greatly outperforms all other existing learning algorithms. However, constructing a deep learning model on a big data set often takes days or months. During this long process, it is preferable to provide a progress indicator that keeps predicting the model construction time left and the percentage of model construction work done. Recently, we developed the first method to do this that permits early stopping. That method revises its predicted model construction cost using information gathered at the validation points, where the model’s error rate is computed on the validation set. Due to the sparsity of validation points, the resulting progress indicators often have a long delay in gathering information from enough validation points and obtaining relatively accurate progress estimates. In this paper, we propose a new progress indication method to overcome this shortcoming by judiciously inserting extra validation points between the original validation points. We implemented this new method in TensorFlow. Our experiments show that compared with using our prior method, using this new method reduces the progress indicator’s prediction error of the model construction time left by 57.5% on average. Also, with a low overhead, this new method enables us to obtain relatively accurate progress estimates faster.

## I. INTRODUCTION

### A. OUR PRIOR PROGRESS INDICATION METHOD FOR CONSTRUCTING DEEP LEARNING MODELS

**FIGURE 1.**
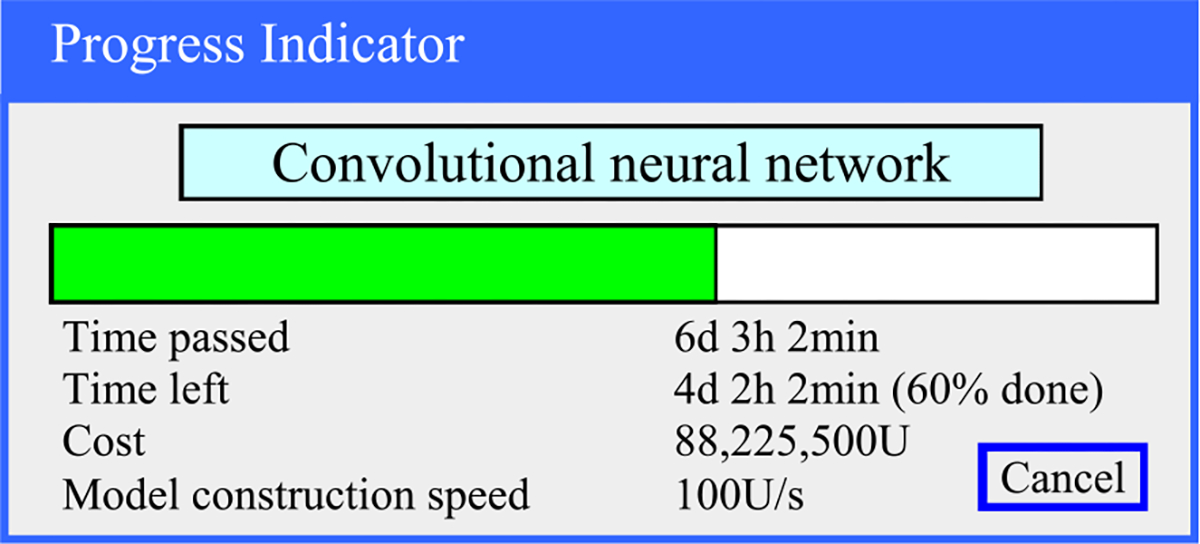
An example progress indicator for constructing deep learning models.

For many machine learning tasks such as image segmentation, machine translation, video classification, and speech recognition, deep learning greatly outperforms all other existing learning algorithms [1]. However, even with a cluster of graphics processing unit (GPU) or tensor processing unit (TPU) nodes, it often takes days or months to construct a deep learning model on a big data set [2]–[5]. During this long process, it is preferable to provide a progress indicator that keeps predicting the model construction time left and the percentage of model construction work done as shown in Fig. 1. This improves the user-friendliness of model construction. Also, the information supplied by the progress indicator can be used to aid workload management [6]–[8].

Recently, we developed the first method to build sophisticated progress indicators for constructing deep learning models that permits early stopping [8]. This method computes progress estimates for the model construction process using information gathered at the validation points, where the model’s error rate is computed on the validation set. Despite producing useful results, this method has a shortcoming. Due to the sparsity of validation points, the resulting progress indicators often have a long delay in obtaining relatively accurate progress estimates. More specifically, at the beginning of model construction, we come up with a crude estimate of the model construction cost that is usually inaccurate. At least three data points are needed to estimate the three parameters of the regression function that is used to predict the model construction cost. Consequently, the predicted model construction cost is revised starting from the third validation point, which is too late. Then a revision is made only at each subsequent validation point, which is infrequent. The combination of these two factors often causes a long delay in gathering information from enough validation points and obtaining relatively accurate progress estimates.

For example, Goyal *et al.* [9] used eight Nvidia Tesla P100 GPUs to train the ResNet-50 convolutional neural network on the ImageNet Large-Scale Visual Recognition Competition (ILSVRC) data set [10]. About 19 minutes passed between two successive validation points [9], [11]. By the time the progress indicator revised its predicted model construction cost for the first time, 3 × 19 = 57 minutes had elapsed. This is a long delay that takes up a non-trivial fraction of the 29-hour model construction time [9].

### B. OUR CONTRIBUTIONS

The objective of this research work is to overcome our prior progress indication method’s [8] shortcoming of having a long delay in obtaining relatively accurate progress estimates for the deep learning model construction process. To obtain relatively accurate progress estimates faster, in this paper we propose a new progress indication method for constructing deep learning models that judiciously inserts extra validation points between the original validation points. The predicted model construction cost is revised at both the original and the added validation points. Consequently, compared with our prior progress indication method [8], our new progress indication method starts revising the predicted model construction cost earlier and revises the predicted model construction cost more frequently. This helps the progress indicator reduce its prediction error of the model construction time left and obtain relatively accurate progress estimates faster.

A good progress indicator should have a low run-time overhead [6]. In our case, a large part of the progress indicator’s run-time overhead comes from computing the model’s error rate at the added validation points. To lower this part of the run-time overhead, at each added validation point, we calculate the model’s error rate on a randomly sampled subset of the full validation set rather than on the full validation set.

To fill in the rest of our new progress indication method, we need to solve three technical challenges. First, we need to set 1) *n_j_* (*j* ≥ 0), the count of validation points to be added between the *j*-th and the (*j* + 1)-th original validation points, and 2) *V*′, the uniform size of the randomly sampled subset of the full validation set that will be used at each added validation point. Through theoretical reasoning, we show that *n_j_* should decrease as *j* increases. For this purpose, exponential decay works better than linear decay. *V*′ is chosen to control the total overhead of computing the model’s error rate at the validation points added before the first original validation point, while keeping the randomly sampled subset of the full validation set large enough for reasonably estimating the model’s generalization error at each added validation point.

**FIGURE 2.**
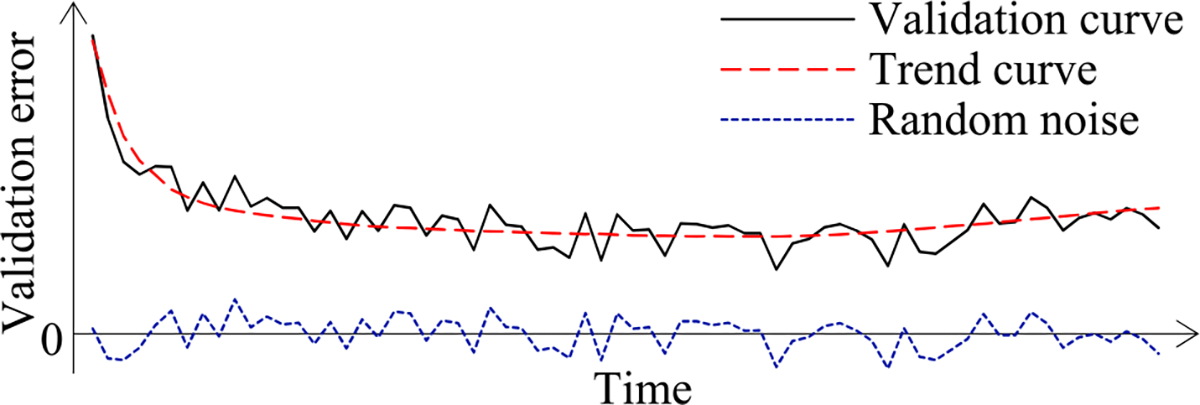
The validation curve = some random noise + a trend curve.

Second, the validation error is the model’s error rate calculated on the actual validation set used at a validation point. As in our prior paper [8], we use the validation curve to predict when early stopping will occur. As shown in Fig. 2, this curve shows the validation errors obtained over time, is non-smooth, and can be regarded as the sum of some zero-mean random noise and a smooth trend curve. The random noise’s variance depends on the size of the actual validation set used at the validation point. The relationship between these two numbers is previously unknown and difficult to be derived directly. However, we need to know this relationship in order to use both the original and the added validation points to predict when early stopping will occur. Noting that the random noise’s variance is equal to the validation error’s variance, we use an indirect approach to derive this relationship. We first compute the conditional mean and the conditional variance of the validation error given the model’s generalization error [12], both of which can be expressed using the model’s generalization error and the size of the actual validation set used at the validation point. Then we use the conditional mean, the conditional variance, and the law of total variance [13] to compute the validation error’s variance, which is expressed using the mean and the variance of the model’s generalization error and the size of the actual validation set used at the validation point.

Third, using the above-mentioned relationship and maximum likelihood estimation [13], we estimate the trend curve and the variance of the random noise. To the best of our knowledge, this is the first time that maximum likelihood estimation is employed for progress indication. The likelihood function is the product of multiple integrals, which are difficult to be used directly for numerical optimization. To overcome this hurdle, for each integral, we use the probability density function of a normal distribution to approximate a key component of the integrand. In this way, we acquire a simplified form of the likelihood function, which is easy to use for numerical optimization.

We implemented our new progress indication method in TensorFlow [14], an open-source software package for deep learning. We present our performance test results for recurrent and convolutional neural networks. Our results show that compared with using our prior method, using this new method reduces the progress indicator’s prediction error of the model construction time left by 57.5% on average. Also, with a low overhead, this new method enables us to obtain relatively accurate progress estimates faster.

### C. ORGANIZATION OF THE PAPER

The remaining sections of this paper are organized in the following way. Section II reviews our prior progress indication method for constructing deep learning models. Section III describes our new progress indication method for constructing deep learning models. Section IV shows performance test results by implementing our new method in TensorFlow. Section V presents the related work. Section VI points out some directions for future work. Section VII gives the conclusion.

## II. REVIEW OF OUR PRIOR PROGRESS INDICATION METHOD FOR CONSTRUCTING DEEP LEARNING MODELS

In this section, we first introduce some notations and concepts that will be used in the rest of the paper. Then we outline our prior progress indication method for constructing deep learning models. Finally, we compare our prior and our new progress indication methods.

### A. SOME NOTATIONS AND CONCEPTS

To control model construction, the user of the deep learning software specifies an early stopping condition and three positive integers *B*, *g*, and *m_e_*. During model construction, we process all the training instances for one or more rounds, also known as epochs. The deep learning model is constructed in batches, each processing *B* training instances to calculate parameter value updates to the model. We reach an original validation point after finishing every *g* batches of model construction. There, we first calculate the validation error, which is the model’s error rate on the full validation set. Then we assess whether the early stopping condition is fulfilled. If so, we end model construction. *m_e_* denotes the largest number of epochs permitted to train the model. If the early stopping condition remains unfulfilled by the time we finish the *m_e_*-th epoch, we end model construction at that time. Thus, the largest number of batches permitted to train the model is

$$b_{max}=\text{the}\;\text{count}\;\text{of}\;\text{data}\;\text{instances}\;\text{that}\;\text{are}\;\;\text{in}\;\text{the}\;\text{training}\;\text{set}\;\times \;m_{e}/B.$$

The largest number of original validation points permitted to train the model is

$$v_{max}=\left\lfloor b_{max}/g\right\rfloor ,$$

where ⌊ ⌋ is the floor function, e.g., ⌊4.4⌋ = 4.

As in our prior work [8], the goal of this work is not to deal with every early stopping condition that exists. Instead, we focus on a commonly used early stopping condition [1], [15] adopted in our prior work [8]. Through a case study on the condition, we demonstrate that when early stopping is permitted in constructing a deep learning model, it is feasible to obtain relatively accurate progress estimates faster by judiciously inserting extra validation points between the original validation points. The early stopping condition uses two pre-determined numbers: patience *p* > 0 and min_delta *δ* ≥ 0. The condition is fulfilled when the validation error drops by < *δ* for *p* original validation points in a row. In other words, letting ${\tilde{e}}_{j}$ denote the validation error of the model at the *j*-th original validation point, we end model construction at the *k*-th original validation point when ${\tilde{e}}_{k-p}-{\tilde{e}}_{i}$ is < *δ* for each *i* between *k* − *p* + 1 and *k*.

### B. OUTLINE OF OUR PRIOR PROGRESS INDICATION METHOD FOR CONSTRUCTING DEEP LEARNING MODELS

In this section, we outline our prior progress indication method. We begin with a crude estimate of the model construction cost. The estimated model construction cost is measured by the unit of work *U*. Every *U* is the mean quantity of work taken to process a training instance once in model construction, by going once forward and once backwards over the neural network. During model construction, we keep collecting statistics and using them to refine the estimated model construction cost. We keep monitoring the present model construction speed, which is calculated as the number of *U*s done per second in the past 10 seconds. The model construction time left is predicted to be the estimated model construction cost left divided by the present model construction speed. Every few seconds, the progress indicator is updated with the most recent information. As we keep collecting more precise information of the model construction task as it runs, our progress estimates are inclined to become more and more accurate.

#### 1) CALCULATING THE MODEL CONSTRUCTION COST

The model construction cost is predominated by and is approximately the sum of the cost to process the training instances and the cost to calculate the validation errors. The cost to process the training instances is

(1) =thecountofbatchesrequiredtotrainthemodel×thecountoftraininginstancesineverybatch×themeanquantityofworktakentoprocessatraininginstanceonetimeinmodelconstruction=thecountofbatchesrequiredtotrainthemodel×B.

Let *V* denote the count of data instances that are in the full validation set. Every data instance in the full validation set is called a validation instance. Our prior work [8] shows that the mean quantity of work taken to process a validation instance one time to calculate the validation error is 1/3 unit of work. The cost to calculate the validation errors is

(2) =thecountoforiginalvalidationpointsrequiredtotrainthemodel×thecountofdatainstancesthatareinthefullvalidationset×themeanquantityofworktakentoprocessavalidationinstanceonetimetocalculatethevalidationerror=thecountoforiginalvalidationpointsrequiredtotrainthemodel×V/3.

Let *n_v_* denote the count of original validation points required to train the model. Recall that *v_max_* denotes the largest number of original validation points permitted to train the model. *g* denotes the count of batches of model construction between two successive original validation points. If *n_v_* is < *v_max_*, early stopping will occur before we reach the *v_max_*-th original validation point. In this case, the count of batches required to train the model is = *n_v_* × *g*. If *n_v_* is = *v_max_*, early stopping will never occur. In this case, the count of batches required to train the model is = *b_max_*, the largest number of batches permitted to train the model. In formulas (1) and (2), *B*, *g*, and *V* are known before model construction starts. Hence, to predict the model construction cost, we mainly need to project *n_v_*.

#### 2) ESTIMATING THE COUNT OF ORIGINAL VALIDATION POINTS REQUIRED TO TRAIN THE MODEL

When model construction starts, we project *n_v_*, the count of original validation points required to train the model, to be *v_max_*, the largest number of original validation points permitted to train the model. After model construction starts, we use the validation curve to revise the estimated *n_v_*. We deem the validation curve to be the sum of some zero-mean random noise and a smooth trend curve (see Fig. 2). We use an inverse power function

$$f(j)=aj^{-b}+c[6],\;[16]-[19]$$

as the regression function to estimate the trend curve. Here, *a* is >0, *b* is >0, *c* is >0, and *j* is the original validation point’s sequence number. Since at least three data points are needed to estimate the three parameters *a*, *b*, and *c*, we do not refine the estimated *n_v_* before reaching the third original validation point. At each original validation point whose sequence number is ≥3 and at which the early stopping condition is unfulfilled, we re-estimate *n_v_* by fitting the regression function to the validation curve obtained so far, using recorded data to estimate the variance of the random noise, using the fitted regression function to estimate the trend curve for future original validation points, and then performing Monte Carlo simulation to project *n_v_*. During the Monte Carlo simulation, we create multiple synthetic validation curves through adding to the estimated trend curve simulated random noise. We apply the early stopping condition to every synthetic validation curve to obtain a separate simulated count of original validation points required to train the model. No simulated number can be > *v_max_*. Then we compute a revised estimate of *n_v_* based upon the estimated mode of these simulated numbers.

### C. COMPARING OUR PRIOR AND OUR NEW PROGRESS INDICATION METHODS

Tables 1 and 2 show the differences and the commonalities between our prior and our new progress indication methods for constructing deep learning models, respectively.

## III. OUR NEW PROGRESS INDICATION METHOD FOR CONSTRUCTING DEEP LEARNING MODELS

In this section, we present our new progress indication method for constructing deep learning models. Our presentation focuses on using deep learning for classification and the steps related to estimating the trend curve, the variance of the random noise, and the model construction cost based upon the predicted count of original validation points required to train the model. The approaches to conduct Monte Carlo simulation to estimate the count of original validation points required to train the model, to monitor the present model construction speed, and to estimate the model construction time left based upon the projected model construction cost left and the present model construction speed are identical to those used in our prior progress indication method for constructing deep learning models [8] and are omitted.

This section is organized in the follow way. Section III-A provides an overview of our new progress indication method for constructing deep learning models. Section III-B presents our approach to insert extra validation points between the original validation points. Section III-C shows how to set *V*′, the uniform size of the randomly sampled subset of the full validation set that will be used at each added validation point. Section III-D derives the relationship between the random noise’s variance and the size of the actual validation set used at the validation point. Section III-E shows how to estimate the trend curve and the variance of the random noise for future validation points. Section III-F describes how to determine *V_min_*, the minimum size needed for the randomly sampled subset of the full validation set used at an added validation point. Section III-G shows how to estimate the model construction cost based upon the predicted count of original validation points required to train the model.

In the rest of this paper, whenever we mention validation points, we mean both original and added validation points, unless original validation points or added validation points are explicitly mentioned.

### A. OVERVIEW OF THE NEW PROGRESS INDICATION METHOD

This section provides an overview of the new progress indication method for constructing deep learning models. To obtain relatively accurate progress estimates faster, we judiciously insert extra validation points between the original validation points. Using the validation errors obtained at both the original and the added validation points that we have encountered so far, we revise the predicted model construction cost at both the original and the added validation points. Consequently, compared with our prior progress indication method [8], our new progress indication method starts revising the predicted model construction cost earlier and revises the predicted model construction cost more frequently. This helps the progress indicator reduce its prediction error of the model construction time left and obtain relatively accurate progress estimates faster.

Our prior progress indication method [8] roughly approximates the model construction cost as the sum of two components: the cost to process the training instances and the cost to calculate the validation errors at the original validation points. In addition to these two components, our new progress indication method adds a third component to the model construction cost: the cost to calculate the validation errors at the added validation points. Our discussion of the model construction cost focuses on these three dominating components.

As in our prior work [8], to predict the model construction cost, we mainly need to predict *n_v_*, the count of original validation points required to train the model. When model construction starts, we estimate *n_v_* to be *v_max_*, the largest number of original validation points permitted to train the model. We deem the validation curve to be the sum of some zero-mean random noise and a smooth trend curve. Our new progress indication method uses four parameters to estimate the trend curve and the variance of the random noise (see Section III–E). Since at least *τ_v_* = 4 data points are needed to estimate the four parameters, we refine the estimated *n_v_* only when we reach a validation point whose sequence number is ≥ *τ_v_* and where the early stopping condition is unfulfilled.

A good progress indicator should have a low run-time overhead [6]. In our new progress indication method, a large part of the progress indicator’s run-time overhead comes from computing the model’s error rate at the added validation points. To lower this part of the run-time overhead, at each added validation point, we calculate the model’s error rate on a randomly sampled subset of the full validation set rather than on the full validation set. The sampling is done without replacement. The subset is usually much smaller than the full validation set and could be biased. If we keep using the same biased subset at each added validation point, the bias could have a large negative impact on our estimation accuracy of the trend curve, the variance of the random noise, and subsequently the model construction cost. To address this issue, we re-sample the full validation set to obtain a new subset at each added validation point to calculate the model’s error rate. Each subset includes the same number *V*′ of data instances. At each original validation point, we use the full validation set to calculate the model’s error rate.

The random noise’s variance depends on the size of the actual validation set used at the validation point. We use an indirect approach to derive the relationship between these two numbers. Using this relationship, the validation curve obtained so far, and maximum likelihood estimation [13], we estimate the trend curve and the variance of the random noise for future validation points. We use the Monte Carlo simulation approach in our prior work [8] to predict *n_v_*, the count of original validation points required to train the model. Finally, we revise the predicted model construction cost based upon the projected *n_v_*.

### B. OUR APPROACH TO INSERT EXTRA VALIDATION POINTS BETWEEN THE ORIGINAL VALIDATION POINTS

This section describes our approach to insert extra validation points between the original validation points. We regard the beginning of model construction as the 0-th original validation point, although the model’s error rate is not computed there. For each pair of successive original validation points, we insert extra validation points evenly between them. More specifically, recall that *g* denotes the count of batches of model construction between two successive original validation points. *v_max_* denotes the largest number of original validation points permitted to train the model. *n_j_* (0 ≤ *j* ≤ *v_max_* − 1) denotes the count of validation points to be added between the *j*-th and the (*j* + 1)-th original validation points. When *j* = 0, *n*_0_ denotes the count of validation points to be added before the first original validation point. We ensure that *n_j_* is ≤ *g* − 1 for every *j* between 0 and *v_max_* − 1. Starting from the *j*-th original validation point, we do

$$\lfloor kg/\left(n_{j}+1\right)\rceil$$

batches of model construction to reach the *k*-th (1 ≤ *k* ≤ *n_j_*) of the *n_j_* validation points added between the *j*-th and the (*j* + 1)-th original validation points. Here, ⌊ ⌉ is the nearest integer function, e.g., ⌊4.4⌉ = 4 and ⌊4.6⌉ = 5.

The rest of this section is organized in the following way. Section III-B1 provides an overview of how we set *n_j_* (0 ≤ *j* ≤ *v_max_* − 1), the count of validation points to be added between the *j*-th and the (*j* + 1)-th original validation points. Section III-B2 describes how to set *n*_0_, the count of validation points to be added before the first original validation point. Section III-B3 shows how to set *q*, the constant regulating the decay rate of *n_j_* (0 ≤ *j* ≤ *v_max_* − 1) in the exponential decay schema.

#### 1) OVERVIEW OF HOW WE SET *n_j_* (0 ≤ *j* ≤ *v_max_* − 1)

This section provides an overview of how we set *n_j_* (0 ≤ *j* ≤ *v_max_* − 1), the count of validation points to be added between the *j*-th and the (*j* + 1)-th original validation points.

Recall that *n_v_* denotes the count of original validation points required to train the model. Our initial estimate of *n_v_* is usually inaccurate and is not refined until we reach the fourth validation point. As we accumulate more data points over time, our estimate of *n_v_* tends to become more accurate. To refine our initial estimate of *n_v_* as soon as possible and to obtain relatively accurate estimates of *n_v_* faster, we insert more validation points for use at the early stages of model construction than at the later stages of model construction. In other words, we decrease *n_j_* (0 ≤ *j* ≤ *v_max_* − 1), the count of validation points to be added between the *j*-th and the (*j* + 1)-th original validation points, as *j* increases. Furthermore, we want *n*_0_, the count of validation points to be added before the first original validation point, to be reasonably large. This is particularly the case when a sophisticated progress indicator is most needed: the training set is large, many batches of model construction are performed between two successive validation points, and model construction takes a long time.

One could decrease *n_j_* either linearly or exponentially as *j* increases. For our purpose, exponential decay works better than linear decay. To compare these two decay schemata of *n_j_* and show this, we consider two model construction processes that have the same setting except for the decay schema used. Recall that *n*_0_ denotes the count of validation points to be added before the first original validation point. *v_max_* is the largest number of original validation points permitted to train the model. One model construction process uses the exponential decay schema, where

$$n_{j}=\lfloor n_{0}q^{j}\rceil \;\left(1\leq j\leq v_{max}-1\right),$$

*q* (0 ≤ *q* < 1) is a constant regulating the decay rate of *n_j_*, and 0^0^ is defined to be 1. The other model construction process uses the linear decay schema, where

$$n_{j}=\max\left(\lfloor n_{0}-jz\rceil ,\;0\right)\;\left(1\leq j\leq v_{max}-1\right)$$

and *z* is a constant >0 regulating the decay rate of *n_j_*. Given the same mean cost of calculating the validation error at each added validation point, the total cost of calculating the validation errors at all added validation points is ∝ the total count of validation points added between the original validation points. To have the same total cost of calculating the validation errors at all added validation points, in the two model construction processes we insert the same total number of validation points between the original validation points. For a sufficiently large *v_max_*, the total count of validation points added between the original validation points is roughly

$$\sum_{j=0}^{+\infty }n_{0}q^{j}=n_{0}/(1-q)$$

and

$$\sum_{j=0}^{\left\lfloor n_{0}/z\right\rfloor }\left(n_{0}-jz\right)\approx n_{0}^{2}/(2z)$$

for the exponential decay schema and the linear decay schema, respectively. Recall that we want *n*_0_ to be reasonably large. Thus, we expect the *n*_0_ used in the linear decay schema to be typically >2*z*/(1 − *q*). In this case, the *n*_0_ used in the exponential decay schema is larger than the *n*_0_ used in the linear decay schema. Adopting a larger *n*_0_ makes the early stage of model construction include more added validation points, which is what we want. Thus, we employ the exponential decay schema instead of the linear decay schema. In the exponential decay schema, once *n*_0_ and *q* are set using the approach given in Sections III-B2 and III-B3, respectively, *n_j_* is known for each *j* between 0 and *v_max_* − 1.

#### 2) SETTING *n*_0_

In this section, we describe how to set *n*_0_, the count of validation points to be added before the first original validation point. When setting *n*_0_, we try to fulfill the following two requirements if possible:

##### 1) Requirement 1:

When we finish the work at the fourth validation point, the model construction cost that has been incurred is ≤ *C* units of work, where *C* is a pre-set number >0. Requirement 1 is used to control the amount of time that elapses before we refine our beginning estimate of the model construction cost for the first time at the fourth validation point. This amount should not be too large.

##### 2) Requirement 2:

From when model construction starts to the time we finish the work at the first original validation point, the cost to calculate the validation errors at the added validation points is ≤ *c*_0_*P*_1_. Here, *P*_1_ is a pre-set percentage >0. *c*_0_ denotes the model construction cost that has been incurred when we finish the work at the first original validation point, excluding the progress indicator’s overhead of calculating the validation errors at the added validation points. That is, *c*_0_ is = the cost to process the training instances before we reach the first original validation point + the cost to calculate the validation error at the first original validation point. Requirement 2 is used to control the progress indicator’s overhead that has been incurred for calculating the validation errors at the added validation points when we finish the work at the first original validation point. This overhead should not be too large.

These two requirements are soft requirements, as it may not always be possible to fully fulfill both requirements.

We have two considerations when setting the value of *C* in Requirement 1. On one hand, to prevent the user of the deep learning software from waiting too long before our beginning estimate of the model construction cost is refined for the first time at the fourth validation point, we do not want *C* to be too large. On the other hand, the smaller the *C*, the more validation points need to be added before the first original validation point, and subsequently due to Requirement 2, the smaller the cost of calculating the validation error at an added validation point can be. At each added validation point, the cost to calculate the validation error is ∝ the size of the randomly sampled subset of the full validation set used to calculate the model’s error rate. If *C* is too small, this subset will not be large enough for reasonably estimating the model’s generalization error. This will lower the progress indicator’s projection accuracy of the model construction cost and is undesirable. To strike a balance between the two considerations, we set *C*’s default value to 20,000 × the number of GPUs, TPUs, or central processing units (CPUs) used to train the model. This allows a non-trivial number of batches of model construction to appear between two successive validation points, as a batch of model construction typically involves much <20,000/4 = 5,000 units of work on any GPU, TPU, or CPU.

We have two considerations when setting the value of *P*_1_ in Requirement 2. On one hand, we want *P*_1_ to be small so that the progress indicator does not cause a large increase in the model construction cost during the period from when model construction starts to the time we finish the work at the first original validation point. On the other hand, if *P*_1_ is too small, at each added validation point, the randomly sampled subset of the full validation set used to calculate the model’s error rate will not be large enough for reasonably estimating the model’s generalization error. This is undesirable. There is also no need to make *P*_1_ too small. Recall that *n_j_* (0 ≤ *j* ≤ *v_max_* − 1) denotes the count of validation points to be added between the *j*-th and the (*j* + 1)-th original validation points. As *n_j_* decreases as *j* increases, the progress indicator’s overhead of calculating the validation errors at the validation points added before the first original validation point can be amortized over time during model construction. To strike a balance between the two considerations, we set the default value of *P*_1_ to 5%.

Recall that *c*_0_ is the model construction cost that has been incurred when we finish the work at the first original validation point, excluding the progress indicator’s overhead of calculating the validation errors at the added validation points. *n*_0_ denotes the count of validation points to be added before the first original validation point. We first compute *c*_0_ and then decide the value of *n*_0_.

###### a: COMPUTING c_0_

Recall that *g* denotes the count of batches of model construction between two successive original validation points. *B* is the count of training instances in every batch. *c*_0_ is the sum of two parts. The first part is the cost to process the training instances before we reach the first original validation point

=thecountofbatchesofmodelconstructionbeforethefirstoriginalvalidationpoint×thecountoftraininginstancesineverybatch×themeanquantityofworktakentoprocessatraininginstanceonetimeinmodelconstruction=g×B×1=gB.

Our prior work [8] shows that the mean quantity of work taken to process a validation instance one time to calculate the validation error is 1/3 unit of work. Recall that *V* is the count of data instances that are in the full validation set. The second part of *c*_0_ is *c_v_*, the cost to calculate the validation error at the first original validation point. *c_v_* is

=thecountofdatainstancesthatareinthefullvalidationset×themeanquantityofworktakentoprocessavalidationinstanceonetimetocalculatethevalidationerror=V/3.

Adding the two components, we have *c*_0_ = *gB* + *V*/3.

###### b: DECIDING THE VALUE OF n_0_

Recall that *c*_0_ is the model construction cost that has been incurred when we finish the work at the first original validation point, excluding the progress indicator’s overhead of calculating the validation errors at the added validation points. *P*_1_ is the maximum allowed percentage increase in the model construction cost that the progress indicator causes during the period from when model construction starts to the time we finish the work at the first original validation point. *C* is the upper threshold of the model construction cost that has been incurred when we finish the work at the fourth validation point. *c_v_* is the cost to calculate the validation error at the first original validation point. *n*_0_ denotes the count of validation points to be added before the first original validation point.

**FIGURE 3.**
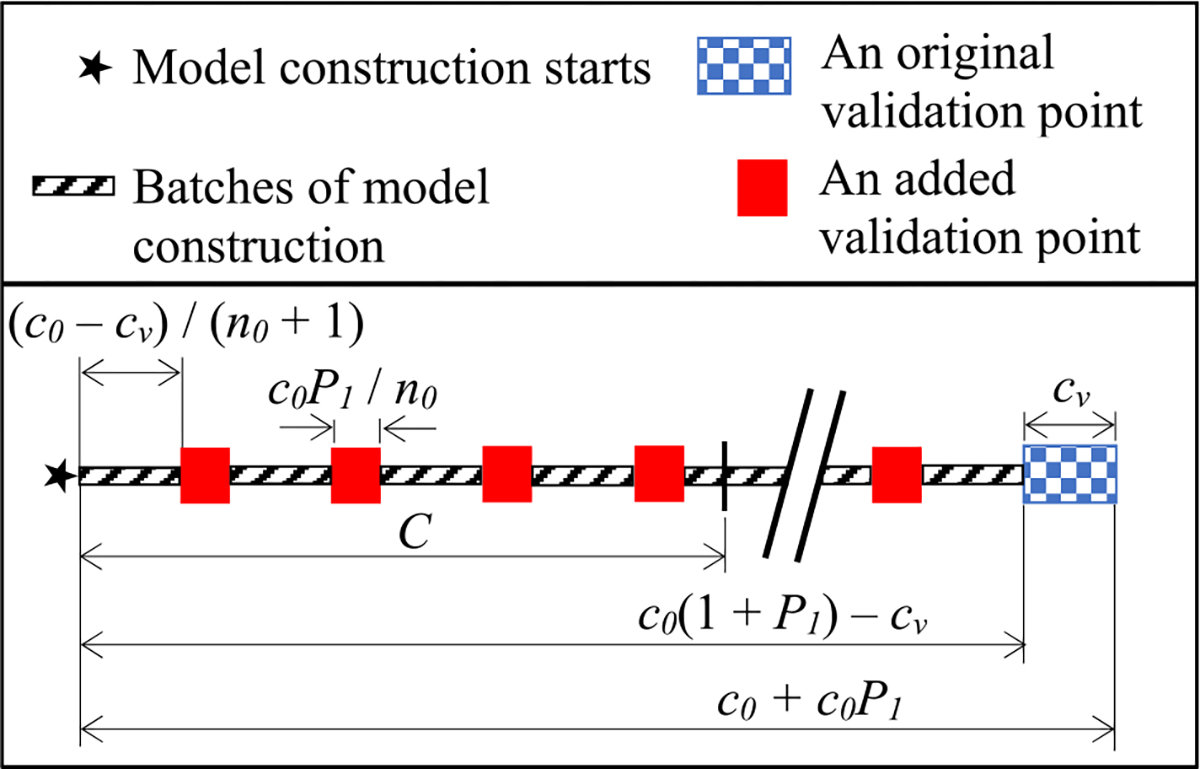
Decomposition of the model construction cost that has been incurred when we finish the work at the first original validation point.

When setting *n*_0_, we try to fulfill Requirements 1 and 2 mentioned above if possible. In attempting to fulfill Requirement 2, we can aim the cost to calculate the validation errors at the *n*_0_ validation points added before the first original validation point to be *c*_0_*P*_1_. There are two possible cases:

1) Case 1: The model construction cost that has been incurred when we are just about to arrive at the first original validation point is ≥ *C* (see Fig. 3). That is,


$$c_{0}+c_{0}P_{1}-c_{v}=c_{0}\left(1+P_{1}\right)-c_{v}\;\;\geq C.$$

In this case, we show that if *n*_0_ is set to

$$\left\lceil 4\left[c_{0}\left(1+P_{1}\right)-c_{v}\right]/C\right\rceil$$

that is ≥4, Requirement 1 is fulfilled. Here, ⌈ ⌉ is the ceiling function, e.g., ⌈4.4⌉ = 5. We note that:

- The cost to calculate the validation error at each of the *n*_0_ validation points added before the first original validation point is *c*_0_*P*_1_/*n*_0_.
- The cost to process the training instances that has been incurred when we are just about to arrive at the first original validation point is *c*_0_ − *c_v_*, which is >0. With *n*_0_ validation points inserted before it, the first original validation point is the (*n*_0_+1)-th validation point. Thus, before we finish the work at the first original validation point, the cost to process the training instances between two successive validation points is (*c*_0_ − *c_v_*)/(*n*_0_ + 1).

The fourth validation point is the fourth validation point added before the first original validation point. The model construction cost that has been incurred when we finish the work at the fourth validation point is the sum of two components:

- 4*c*_0_*P*_1_/*n*_0_, the cost to calculate the validation errors at the first four validation points added before the first original validation point; and
- 4(*c*_0_ −*c_v_*)/(*n*_0_ +1), the cost to process the training instances before we reach the fourth validation point.

Adding these two components, we get the model construction cost that has been incurred when we finish the work at the fourth validation point

$$=4c_{0}P_{1}/n_{0}+4\left(c_{0}-c_{v}\right)/\left(n_{0}+1\right)\;<4\;c_{0}P_{1}/n_{0}+4\left(c_{0}-c_{v}\right)/n_{0}\;=4\left[c_{0}\left(1+P_{1}\right)-c_{v}\right]/n_{0}\;=C\times 4\left[c_{0}\left(1+P_{1}\right)-c_{v}\right]/C/\left\lceil 4\left[c_{0}\left(1+P_{1}\right)-c_{v}\right]/C\right\rceil \;\leq C.$$

This verifies that Requirement 1 is fulfilled.

2) Case 2: The model construction cost that has been incurred when we are just about to arrive at the first original validation point is < *C*. That is,


$$c_{0}\left(1+P_{1}\right)-c_{v}<C.$$

In this case, if *n*_0_ is set to 4, the fourth validation point is the fourth validation point added before the first original validation point. The model construction cost that has been incurred when we finish the work at the fourth validation point is < that when we are just about to arrive at the first original validation point, and thus is < *C*. This shows that Requirement 1 is fulfilled.

Recall that *g* denotes the count of batches of model construction between two successive original validation points. At least one batch of model construction needs to occur between two successive validation points. Thus, *n*_0_ cannot exceed *g* − 1. To fulfill this, we set *n*_0_ to

$$\min\left(\left\lceil 4\left[c_{0}\left(1+P_{1}\right)-c_{v}\right]/C\right\rceil ,\;g-1\right)$$

if *c*_0_(1 + *P*_1_) − *c_v_* is ≥ *C*. Otherwise, if *c*_0_(1 + *P*_1_) − *c_v_* is < *C*, we set *n*_0_ to min(4, *g* − 1).

#### 3) SETTING *q*

In this section, we show how to set *q*, the constant regulating the decay rate of *n_j_* (0 ≤ *j* ≤ *v_max_* − 1) in the exponential decay schema. Recall that *v_max_* denotes the largest number of original validation points permitted to train the model. *n_j_* (0 ≤ *j* ≤ *v_max_* − 1) denotes the count of validation points to be added between the *j*-th and the (*j*+1)-th original validation points. *n*_0_ denotes the count of validation points to be added before the first original validation point. In the exponential decay schema, *n_j_* = ⌊*n*_0_*q^j^*⌉ (0 ≤ *j* ≤ *v_max_* − 1).

Let *p_j_* (1 ≤ *j* ≤ *v_max_*) denote the percentage increase in the model construction cost that the progress indicator causes during the period from when model construction starts to the time we finish the work at the *j*-th original validation point. When setting *q*, we try to fulfill the following requirement if possible:

##### Requirement 3:

*p_vmax_* is ≤ *P_v_*, where *P_v_* is a pre-set percentage >0.

This requirement is a soft requirement, as it may not always be possible to fully fulfill this requirement.

The increase in the model construction cost caused by the progress indicator comes from calculating the validation errors at the added validation points. Since the same number of validation instances are used to calculate the validation error at each added validation point, the cost to calculate the validation error at an added validation point is a constant. Thus, during the period from when model construction starts to the time we finish the work at the *j*-th (1 ≤ *j* ≤ *v_max_*) original validation point, the increase in the model construction cost caused by the progress indicator is $\propto \sum_{k=0}^{j-1}n_{k}$, the total count of validation points added before the *j*-th original validation point. During the same period, the model construction cost excluding the progress indicator’s overhead of calculating the validation errors at the added validation points is ∝ *j*, as both the cost to process the training instances between two successive original validation points and the cost to calculate the validation error at an original validation point are constants. As the ratio of the increase in the model construction cost caused by the progress indicator to the model construction cost excluding the progress indicator’s overhead, *p_j_* (1 ≤ *j* ≤ *v_max_*) is

(3) $$\propto \sum_{k=0}^{j-1}n_{k}/j\;=\sum_{k=0}^{j-1}\lfloor n_{0}q^{k}\rceil /j.$$

As *j* increases, *n_j_* and subsequently *p_j_* strictly decrease. Thus, *P_v_* in Requirement 3 should be < *P*_1_, the maximum allowed percentage increase in the model construction cost that the progress indicator causes during the period from when model construction starts to the time we finish the work at the first original validation point. In addition, we have two other considerations when setting the value of *P_v_*. On one hand, we want *P_v_* to be small, as a good progress indicator should have a low run-time overhead [6]. On the other hand, the larger the *P_v_*, the more validation points we can add before model construction finishes. This helps us obtain more accurate progress estimates for the model construction process. To strike a balance between these two considerations, we set the default value of *P_v_* to 0.5%.

Recall that when deciding the value of *n*_0_, we aim *p*_1_ to be = *P*_1_ in attempting to fulfill Requirement 2. In the following derivation used to set *q*, we regard *p*_1_ to be = *P*_1_. There are two possible cases: 1) *v_max_* is < *P*_1_/*P_v_* and 2) *v_max_* is ≥ *P*_1_/*P_v_*. We discuss the two cases sequentially.

###### Case 1 (v_max_ is < P_1_/P_v_)

We first discuss the case when *v_max_* is < *P*_1_/*P_v_*. Recall that *v_max_* denotes the largest number of original validation points permitted to train the model. *n_j_* (0 ≤ *j* ≤ *v_max_* − 1) denotes the count of validation points to be added between the *j*-th and the (*j*+1)-th original validation points. *q* (0 ≤ *q* < 1) is the constant regulating the decay rate of *n_j_* in the exponential decay schema. *P*_1_ is the maximum allowed percentage increase in the model construction cost that the progress indicator causes during the period from when model construction starts to the time we finish the work at the first original validation point. *p_j_* (1 ≤ *j* ≤ *v_max_*) is the percentage increase in the model construction cost that the progress indicator causes during the period from when model construction starts to the time we finish the work at the *j*-th original validation point. We regard *p*_1_ to be = *P*_1_.

Formula (3) shows that *p_j_* (1 ≤ *j* ≤ *v_max_*) is

$$\propto \sum_{k=0}^{j-1}\lfloor n_{0}q^{k}\rceil /j.$$

For *j* = *v_max_*, we have

$$p_{v_{max}}\propto \sum_{k=0}^{v_{max}-1}\lfloor n_{0}q^{k}\rceil /v_{max}.$$

For *j* = 1, we have

$$p_{1}\propto n_{0}/1.$$

When *q* is 0, $p_{v_{max}}$ reaches its smallest value, which is ∝ *n*_0_/*v_max_* and is = *p*_1_/*v_max_* = *P*_1_/*v_max_*. When *v_max_* is < *P*_1_/*P_v_*, $p_{v_{max}}$ must be > *P_v_*. Requirement 3 cannot be fully fulfilled. To minimize $p_{v_{max}}$ and fulfill Requirement 3 as much as possible, we set *q* to 0.

###### Case 2 (v_max_ is ≥ P_1_/P_v_)

Next, we discuss the case when *v_max_* is ≥ *P*_1_/*P_v_*. When *v_max_* is = *P*_1_/*P_v_*, we set *q* to 0 to let $p_{v_{max}}$ reach its smallest value *P*_1_/*v_max_* = *P_v_* and fulfill Requirement 3. When *v_max_* is > *P*_1_/*P_v_*, we proceed as follows.

Formula (3) shows that *p_j_* (1 ≤ *j* ≤ *v_max_*) is

(4) $$\propto \sum_{k=0}^{j-1}\lfloor n_{0}q^{k}\rceil /j\;\approx \sum_{k=0}^{j-1}n_{0}q^{k}/j.$$

For *j* = *v_max_*, we roughly have

(5) $$p_{v_{max}}\propto \sum_{k=0}^{v_{max}-1}n_{0}q^{k}/v_{max}.$$

For *j* = 1, we have

(6) $$p_{1}\propto n_{0}/1.$$

Dividing each side of formula (5) by the corresponding side of formula (6), we roughly have

(7) $$p_{v_{max}}/p_{1}=\sum_{k=0}^{v_{max}-1}q^{k}/v_{max}.$$

Regarding *p*_1_ to be = *P*_1_ and rearranging formula (7) lead to

$$\sum_{k=0}^{v_{max}-1}q^{k}-v_{max}p_{v_{max}}/P_{1}=0.$$

If we make the function of *q*

$$f(q)\overset{\;\text{def}\;}{=}\sum_{k=0}^{v_{max}-1}q^{k}-v_{max}P_{v}/P_{1}\;\;=0,$$

we can have $p_{v_{max}}=P_{v}$ and fulfill Requirement 3. Recall that *P*_1_ > *P_v_* > 0. The following theorem holds.

##### Theorem:

For any *v_max_* > *P*_1_/*P_v_*, *f* (*q*) must have a unique root *q* in (0, 1).

##### Proof:

For each *k* (1 ≤ *k* ≤ *v_max_* − 1), *q^k^* is continuous and strictly increasing on [0, 1]. Thus, *f* (*q*) is continuous and strictly increasing on [0, 1].

$$f(0)=1-v_{max}P_{v}/P_{1}$$

is <0 because *v_max_* is > *P*_1_/*P_v_*.

$$f(1)=v_{max}-v_{max}P_{v}/P_{1}$$

is >0 because *P*_1_ is > *P_v_*. According to the intermediate value theorem [20], *f* (*q*) must have a root in (0, 1). As *f* (*q*) is strictly increasing on [0, 1], this root is unique. ■

$$\text{For}\;\text{any}\;q\neq 1,\;f(q)\;\text{is}\;\;=\left(1-q^{v_{max}}\right)/(1-q)-v_{max}P_{v}/P_{1}.$$

We use the bisection method to find *f* (*q*)’s unique root in (0, 1) and set *q* to this root.

In summary, we set *q* to 0 if *v_max_* is ≤ *P*_1_/*P_v_*. Otherwise, if *v_max_* is > *P*_1_/*P_v_*, we set *q* to *f* (*q*)’s unique root in (0, 1).

###### The Shape of p_j_ as a Function of j

Recall that *p_j_* (1 ≤ *j* ≤ *v_max_*) strictly decreases as *j* increases. In this section, we show that *p_j_* decreases quickly as *j* increases, indicating that the progress indicator usually has a low run.time overhead.

When *v_max_* is ≤ *P*_1_/*P_v_*, *q* is set to 0. Formula (3) shows that *p_j_* (1 ≤ *j* ≤ *v_max_*) is

$$\propto \sum_{k=0}^{j-1}\lfloor n_{0}q^{k}\rceil /j\;=n_{0}/j.$$

For *j* = 1, we have

$$p_{1}\propto n_{0}/1.$$

Thus, *p_j_* = *p*_1_/*j*. This is a rapidly decreasing function of *j*. Typically, the patience *p* in the early stopping condition is ≥2. When the early stopping condition is fulfilled, we have encountered ≥3 original validation points (i.e., *j* ≥ 3) and *p_j_* is ≤5%/3 ≈ 1.7% if *p*_1_ is = *P*_1_ = 5%.

When *v_max_* is > *P*_1_/*P_v_*, *q* is set to a number in (0, 1). Formula (4) shows that *p_j_* (1 ≤ *j* ≤ *v_max_*) is roughly

$$\propto \sum_{k=0}^{j-1}n_{0}q^{k}/j\;=n_{0}\left(1-q^{j}\right)/(1-q)/j\;<n_{0}/(1-q)/j.$$

**FIGURE 4.**
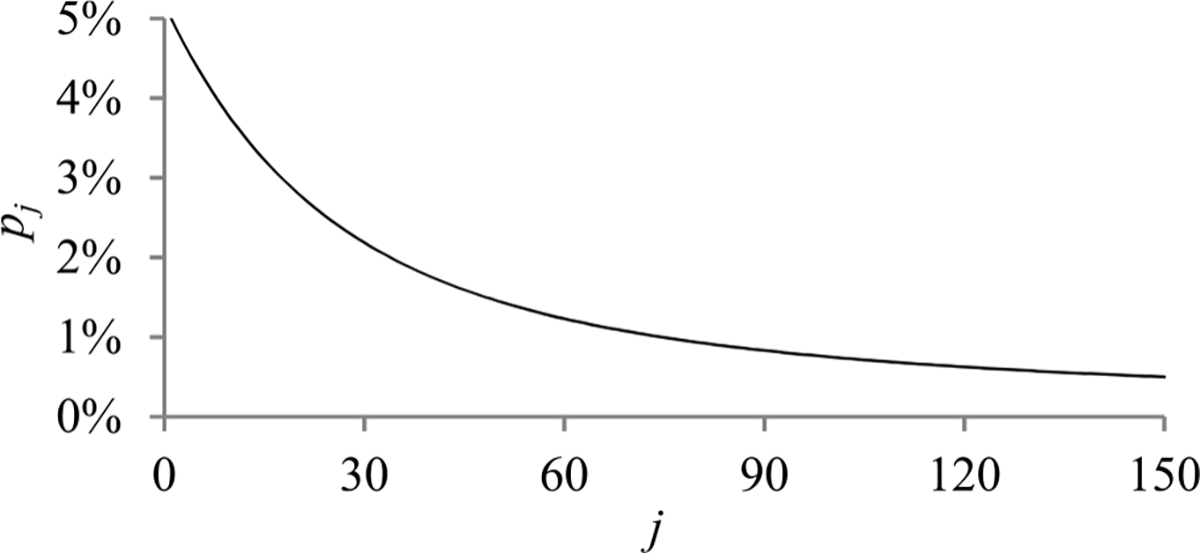
A typical shape of *p*_*j*_ as a function of *j*.

Since *p*_1_ is ∝ *n*_0_/1, *p_j_* decreases faster than *p*_1_*/*(1 − *q*)/*j* as *j* increases. Fig. 4 shows a typical shape of *p_j_* as a function of *j*.

### C. SETTING V′

At each added validation point, we use a distinct randomly sampled subset of the full validation set to calculate the model’s error rate. Every subset contains the same number of data instances. In this section, we show how to set *V*′, the count of data instances that are in the subset.

Our prior work [8] shows that the mean quantity of work taken to process a validation instance one time to calculate the validation error is 1/3 unit of work. The cost to calculate the validation errors at the *n*_0_ validation points added before the first original validation point is

$$=n_{0}\times \;\text{the}\;\text{count}\;\text{of}\;\text{data}\;\text{instances}\;\text{that}\;\text{are}\;\text{in}\;\text{the}\;\;\text{randomly}\;\text{sampled}\;\text{subset}\;\text{of}\;\text{the}\;\text{full}\;\text{validation}\;\;\text{set}\;\text{used}\;\text{at}\;\text{each}\;\text{added}\;\text{validation}\;\text{point}\;\;\times \;\text{the}\;\text{mean}\;\text{quantity}\;\text{of}\;\text{work}\;\text{taken}\;\text{to}\;\text{process}\;\;\text{a}\;\text{validation}\;\text{instance}\;\text{one}\;\text{time}\;\text{to}\;\;\text{calculate}\;\text{the}\;\text{validation}\;\text{error}\;=n_{0}V^{\prime }/3.$$

Recall that *c*_0_ is the model construction cost that has been incurred when we finish the work at the first original validation point, excluding the progress indicator’s overhead of calculating the validation errors at the added validation points. *P*_1_ is the maximum allowed percentage increase in the model construction cost that the progress indicator causes during the period from when model construction starts to the time we finish the work at the first original validation point. If we set

$$V^{\prime }=\lfloor c_{0}P_{1}/n_{0}/(1/3)\rceil \;\;=\lfloor 3c_{0}P_{1}/n_{0}\rceil ,$$

we have *n*_0_*V*′/3 ≈ *c*_0_*P*_1_ fulfilling Requirement 2.

As described in Sections III.E1 and III-F, our estimation method of the trend curve and the variance of the random noise requires *V*′ to be ≥ a threshold *V_min_*. This may occasionally cause Requirement 2 to be not fully fulfilled. Moreover, *V*′ should be ≤ *V*, the count of data instances that are in the full validation set. Given all the above considerations, we set

(8) $$V^{\prime }=\min\left(\max\left(\lfloor 3c_{0}P_{1}/n_{0}\rceil ,\;V_{min}\right),\;V\right).$$

### D. RELATIONSHIP BETWEEN THE RANDOM NOISE’S VARIANCE AND THE SIZE OF THE ACTUAL VALIDATION SET USED AT THE VALIDATION POINT

At each original validation point, the actual validation set used is the full validation set. At each added validation point, the actual validation set used is a randomly sampled subset of the full validation set. Recall that we deem the validation curve to be the sum of some zero-mean random noise and a smooth trend curve. The random noise’s variance depends on the size of the actual validation set used at the validation point. The relationship between these two numbers is previously unknown and difficult to be derived directly. However, we need to know this relationship in order to use both the original and the added validation points to predict when early stopping will occur. Noting that the random noise’s variance is equal to the validation error’s variance, we use an indirect approach to derive this relationship in two steps:

1. Step 1: Compute the conditional mean and the conditional variance of the validation error given the model’s generalization error [12], both of which can be expressed using the model’s generalization error and the size of the actual validation set used at the validation point.
2. Step 2: Use the conditional mean, the conditional variance, and the law of total variance [13] to compute the validation error’s variance, which is expressed using the mean and the variance of the model’s generalization error and the size of the actual validation set used at the validation point.

In the following, we first define a model’s generalization error and then present the two steps sequentially.

#### A Model’s Generalization Error

For a classification task, a model’s generalization error is defined as the probability that a data instance is misclassified by the model [12]. A deep learning model’s generalization error at any validation point is a random variable, as three factors introduce randomness into the model construction process. First, the model is trained in batches using stochastic gradient descent [1]. Each batch processes *B* training instances randomly chosen from the training set. Second, the weights of the neural network model are frequently randomly initialized [1]. Third, dropout [21] is often used in model construction. When using dropout, in every batch of model construction, we randomly omit some nodes along with their connections of the neural network model.

##### Step 1: Compute the conditional mean and the conditional variance of the validation error given the model’s generalization error

Let *V_j_* (*V_j_* ≥ 1) denote the count of data instances that are in the actual validation set used at the *j*-th validation point. If the *j*-th validation point is an original validation point, *V_j_* is = *V*, the count of data instances that are in the full validation set. If the *j*-th validation point is an added validation point, *V_j_* is = *V*′, the uniform number of data instances that are in the randomly sampled subset of the full validation set used at each added validation point. Let *e_j_* (0 ≤ *e_j_* ≤ 1) denote the model’s generalization error at the *j*-th validation point, *c_j_* denote the count of validation instances that are misclassified by the model and in the actual validation set used at the *j*-th validation point, and

(9) $${\hat{e}}_{j}=c_{j}/V_{j}\;\left(0\leq {\hat{e}}_{j}\leq 1\right)$$

denote the validation error of the model at the *j*-th validation point. As an estimate of *e_j_*, ${\hat{e}}_{j}$ is a discrete random variable.

A standard assumption used in machine learning is that all data instances are independently and identically sampled from an underlying distribution [12]. The probability that a data instance is misclassified by the model is *e_j_*. Given *e_j_*, *c_j_* follows a binomial distribution. Its probability mass function is

(10) $$P\left(c_{j}|e_{j}\right)=\left(\begin{array}{c} V_{j}\\ c_{j} \end{array}\right)e_{j}^{c_{j}}{\left(1-e_{j}\right)}^{V_{j}-c_{j}}.$$

The conditional mean and the conditional variance of *c_j_* given *e_j_* are *E*(*c_j_*|*e_j_*) = *V_j_e_j_* and *Var*(*c_j_*|*e_j_*) = *V_j_e_j_*(1 − *e_j_*), respectively. From formulas (9) and (10), we have

(11) $$E\left({\hat{e}}_{j}|e_{j}\right)=E\left(c_{j}|e_{j}\right)/V_{j}\;\;=e_{j}$$

and

(12) $$Var\left({\hat{e}}_{j}|e_{j}\right)=Var\left(c_{j}|e_{j}\right)/V_{j}^{2}\;\;=e_{j}\left(1-e_{j}\right)/V_{j}.$$

##### Step 2: Compute the validation error’s variance

Recall that *V_j_* (*V_j_* ≥ 1) denotes the count of data instances that are in the actual validation set used at the *j*-th validation point. ${\hat{e}}_{j}$ denotes the validation error of the model at the *j*-th validation point. *e_j_* denotes the model’s generalization error at the *j*-th validation point. Let *μ_j_* (0 ≤ *μ_j_* ≤ 1) and $\sigma_{j}^{2}$ denote the mean and the variance of *e_j_*, respectively. Given two random variables *X* and *Y*, the law of total variance [13] is

Var(X)=E[Var(X∣Y)]+Var[E(X∣Y)].

We have

(13) $$Var\left({\hat{e}}_{j}\right)=E\left[Var\left({\hat{e}}_{j}|e_{j}\right)\right]+Var\left[E\left({\hat{e}}_{j}|e_{j}\right)\right]\;\;=E\left[e_{j}\left(1-e_{j}\right)/V_{j}\right]+Var\left(e_{j}\right)\;\;\text{(plug}\;\text{in}\;\text{formulas}\;\text{(11)}\;\text{and}\;\text{(12))}\;\;=\left[E\left(e_{j}\right)-E\left(e_{j}^{2}\right)\right]/V_{j}+\sigma_{j}^{2}\;\;=\left[\mu_{j}-\left(Var\left(e_{j}\right)+E{\left(e_{j}\right)}^{2}\right)\right]/V_{j}+\sigma_{j}^{2}\;\;\left(\text{as}\;Var(X)=E\left(X^{2}\right)-E{\left(X\right)}^{2}\right)\;\;=\left(\mu_{j}-\sigma_{j}^{2}-\mu_{j}^{2}\right)/V_{j}+\sigma_{j}^{2}\;\;=\left(\mu_{j}-\mu_{j}^{2}\right)/V_{j}+\left(1-1/V_{j}\right)\sigma_{j}^{2}.$$

At the *j*-th validation point, the variance of the random noise is $=Var\left({\hat{e}}_{j}\right)$ computed by formula (13).

### E. ESTIMATING THE TREND CURVE AND THE VARIANCE OF THE RANDOM NOISE FOR FUTURE VALIDATION POINTS

Recall that we re-estimate the count of original validation points required to train the model only when we reach a validation point whose sequence number is ≥ *τ_v_* and where the early stopping condition is unfulfilled. In this section, we show at such a validation point, how to estimate the trend curve and the variance of the random noise for future validation points. To do this, we need to only estimate for each *j* ≥ 1, the mean *μ_j_* and the variance $\sigma_{j}^{2}$ of the model’s generalization error at the *j*-th validation point. Once *μ_j_* and $\sigma_{j}^{2}$ are obtained, the random noise’s variance at the *j*-th validation point can be computed by formula (13). Moreover, the trend curve’s value at the *j*-th validation point is = *μ_j_*. To show this, recall that ${\hat{e}}_{j}$ is the validation error of the model at the *j*-th validation point. *e_j_* is the model’s generalization error at the *j*-th validation point. We deem the validation curve to be the sum of some zero-mean random noise and a smooth trend curve. The trend curve’s value at the *j*-th validation point is $=E\left({\hat{e}}_{j}\right)$. Given two random variables *X* and *Y*, the law of total expectation [13] is

E(X)=E[E(X∣Y)].

We have

$$E\left({\hat{e}}_{j}\right)=E\left[E\left({\hat{e}}_{j}|e_{j}\right)\right]\;\;=E\left(e_{j}\right)\;(\text{plug}\;\text{in}\;\text{formula}\;\text{(11))}\;\;=\mu_{j}.$$

We use maximum likelihood estimation [13] to estimate *μ_j_* and $\sigma_{j}^{2}$. To the best of our knowledge, this is the first time that maximum likelihood estimation is used for progress indication. We consider three cases: 1) a continuous decay method is applied to the learning rate, 2) a constant learning rate is adopted, and 3) a step decay method is applied to the learning rate. The three cases are handled in Sections III-E1 to III-E3, respectively.

#### 1) ESTIMATING *μ_j_* AND $\sigma_{j}^{2}$ WHEN A CONTINUOUS DECAY METHOD IS APPLIED TO THE LEARNING RATE

This section describes how to estimate for each *j* ≥ 1, the mean *μ_j_* and the variance $\sigma_{j}^{2}$ of the model’s generalization error at the *j*-th validation point when the learning rate changes over time based upon a continuous decay method. In such a decay method, the learning rate continuously decreases over epochs. For instance, in an exponential decay method, the learning rate adopted in the *k*-th epoch (*k* ≥ 1) is $r_{0}e^{-(k-1)\rho }$. Here, *ρ* > 0 is a constant regulating the decay rate of the learning rate. *r*_0_ > 0 is the beginning learning rate. To estimate *μ_j_* and $\sigma_{j}^{2}$, we need to estimate only four parameters: *a*, *b*, and *c* used to model *μ_j_* and *λ* used to model $\sigma_{j}^{2}$. In the following, we introduce these four parameters and then show how to estimate them.

##### a: a, b, AND c USED TO MODEL μ_j_

As in our prior work [8], we use an inverse power function [6], [16]–[19] to model the trend curve. Recall that the trend curve’s value at the *j*-th validation point is = *μ_j_*, the mean of the model’s generalization error at the *j*-th validation point.

Thus, we have

(14) $$\mu_{j}=ax_{j}^{-b}+c,$$

where *a* is >0, *b* is >0, *c* is >0, *j* is the validation point’s sequence number, and *x_j_* is the normalized number of batches of model construction finished before the *j*-th validation point

$$\overset{\text{def}}{=}\text{the}\;\text{count}\;\text{of}\;\text{batches}\;\text{of}\;\text{model}\;\text{construction}\;\;\text{finished}\;\text{before}\;\text{the}\;j\text{-th}\;\text{validation}\;\text{point}\;\text{/}\;\text{the}\;\;\text{count}\;\text{of}\;\text{batches}\;\text{of}\;\text{model}\;\text{construction}\;\text{between}\;\text{two}\;\;\text{successive}\;\text{original}\;\text{validation}\;\text{points}.$$

To estimate *μ_j_*, we need to estimate only *a*, *b*, and *c*.

##### b: λ USED TO MODEL $\sigma_{j}^{2}$

The variance of the model’s generalization error varies with the learning rate. The learning rate regulates how much the weights of the neural network and therefore the model’s generalization error change over time as well as due to random variations. The larger the learning rate, the larger the changes are likely to be. When the learning rate is 0, neither the weights of the neural network nor the model’s generalization error would ever differ from their initial values. In this case, the variance of the model’s generalization error is 0. Based upon this insight, we deem the standard deviation and the variance of the model’s generalization error to be approximately ∝ the learning rate and its square, respectively. Let *λ* > 0 denote the ratio of the variance of the model’s generalization error to the square of the learning rate. Let *r_j_* denote the learning rate right before the *j*-th validation point. The variance of the model’s generalization error at the *j*-th validation point is modelled by

(15) $$\sigma_{j}^{2}=\lambda r_{j}^{2}.$$

For each *j* ≥ 1, *r_j_* is known. To estimate $\sigma_{j}^{2}$, we need to estimate only *λ*.

In our prior work [8], the same validation set was used at each validation point. We regarded the variance of the validation error to depend only on and be approximately ∝ the square of the learning rate. In this work, the count of data instances that are in the actual validation set used at the validation point varies by validation points. Formula (13) shows that the variance of the validation error depends on the count of data instances that are in the actual validation set used at the validation point. Thus, we can no longer regard the variance of the validation error to depend only on the square of the learning rate. Rather, we regard the variance of the model’s generalization error to depend only on and be approximately ∝ the square of the learning rate.

##### c: OVERVIEW OF ESTIMATING THE PARAMETERS a, b, c, AND λ

We use maximum likelihood estimation [13] to estimate the parameters *a*, *b*, *c*, and *λ*. The likelihood function is the product of multiple integrals, which are difficult to be used directly for numerical optimization. To overcome this hurdle, for each integral, we use the probability density function of a normal distribution to approximate a key component of the integrand. In this way, we acquire a simplified form of the likelihood function, which is easy to use for numerical optimization.

In the following, we show how to estimate the parameters *a*, *b*, *c*, and *λ* in six steps. First, we present the likelihood function as the product of multiple probabilities. Second, we express each probability as an integral. Third, we show how to approximate a key component of the integrand of the integral. Fourth, we give a simplified expression of the probability. Fifth, we describe the constrained numerical optimization problem for maximizing the likelihood function and estimating *a*, *b*, *c*, and *λ*. Finally, we discuss the software package and its setting used to do numerical optimization.

##### d: THE LIKELIHOOD FUNCTION

We employ the validation curve up to the present validation point to estimate the parameters *a*, *b*, *c*, and *λ*. These parameters are then adopted to estimate the trend curve and the variance of the random noise for future validation points based upon formulas (13), (14), and (15). As an intuition, the validation points long before the present validation point may not well manifest the validation curve’s trend for future validation points and could be unsuited for estimating *a*, *b*, *c*, and *λ*. Like our prior work [8], to estimate *a*, *b*, *c*, and *λ*, we employ the last

$$w=\min\left(n,\;w^{\prime }\right)$$

validation points rather than all the validation points that we have reached so far. Here, *n* denotes the present validation point’s sequence number. *W*′ is a pre-chosen window size with a default value of 50.

Recall that ${\hat{e}}_{j}$ denotes the validation error of the model at the *j*-th validation point. We deem the validation curve to be the sum of some zero.mean random noise and a smooth trend curve. The trend curve’s value at the *j*-th validation point is = *μ_j_*. Let *ε_j_* denote the random noise at the *j*-th validation point. We have

$${\hat{e}}_{j}=\mu_{j}+\varepsilon_{j}.$$

We regard the random noises at distinct validation points to be independent of each other. Formula (14) shows that *μ_j_* is a function of *a*, *b*, and *c*. The likelihood function that we want to maximize and covers the validation errors at the last *w* validation points is

(16) $$L\left(a,b,c,\lambda |{\hat{e}}_{n-w+1},{\hat{e}}_{n-w+2},\ldots ,{\hat{e}}_{n}\right)\;=P\left({\hat{e}}_{n-w+1},{\hat{e}}_{n-w+2},\ldots ,{\hat{e}}_{n};a,b,c,\lambda \right)\;=P(\mu_{n-w+1}+\varepsilon_{n-w+1},\mu_{n-w+2}+\varepsilon_{n-w+2},\ldots ,\mu_{n}\;+\varepsilon_{n};a,b,c,\lambda )\;=P\left(\varepsilon_{n-w+1},\varepsilon_{n-w+2},\ldots ,\varepsilon_{n};a,b,c,\lambda \right)\;=\prod_{j=n-w+1}^{n}P\left(\varepsilon_{j};a,b,c,\lambda \right)\;=\prod_{j=n-w+1}^{n}P\left(\mu_{j}+\varepsilon_{j};a,b,c,\lambda \right)\;=\prod_{j=n-w+1}^{n}P\left({\hat{e}}_{j};a,b,c,\lambda \right).$$

##### e: EXPRESSING $P\left({\hat{e}}_{j};\;a,\;b,\;c,\;\lambda \right)$ AS AN INTEGRAL

Recall that ${\hat{e}}_{j}$ and *e_j_* (0 ≤ *e_j_* ≤ 1) are the validation error and the model’s generalization error at the *j*-th validation point, respectively. Using the law of total probability and Bayes’ theorem [13], we have

(17) $$P\left({\hat{e}}_{j};\;a,\;b,\;c,\;\lambda \right)\;=\int_{0}^{1}P\left({\hat{e}}_{j},e_{j};\;a,\;b,c,\lambda \right)de_{j}\;=\int_{0}^{1}P\left({\hat{e}}_{j}|e_{j};\;a,\;b,\;c,\;\lambda \right)P\left(e_{j};\;a,\;b,\;c,\;\lambda \right)de_{j}.$$

Recall that *μ_j_* and $\sigma_{j}^{2}$ are the mean and the variance of the model’s generalization error at the *j*-th validation point, respectively. Formula (14) shows that *μ_j_* is a function of *a*, *b*, and *c*. Formula (15) shows that $\sigma_{j}^{2}$ is a function of *λ*. We regard *e_j_* to follow a normal distribution with mean *μ_j_* and variance $\sigma_{j}^{2}$. That is,

(18) $$P\left(e_{j};\;a,\;b,\;c,\;\lambda \right)=P\left(e_{j};\;\mu_{j},\;\sigma_{j}^{2}\right)\;\;=\frac{1}{\sqrt{2\pi \sigma_{j}^{2}}}exp\left(-\frac{{\left(e_{j}-\mu_{j}\right)}^{2}}{2\sigma_{j}^{2}}\right).$$

Recall that *c_j_* is the count of validation instances that are misclassified by the model and in the actual validation set used at the *j*-th validation point. *V_j_* is the count of data instances that are in the actual validation set used at the *j*-th validation point. We have

$$\begin{array}{l} P\left({\hat{e}}_{j}|e_{j};\;a,\;b,\;c,\;\lambda \right)\\ \;=P\left(c_{j}/V_{j}|e_{j};\;a,\;b,\;c,\;\lambda \right)\;(\text{plug}\;\text{in}\;\text{formula}(9))\\ \;=P\left(c_{j}|e_{j}\right)\\ \;=\left(\begin{array}{c} V_{j}\\ c_{j} \end{array}\right)e_{j}^{c_{j}}{\left(1-e_{j}\right)}^{V_{j}-c_{j}}\;(\text{plug}\;\text{in}\;\text{formula}(10))\\ \;=\left(\begin{array}{c} V_{j}\\ V_{j}{\hat{e}}_{j} \end{array}\right)e_{j}^{V_{j}{\hat{e}}_{j}}{\left(1-e_{j}\right)}^{V_{j}\left(1-{\hat{e}}_{j}\right)}\\ \;\;(c_{j}=V_{j}{\hat{e}}_{j}\;\text{based}\;\text{upon}\;\text{formula}\;\text{(9))}. \end{array}$$

When maximizing the likelihood function, we can ignore the positive constant $\left(\begin{array}{c} V_{j}\\ V_{j}{\hat{e}}_{j} \end{array}\right)$ and focus on

(19) $$P\left({\hat{e}}_{j}|e_{j};\;a,\;b,\;c,\;\lambda \right)\propto e_{j}^{V_{j}{\hat{e}}_{j}}{\left(1-e_{j}\right)}^{V_{j}\left(1-{\hat{e}}_{j}\right)}.$$

Plugging formulas (18) and (19) into formula (17), we get

(20) $$P\left({\hat{e}}_{j};\;a,\;b,\;c,\;\lambda \right)\;\;\propto \int_{0}^{1}e_{j}^{V_{j}{\hat{e}}_{j}}{\left(1-e_{j}\right)}^{V_{j}\left(1-{\hat{e}}_{j}\right)}\frac{1}{\sqrt{2\pi \sigma_{j}^{2}}}\;\;\times exp\left(-\frac{{\left(e_{j}-\mu_{j}\right)}^{2}}{2\sigma_{j}^{2}}\right)de_{j}.$$

##### f: APPROXIMATING $e_{j}^{V_{j}{\hat{e}}_{j}}{\left(1-e_{j}\right)}^{V_{j}\left(1-{\hat{e}}_{j}\right)}$

Formula (16) shows that the likelihood function is the product of multiple integrals of the form given in formula (20). This form is difficult to be used directly for numerical optimization. To overcome the hurdle, for each integral, we use the probability density function of a normal distribution to approximate

$$e_{j}^{V_{j}{\hat{e}}_{j}}{\left(1-e_{j}\right)}^{V_{j}\left(1-{\hat{e}}_{j}\right)},$$

a key component of the integrand. This enables us to obtain a simplified form of the integral, which is easy to use for numerical optimization.

Recall that *V_j_* is the count of data instances that are in the actual validation set used at the *j*-th validation point. ${\hat{e}}_{j}$ and *e_j_* (0 ≤ *e_j_* ≤ 1) are the validation error and the model’s generalization error at the *j*-th validation point, respectively. When we reach the *j*-th validation point, both *V_j_* and ${\hat{e}}_{j}$ are known.

$$e_{j}^{V_{j}{\hat{e}}_{j}}{\left(1-e_{j}\right)}^{V_{j}\left(1-{\hat{e}}_{j}\right)}$$

is ∝ a beta distribution’s probability density function [13]

$$x^{\alpha -1}{\left(1-x\right)}^{\beta -1}/\text{B}(\alpha ,\;\beta ),$$

where *x* = *e_j_* (0 ≤ *x* ≤ 1) is the variable,

$$\alpha =V_{j}{\hat{e}}_{j}+1,\;\beta =V_{j}\left(1-{\hat{e}}_{j}\right)+1,$$

and B(*α*, *β*) is a normalization constant. The mean and the variance of the beta distribution are

(21) $$\mu_{j}^{\prime }=\alpha /(\alpha +\beta )\;\;=\left(V_{j}{\hat{e}}_{j}+1\right)/\left(V_{j}+2\right)$$

and

(22) $$\sigma_{j}^{\prime 2}=\alpha \beta /\left[{\left(\alpha +\beta \right)}^{2}(\alpha +\beta +1)\right]\;\;=\left(V_{j}{\hat{e}}_{j}+1\right)\left[V_{j}\left(1-{\hat{e}}_{j}\right)+1\right]/\left[{\left(V_{j}+2\right)}^{2}\left(V_{j}+3\right)\right],$$

respectively.

When *α* is ≥10 and *β* is ≥10, we can approximate the beta distribution by a normal distribution that has the same mean and variance as the beta distribution [22]. That is, we roughly have

(23) $$e_{j}^{V_{j}{\hat{e}}_{j}}{\left(1-e_{j}\right)}^{V_{j}\left(1-{\hat{e}}_{j}\right)}\propto \frac{1}{\sqrt{\sigma_{j}^{\prime 2}}}exp\left(-\frac{{\left(e_{j}-\mu_{j}^{\prime }\right)}^{2}}{2\sigma_{j}^{\prime 2}}\right).$$

Usually, *V_j_* is large enough to make *α* ≥ 10 and *β* ≥ 10. For example, even if ${\hat{e}}_{j}$ is as small as 0.02, having *V_j_* ≥ 450 is sufficient to make *α* ≥ 10 and *β* ≥ 10. Occasionally for an *j*, which typically links to an added validation point, *V_j_* may not be large enough to make *α* ≥ 10 and *β* ≥ 10. In this case, we employ the approach described in Section III.F to increase *V_j_* and make *α* ≥ 10 and *β* ≥ 10 if possible. Regardless of whether *α* is ≥10 and *β* is ≥10, we always use formula (23) to simplify the expression of $P\left({\hat{e}}_{j};\;a,\;b,\;c,\;\lambda \right)$.

##### g: COMPUTING A SIMPLIFIED EXPRESSION OF $P\left({\hat{e}}_{j};\;a,\;b,\;c,\;\lambda \right)$

Plugging formula (23) into formula (20), the integrand in formula (20) is roughly

(24) $$\propto \frac{1}{\sqrt{\sigma_{j}^{\prime 2}}}exp\left(-\frac{{\left(e_{j}-\mu_{j}^{\prime }\right)}^{2}}{2\sigma_{j}^{\prime 2}}\right)\frac{1}{\sqrt{2\pi \sigma_{j}^{2}}}exp\left(-\frac{{\left(e_{j}-\mu_{j}\right)}^{2}}{2\sigma_{j}^{2}}\right)\;=\frac{1}{\sqrt{\sigma_{j}^{2}+\sigma_{j}^{\prime 2}}}exp\left(-\frac{{\left(\mu_{j}^{\prime }-\mu_{j}\right)}^{2}}{2\left(\sigma_{j}^{2}+\sigma_{j}^{\prime 2}\right)}\right)\;\;\times \left[\frac{1}{\sqrt{2\pi {\tilde{\sigma }}_{j}^{2}}}exp\left(-\frac{{\left(e_{j}-{\tilde{\mu }}_{j}\right)}^{2}}{2{\tilde{\sigma }}_{j}^{2}}\right)\right],$$

where

(25) $${\tilde{\mu }}_{j}=\left(\sigma_{j}^{2}\mu_{j}^{\prime }+\sigma_{j}^{\prime 2}\mu_{j}\right)/\left(\sigma_{j}^{2}+\sigma_{j}^{\prime 2}\right)$$

and

(26) $${\tilde{\sigma }}_{j}^{2}=\sigma_{j}^{2}\sigma_{j}^{\prime 2}/\left(\sigma_{j}^{2}+\sigma_{j}^{\prime 2}\right).$$

In formula (24), the part in the square brackets is the probability density function of a normal distribution with mean ${\tilde{\mu }}_{j}$ and variance ${\tilde{\sigma }}_{j}^{2}$. The part outside the square brackets has nothing to do with *e_j_*. Let Φ(*x*) denote the cumulative distribution function of a standard normal distribution [13]. Plugging formula (24) into formula (20), we roughly have

(27) $$P\left({\hat{e}}_{j};\;a,\;b,\;c,\;\lambda \right)\;\propto \frac{1}{\sqrt{\sigma_{j}^{2}+\sigma_{j}^{\prime 2}}}exp\left(-\frac{{\left(\mu_{j}^{\prime }-\mu_{j}\right)}^{2}}{2\left(\sigma_{j}^{2}+\sigma_{j}^{\prime 2}\right)}\right)\int_{0}^{1}\frac{1}{\sqrt{2\pi {\tilde{\sigma }}_{j}^{2}}}\;\;\times exp\left(-\frac{{\left(e_{j}-{\tilde{\mu }}_{j}\right)}^{2}}{2{\tilde{\sigma }}_{j}^{2}}\right)de_{j}\;\;=\frac{1}{\sqrt{\sigma_{j}^{2}+\sigma_{j}^{\prime 2}}}exp\left(-\frac{{\left(\mu_{j}^{\prime }-\mu_{j}\right)}^{2}}{2\left(\sigma_{j}^{2}+\sigma_{j}^{\prime 2}\right)}\right)\;\;\times \left[\text{Φ}\left(\frac{1-{\tilde{\mu }}_{j}}{{\tilde{\sigma }}_{j}}\right)-\text{Φ}\left(\frac{-{\tilde{\mu }}_{j}}{{\tilde{\sigma }}_{j}}\right)\right].$$

##### h: MAXIMIZING THE LIKELIHOOD FUNCTION

According to formula (16), the log-likelihood function is

(28) $$\sum_{j=n-w+1}^{n}ln\;P\left({\hat{e}}_{j};\;a,\;b,\;c,\;\lambda \right).$$

Plugging formula (27) into formula (28) shows that to maximize the log-likelihood function, we only need to minimize

(29) $$\sum_{j=n-w+1}^{n}[ln\left(\sigma_{j}^{2}+\sigma_{j}^{\prime 2}\right)+\frac{{\left(\mu_{j}^{\prime }-\mu_{j}\right)}^{2}}{\sigma_{j}^{2}+\sigma_{j}^{\prime 2}}\;\;-2ln\left(\text{Φ}\left(\frac{1-{\tilde{\mu }}_{j}}{{\tilde{\sigma }}_{j}}\right)-\text{Φ}\left(\frac{-{\tilde{\mu }}_{j}}{{\tilde{\sigma }}_{j}}\right)\right)].$$

Plugging formulas (14) and (15) into formulas (25), (26), and (29), we obtain the objective function to be minimized:

(30) $$\sum_{j=n-w+1}^{n}[ln\left(\lambda r_{j}^{2}+\sigma_{j}^{\prime 2}\right)+\frac{{\left(\mu_{j}^{\prime }-ax_{j}^{-b}-c\right)}^{2}}{\lambda r_{j}^{2}+\sigma_{j}^{\prime 2}}\;\;-2ln\left(\text{Φ}\left(\frac{1-{\tilde{\mu }}_{j}}{{\tilde{\sigma }}_{j}}\right)-\text{Φ}\left(\frac{-{\tilde{\mu }}_{j}}{{\tilde{\sigma }}_{j}}\right)\right)],$$

where

(31) $${\tilde{\mu }}_{j}=\left[\lambda r_{j}^{2}\mu_{j}^{\prime }+\sigma_{j}^{\prime 2}\left(ax_{j}^{-b}+c\right)\right]/\left(\lambda r_{j}^{2}+\sigma_{j}^{\prime 2}\right)$$

and

$${\tilde{\sigma }}_{j}^{2}=\lambda r_{j}^{2}\sigma_{j}^{\prime 2}/\left(\lambda r_{j}^{2}+\sigma_{j}^{\prime 2}\right).$$

This numerical optimization problem is subject to five constraints: *a* > 0, *b* > 0, *c* > 0, *λ* > 0, and

$$ax_{n-w+1}^{-b}+c\leq 1.$$

Recall that *x_j_* denotes the normalized number of batches of model construction finished before the *j*-th validation point. To derive the last constraint, recall that *w* denotes the count of validation points used to estimate *a*, *b*, *c*, and *λ*. *n* denotes the present validation point’s sequence number. *μ_j_* (0 ≤ *μ_j_* ≤ 1) is the mean of the model’s generalization error at the *j*-th validation point. Formula (14) shows that

$$\mu_{j}=ax_{j}^{-b}+c.$$

As *j* increases, *x_j_* strictly increases and hence *μ_j_* strictly decreases. *μ_j_* is always >0. If

$$\mu_{n-w+1}=ax_{n-w+1}^{-b}+c$$

is ≤1, *μ_j_* is in [0, 1] for each *j* between *n* − *w* + 1 and *n*.

In summary, we estimate *a*, *b*, *c*, and *λ* by minimizing the objective function given by formula (30) subject to five constraints: *a* > 0, *b* > 0, *c* > 0, *λ* > 0, and

$$ax_{n-w+1}^{-b}+c\leq 1.$$

##### i: THE SOFTWARE PACKAGE AND ITS SETTING USED TO DO NUMERICAL OPTIMIZATION

We use the interior.point algorithm [23, Ch. 19], [24] implemented in the software package Artelys Knitro [25] to solve this constrained minimization problem. Typically, the estimated *a*, *b*, *c*, and *λ* are roughly on the order of magnitude of 0.1, 0.1 [17]–[19], 0.1, and 100, respectively. Accordingly, when conducting numerical optimization, we initialize *a*, *b*, *c*, and *λ* to 0.1, 0.1, 0.1, and 100, respectively.

During the constrained numerical optimization process, one could allow the constraints to be violated [23, Ch. 15.4]. However, if the constraint

$$ax_{n-w+1}^{-b}+c\leq 1$$

is violated, ${\tilde{\mu }}_{j}$ could be >1 for one or more *j* between *n* − *w* + 1 and *n* (see formula (31)). If ${\tilde{\mu }}_{j}$ is ≫1 and ${\tilde{\sigma }}_{j}$ is small, numerical underflow could occur in computing

$$\text{Φ}\left(\left(1-{\tilde{\mu }}_{j}\right)/{\tilde{\sigma }}_{j}\right)-\text{Φ}\left(-{\tilde{\mu }}_{j}/{\tilde{\sigma }}_{j}\right),$$

causing issues when we compute

$$ln\left(\text{Φ}\left(\left(1-{\tilde{\mu }}_{j}\right)/{\tilde{\sigma }}_{j}\right)-\text{Φ}\left(-{\tilde{\mu }}_{j}/{\tilde{\sigma }}_{j}\right)\right)$$

in formula (30). To avoid this issue, we set the bar_feasible parameter in Artelys Knitro to either 1 or 3 to ensure that the five constraints are always satisfied during the entire constrained numerical optimization process [26].

#### 2) ESTIMATING *μ_j_* AND $\sigma_{j}^{2}$ WHEN A CONSTANT LEARNING RATE IS ADOPTED

In this section, we describe how to estimate for each *j* ≥ 1, the mean *μ_j_* and the variance $\sigma_{j}^{2}$ of the model’s generalization error at the *j*-th validation point when a constant learning rate is used. This case is a special case of applying an exponential decay method to the learning rate, when the constant *ρ* regulating the decay rate of the learning rate is 0. We employ the same approach in Section III-E1 to estimate *μ_j_* and $\sigma_{j}^{2}$ for each *j* ≥ 1.

#### 3) ESTIMATING *μ_j_* AND $\sigma_{j}^{2}$ WHEN A STEP DECAY METHOD IS APPLIED TO THE LEARNING RATE

This section describes how to estimate for each *j* ≥ 1, the mean *μ_j_* and the variance $\sigma_{j}^{2}$ of the model’s generalization error at the *j*-th validation point when the learning rate changes over time based upon a step decay method.

**FIGURE 5.**
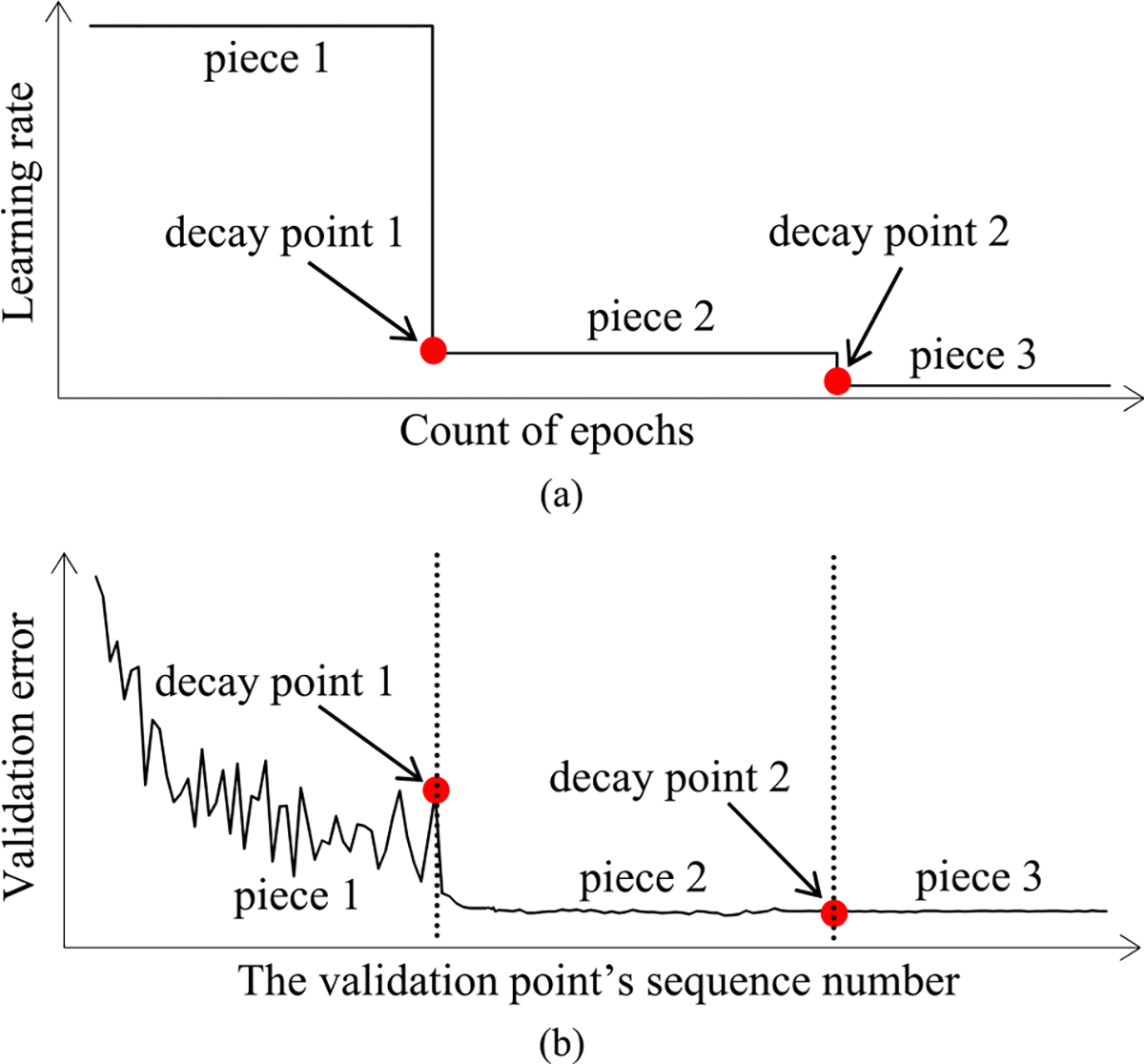
When the learning rate changes over time based upon a step decay method, the learning rate over epochs and an example validation curve. (a) The learning rate over epochs. (b) An example validation curve.

As Fig. 5(a) shows, in a step decay method, we cut the learning rate by a pre-chosen factor that is >1 after a given number of epochs. This factor could change over epochs in a pre-determined fashion. Fig. 5(b) presents a correspondent example validation curve. A decay point is defined as an original validation point at which the learning rate is cut. The decay points partition the validation curve into several pieces. For every *j* ≥ 1, the first original validation point on the (*j* + 1)-th piece is the *j*-th decay point. When model construction begins, both the learning rate used on and the position of each piece are known.

As we move from one piece of the validation curve to the next, both the learning rate and the variance of the model’s generalization error change. We consider this when estimating *μ_j_* and $\sigma_{j}^{2}$ for each *j* ≥ 1. As in Section III-E1, to estimate *μ_j_* and $\sigma_{j}^{2}$, we need to estimate only the four parameters *a*, *b*, *c*, and *λ* used to model *μ_j_* and $\sigma_{j}^{2}$. There are two possible cases: 1) the present validation point resides on the first piece of the validation curve, and 2) the present validation point resides on the *k*-th (*k* ≥ 2) piece of the validation curve. We discuss the two cases sequentially.

##### Case 1 (The Present Validation Point Resides on the First Piece of the Validation Curve)

When the present validation point resides on the first piece of the validation curve, we adopt the method in Section III-E1 to estimate *a*, *b*, *c*, and *λ*.

##### Case 2 (The Present Validation Point Resides on the k.th (k ≥ 2) Piece of the Validation Curve)

**FIGURE 6.**
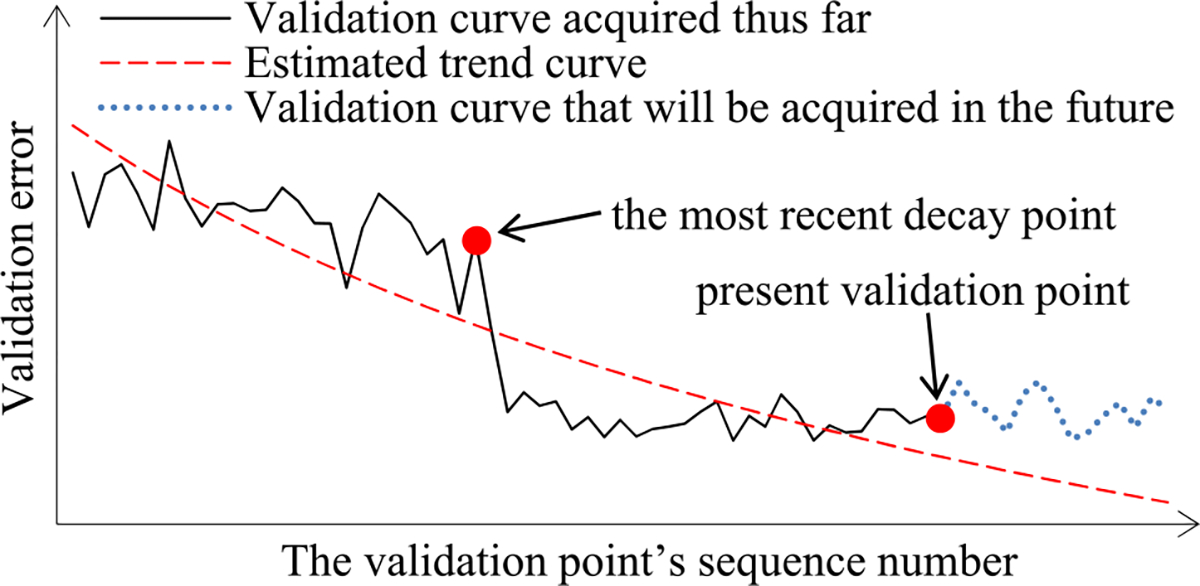
Employing the method in Section III-E1 to estimate the trend curve when one arrives at a validation point that is not far after the most recent decay point.

Next, we discuss the case of the present validation point residing on the *k*-th (*k* ≥ 2) piece of the validation curve. As shown in Fig. 5(b), because of the decay of the learning rate at a decay point, the validation curve frequently drops abruptly at this point as well as at the next few validation points. As Fig. 6 shows, when one arrives at a validation point that is not far after such a decay point, this drop could result in an inaccurately estimated trend curve if the estimation method in Section III-E1 were used.

**FIGURE 7.**
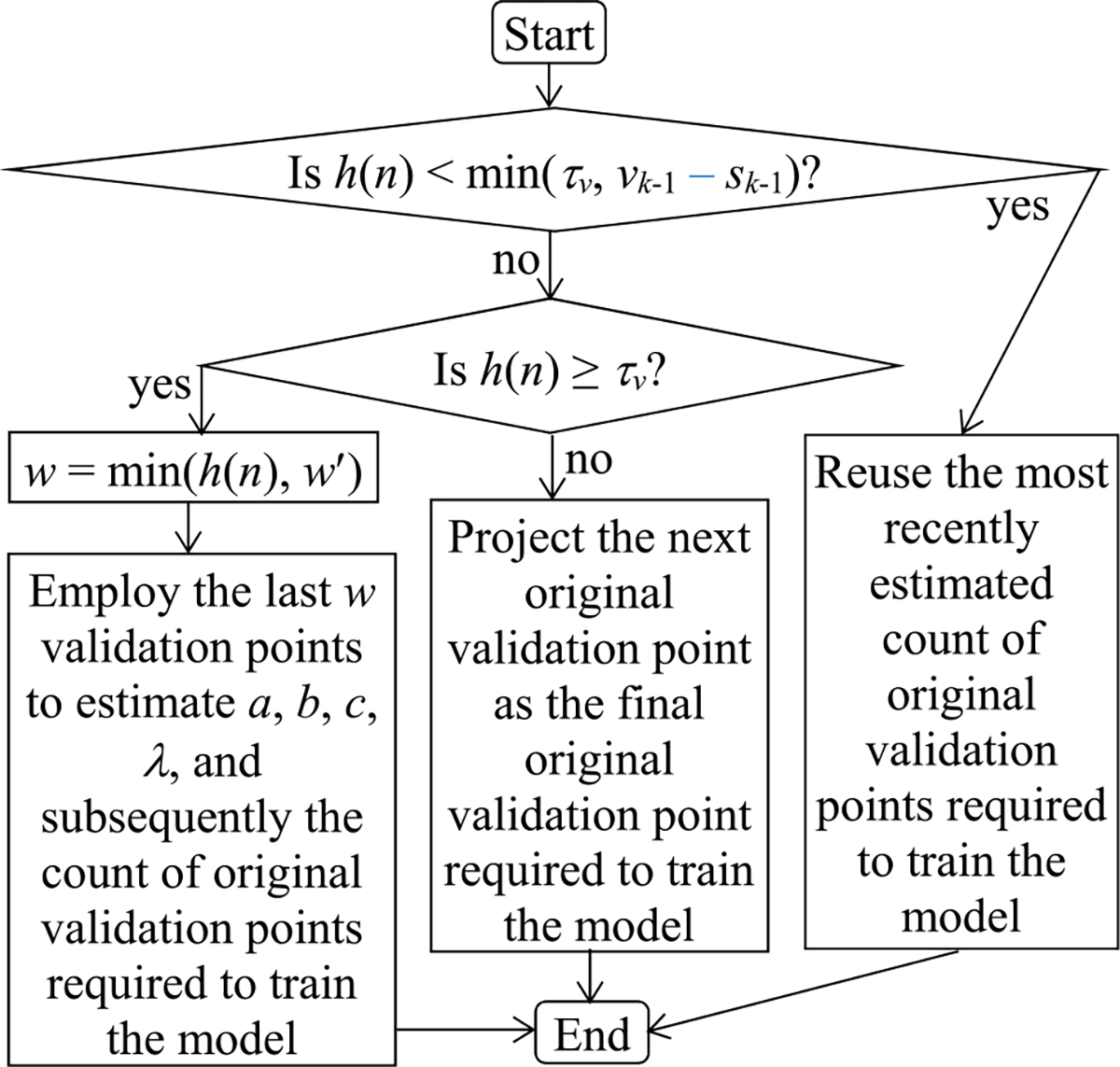
The flowchart of estimating the count of original validation points required to train the model when the present validation point resides on the *k-th* (*k* ≥ 2) piece of the validation curve.

To deal with this issue, we revise the estimation method in Section III-E1. Let *l_j_* (*j* ≥ 1) denote the count of validation points that are on the *j*-th piece of the validation curve. Each *l_j_* is known beforehand. Recall that at least *τ_v_* = 4 data points are needed to estimate *a*, *b*, *c*, and *λ*. Usually, *l_j_* is ≥ *τ_v_* for each *j* ≥ 1.

$$s_{k-1}=\sum_{j=1}^{k-1}l_{j}$$

is the sequence number of the final validation point that is on the prior piece of the validation curve. Let *v*_*k*−1_ denote the count of both original and added validation points required to train the model that is projected at the final validation point on the prior piece. If the *v*_*k*−1_-th validation point resides on the present *k*-th piece, *v*_*k*−1_ − *s*_*k*−1_ is this validation point’s sequence number on the present *k*-th piece. Recall that *n* is the present validation point’s sequence number. Let *h*(*n*) denote the present validation point’s sequence number on the present *k*-th piece. *h*(*n*) is ≤ *l_k_*. There are two possible scenarios (see Fig. 7).

In the first scenario, *h*(*n*) is <min(*τ_v_*, *v*_*k*−1_ − *s*_*k*−1_). In this case, we do not have enough validation points to estimate *a*, *b*, *c*, and *λ*. We reuse the most recently estimated count of original validation points required to train the model. Since *τ_v_* is small, we often pass the phase of not updating the estimated count of original validation points required to train the model in a reasonably short period of time.

In the second scenario, *h*(*n*) is ≥min(*τ_v_*, *v*_*k*−1_ − *s*_*k*−1_). If *v*_*k*−1_ − *s*_*k*−1_ ≤ *h*(*n*) < *τ_v_*, we project the next original validation point as the final original validation point required to train the model. Otherwise, if *h*(*n*) is ≥ *τ_v_*, we revise the method in Section III-E1 in the following two ways to estimate *a*, *b*, *c*, and *λ*.

First, recall that *x_j_* denotes the normalized number of batches of model construction finished before the *j*-th validation point. The trend curve’s value at the *j*-th validation point is = *μ_j_*. As shown in Fig. 5(b), if moved to the left by $x_{s_{k-1}}$, the present piece of the trend curve has approximately the same form as an inverse power function. We adopt the same shifted inverse power function

$$\mu_{j}=a{\left(x_{j}-x_{s_{k-1}}\right)}^{-b}+c$$

rather than formula (14) to model *μ_j_*.

Second, recall that *w*′ denotes the largest number of validation points permitted to estimate *a*, *b*, *c*, and *λ*. *n* denotes the present validation point’s sequence number. *h*(*n*) denotes the present validation point’s sequence number on the present piece of the validation curve. We employ the last

$$w=\min\left(h(n),\;w^{\prime }\right)$$

validation points on the present piece of the validation curve rather than the last min(*n*, *w*′) validation points to estimate *a*, *b*, *c*, and *λ*.

### F. DETERMINING V_min_

In this section, we show how to determine *V_min_*, the minimum number of data instances needed in the randomly sampled subset of the full validation set used at an added validation point.

Recall that *V_j_* (*j* ≥ 1) is the count of data instances that are in the actual validation set used at the *j*-th validation point. ${\hat{e}}_{j}$ denotes the validation error of the model at the *j*-th validation point. At an added validation point, *V_j_* is computed by formula (8) that involves *V_min_*. In Section III-E1, we use a normal distribution to approximate a beta distribution with parameters

$$\alpha =V_{j}{\hat{e}}_{j}+1$$

and

$$\beta =V_{j}\left(1-{\hat{e}}_{j}\right)+1.$$

This approximation is reasonably precise if *α* is ≥10 and *β* is ≥10 [22], which is equivalent to $V_{j}\geq 9/{\hat{e}}_{j}$ and $V_{j}\geq 9/(1-{\hat{e}}_{j})$. If we know ${\hat{e}}_{j}^{’}\text{s}$ lower bound *b_l_* > 0 and upper bound *b_u_* < 1, we can set *V_min_* to

$$9/\min\left(b_{l},\;1-b_{u}\right)$$

to raise the chance of *α* being ≥10 and *β* being ≥10 for each *j* ≥ 1. However, *b_l_* and *b_u_* are unknown beforehand. To address this issue, we start from an initial estimate ${\hat{b}}_{l}$ of *b_l_* and an initial estimate ${\hat{b}}_{u}$ of *b_u_* and set *V_min_* to

(32) $$9/\min\left({\hat{b}}_{l},\;1-{\hat{b}}_{u}\right).$$

During model construction, ${\hat{e}}_{j}$ could fall out of $\left[{\hat{b}}_{l},\;{\hat{b}}_{u}\right]$ at some added validation point, making it possible to have *α* < 10 or *β* < 10. At any added validation point, if ${\hat{e}}_{j}$ falls out of $\left[{\hat{b}}_{l},\;{\hat{b}}_{u}\right]$, we lower ${\hat{b}}_{l}$ or raise ${\hat{b}}_{u}$ to make $\left[{\hat{b}}_{l},\;{\hat{b}}_{u}\right]$ include ${\hat{e}}_{j}$ and then re-compute *V_min_* to make it larger. At any original validation point, if ${\hat{e}}_{j}$ falls out of $\left[{\hat{b}}_{l},\;{\hat{b}}_{u}\right]$, we do not adjust ${\hat{b}}_{l}$ and ${\hat{b}}_{u}$ because the full validation set is used and there is no way to make *V_j_* larger.

We have two considerations when setting the initial values of ${\hat{b}}_{l}$ and ${\hat{b}}_{u}$. First, the larger the ${\hat{b}}_{l}$ and the smaller the ${\hat{b}}_{u}$, the more likely ${\hat{e}}_{j}$ will fall out of $\left[{\hat{b}}_{l},\;{\hat{b}}_{u}\right]$ at some added validation point during model construction, which is undesirable. Second, if ${\hat{b}}_{l}$ is too small or ${\hat{b}}_{u}$ is too large, the *V_min_* computed by formula (32) will be too large. Consequently, *V_j_* could also be too large, undesirably increasing the progress indicator’s run-time overhead. To strike a balance between these two considerations, we set the initial values of ${\hat{b}}_{l}$ and ${\hat{b}}_{u}$ to 0.02 and 0.98, respectively.

During model construction, if the validation error ${\hat{e}}_{j}$ at an added validation point is outside of $\left[{\hat{b}}_{l},\;{\hat{b}}_{u}\right]$, we proceed as follows:

1. Step 1: If ${\hat{e}}_{j}$ is $>{\hat{b}}_{u}$, we change ${\hat{b}}_{u}$ to ${\hat{e}}_{j}$. If ${\hat{e}}_{j}$ is $<{\hat{b}}_{l}$, we change ${\hat{b}}_{l}$ to ${\hat{e}}_{j}$.
2. Step 2: Use formula (32) to re-compute *V_min_*. If ${\hat{e}}_{j}$ is = 0 or 1, which is unlikely to occur in practice, we set *V_min_* to +∞.
3. Step 3: Use formula (8) to re-compute *V*′, the uniform number of data instances that are in the randomly sampled subset of the full validation set used at each added validation point.
4. Step 4: If the new *V*′ differs from the old *V*′, we re-sample the full validation set to obtain a new subset and re-compute ${\hat{e}}_{j}$, the model’s error rate on the subset. The count of data instances that are in the subset is the new *V*′, which will also be used at each added validation point after the present validation point.
5. Step 5: If ${\hat{e}}_{j}$ is re-computed in Step 4 and the new ${\hat{e}}_{j}$ is outside of $\left[{\hat{b}}_{l},\;{\hat{b}}_{u}\right]$, we repeat Steps 1–4 until the new ${\hat{e}}_{j}$ is within $\left[{\hat{b}}_{l},\;{\hat{b}}_{u}\right]$.

In practice, we rarely need to change *V*′ from its initially computed value because 1) the initial $\left[{\hat{b}}_{l},\;{\hat{b}}_{u}\right]$ is wide and has a high likelihood to include ${\hat{e}}_{j}$, and 2) if the initially computed *V*′ is > the *V_min_* re-computed in Step 2, no value change will be made to *V*′ in Step 3.

### G. ESTIMATING THE MODEL CONSTRUCTION COST BASED UPON THE PROJECTED COUNT OF ORIGINAL VALIDATION POINTS REQUIRED TO TRAIN THE MODEL

After estimating the trend curve and the variance of the random noise, we can project the model construction cost. The Monte Carlo simulation method in our prior paper [8] is used to estimate *n_v_*, the count of original validation points required to train the model. Recall that *V*′ is the uniform number of data instances that are in the randomly sampled subset of the full validation set used at each added validation point. *n_j_* (0 ≤ *j* ≤ *v_max_* − 1) is the count of validation points to be added between the *j*-th and the (*j* + 1)-th original validation points. *q* is the constant regulating the decay rate of *n_j_* (0 ≤ *j* ≤ *v_max_* − 1) in the exponential decay schema. Our prior work [8] shows that the mean quantity of work taken to process a validation instance one time to calculate the validation error is 1/3 unit of work. The model construction cost is the sum of three components:

1. The cost to process the training instances, which is computed using formula (1).
2. The cost to calculate the validation errors at the original validation points, which is computed using formula (2).
3. The cost to calculate the validation errors at the added validation points

$$=\text{the}\;\text{total}\;\text{count}\;\text{of}\;\text{validation}\;\text{points}\;\text{added}\;\text{before}\;\;\text{the}\;n_{v}\text{-th}\;\text{original}\;\text{validation}\;\text{point}\;\times \;\text{the}\;\text{uniform}\;\;\text{number}\;\text{of}\;\text{data}\;\text{instances}\;\text{that}\;\text{are}\;\text{in}\;\text{the}\;\text{randomly}\;\;\text{sampled}\;\text{subset}\;\text{of}\;\text{the}\;\text{full}\;\text{validation}\;\text{set}\;\text{used}\;\text{at}\;\;\text{each}\;\text{added}\;\text{validation}\;\text{point}\;\;\times \;\text{the}\;\text{mean}\;\text{quantity}\;\text{of}\;\text{work}\;\text{taken}\;\text{to}\;\;\text{process}\;\text{a}\;\text{validation}\;\text{instance}\;\text{one}\;\text{time}\;\;\text{to}\;\text{calculate}\;\text{the}\;\text{validation}\;\text{error}\;=\sum_{j=0}^{n_{v}}n_{j}\times V^{\prime }/3\;=\sum_{j=0}^{n_{v}}\left\lfloor n_{0}q^{j}\right\rceil \times V^{\prime }/3.$$

## IV. PERFORMANCE

This section presents the performance test results of our new progress indication method for constructing deep learning models. TensorFlow is a commonly used open-source software package for deep learning created by Google [14]. We implemented our new method in TensorFlow Version 1.13.1. In each test, our progress indicators gave informative estimates and revised them every 10 seconds with minute overhead, fulfilling the progress indication goals of low overhead, continuously revised updates, and reasonable pacing listed in our prior paper [6].

### A. DESCRIPTION OF THE EXPERIMENTS

The experiments were performed by running TensorFlow on a Digital Storm workstation. The workstation runs the Ubuntu 18.04.02 operating system and has 64GB memory, one eight-core Intel Core i7–9800X 3.8GHz CPU, one GeForce RTX 2080 Ti GPU, one 3TB SATA disk, and one 500GB solid-state drive. Every deep learning model was constructed on an unloaded system and using the GPU.

We tested two standard deep learning models: the Gated Recurrent Unit (GRU) model, a recurrent neural network, used in Purushotham *et al.* [27] and the convolutional neural network GoogLeNet [28]. For every model, we tested four standard optimization algorithms for constructing deep learning models: root mean square propagation (RMSprop) [29], classical stochastic gradient descent (SGD) [30], adaptive gradient (AdaGrad) [31], and adaptive moment estimation (Adam) [32]. For each (deep learning model, optimization algorithm) pair, three learning rate decay methods were tested: using an exponential decay method, a step decay method, and a constant learning rate. We present the test results for GoogLeNet using Adam and the GRU model using RMSprop. The test results for the other (deep learning model, optimization algorithm) pairs are similar and shown in the Appendix in the full version of the paper [33]. There is one exception. For the step decay method, we present the test results for GoogLeNet using Adam. The test results for using RMSprop and the step decay method to construct the GRU model are similar and shown in the Appendix in the full version of the paper [33].

We employed two popular benchmark data sets shown in Table 3: CIFAR-10 [34] and MIMIC-III [35]. GoogLeNet was trained on CIFAR-10. In CIFAR-10, every data instance is an image of size 32 × 32. CIFAR-10 was split into a validation set and a training set as described in Krizhevsky [34]. The GRU model was trained on a subset of the MIMIC-III data set called “Feature Set C, 48-h data” to perform the “ICD-9 code group prediction” task in Purushotham *et al.* [27]. In the subset, every data instance is a sequence of length 48. The subset was partitioned into a validation set and a training set as described in Purushotham *et al.* [27].

Except for the largest number of epochs permitted to train the model and the learning rate decay method, all the hyper-parameters were given their default values that appeared in the open source code of GoogLeNet and the GRU model [36], [37]. In particular, the count of training instances in every batch was = 100 and 128 for the GRU model and GoogLeNet, respectively. In each test, the beginning learning rate was = 0.001. The patience *p* was = 11, an integer randomly selected from [3, 25]. The min_delta *δ* was = 0.00131, a number randomly selected from [0, 0.01]. The largest number of epochs permitted to train the model was = 150. An original validation point was put at or near the end of each epoch of model construction. Accordingly, the count of batches of model construction between two successive original validation points was 390 and 205 for GoogLeNet and the GRU model, respectively.

Recall that *v_max_* denotes the largest number of original validation points permitted to construct the model. *n_j_* (0 ≤ *j* ≤ *v_max_* − 1) is the count of validation points added between the *j*-th and the (*j* + 1)-th original validation points. *n*_0_ is the count of validation points added before the first original validation point. *q* is the constant regulating the decay rate of *n_j_* (0 ≤ *j* ≤ *v_max_* − 1) in the exponential decay schema. *V*′ is the uniform number of data instances that are in the randomly sampled subset of the full validation set used at each added validation point. For each of GoogLeNet and the GRU model, Table 4 shows the *n*_0_, *q*, and *V*′ set by the approach given in Section III-B. In our experiments, *V*′ never changed during model construction.

### B. ACCURACY MEASURE

**FIGURE 8.**
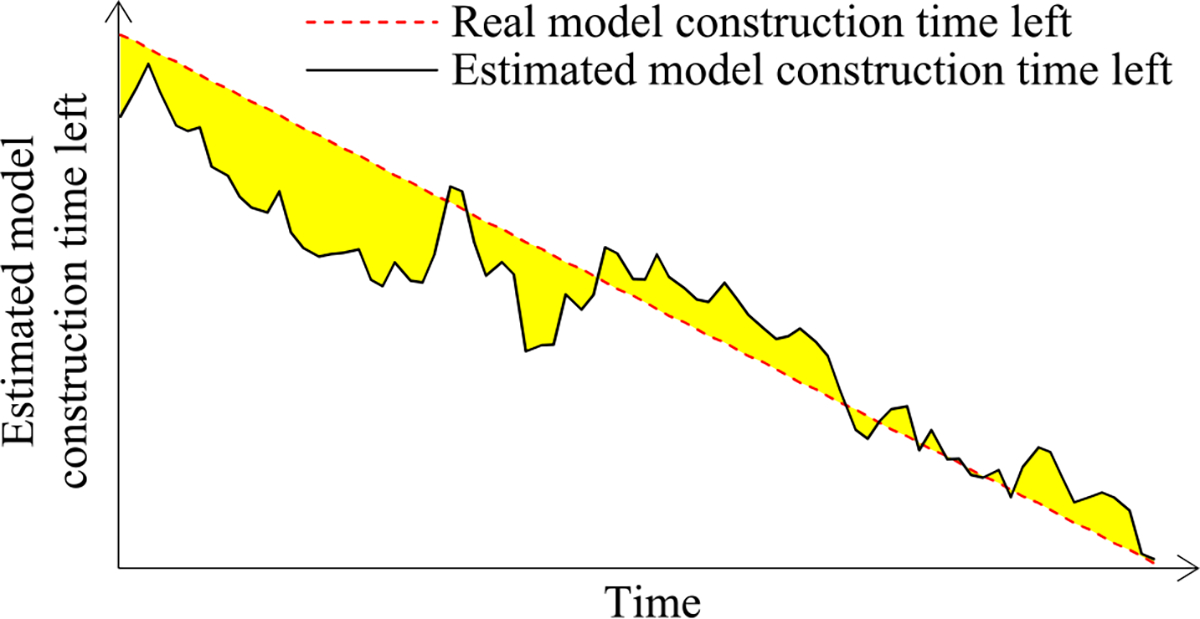
The areas of the regions employed to calculate the average prediction error.

We used the average prediction error adopted in Chaudhuri *et al.* [38] to gauge the progress indicator’s estimation accuracy. The average prediction error is the ratio of a numerator to a denominator (see Fig. 8). The area of the region between a straight diagonal line and a curve is the numerator. The straight line shows the real model construction time left. The curve shows the progress indicator’s estimate of the model construction time left over time. The area of the triangle created by the straight diagonal line, the y-axis, and the x-axis is the denominator. The larger the average prediction error, the less accurate the estimates given by the progress indicator.

### C. COMPARISON OF THREE PROGRESS INDICATION METHODS FOR CONSTRUCTING DEEP LEARNING MODELS

We compared the accuracy of the progress estimates provided by three progress indication methods for constructing deep learning models:

1. **Method 1**: This is our prior method [8].
2. **Method 2**: This is a hybrid of our prior and new methods. We use the approach in Section III-B to insert extra validation points between the original validation points, the approach in Section III-C to set the uniform number of data instances that are in the randomly sampled subset of the full validation set used at each added validation point, the approach in our prior paper [8] to predict the count of original validation points required to train the model, and the approach in Section III-G to estimate the model construction cost based upon the projected number. We disregard the dependency of the random noise’s variance on the size of the actual validation set used at the validation point. Instead, as in our prior paper [8], we deem the random noise’s variance to be approximately α the square of the learning rate with no reliance on the size of the actual validation set used at the validation point.
3. **Method 3**: This is our new method shown in Section III.

We conducted 24 tests, one for every combination of a deep learning model, an optimization algorithm, and a learning rate decay method. In each test, we constructed the deep learning model five times, each in a distinct run. In each run, we used each of the three progress indication methods to provide progress estimates. For each test, Table 5 shows the standard deviation and the mean of the average prediction error over the five runs for each of the three methods. For each test, the smallest mean of the average prediction error over the five runs across the three methods is marked in bold in Table 5.

#### 1) COMPARISON OF METHODS 1 AND 3

In 20 of the 24 tests, method 3 beat method 1 and had a smaller mean of the average prediction error over the five runs. Method 1 outperformed method 3 in the other two tests: 1) using Adam and a constant learning rate to construct GoogLeNet, and 2) using AdaGrad and applying a step decay method to the learning rate to construct GoogLeNet. The mean of the average prediction error over all runs in all tests for method 3 is 0.48, which is 57.5% lower than the corresponding mean of 1.13 for method 1. Thus, compared with using our prior method [8], using our new method reduces the progress indicator’s prediction error of the model construction time left. Moreover, our new method gave decently accurate estimates of the model construction time left.

#### 2) COMPARISON OF METHODS 2 AND 3

In 18 of the 24 tests, method 3 beat method 2 and had a smaller mean of the average prediction error over the five runs. Method 2 outperformed method 3 in the other five tests: 1) using Adam and a constant learning rate to construct GoogLeNet, 2) using RMSprop and applying a step decay method to the learning rate to construct GoogLeNet, 3) using AdaGrad and applying a step decay method to the learning rate to construct GoogLeNet, 4) using SGD and a constant learning rate to construct the GRU model, and 5) using SGD and applying a step decay method to the learning rate to construct the GRU model. The mean of the average prediction error over all runs in all tests for method 3 is 0.48, which is 20.0% lower than the corresponding mean of 0.60 for method 2. Thus, considering the dependency of the random noise’s variance on the size of the actual validation set used at the validation point raises the progress indicator’s prediction accuracy.

In Sections IV-D to IV-F and the Appendix in the full version of the paper [33], we focus on the new progress indication method described in Section III. Yet, for the model construction time left, we show the estimates provided by both the old and the new progress indication methods. Recall that in each of the 24 tests, we constructed the deep learning model five times, each in a distinct run. We randomly selected one of the five runs and present the outputs of the progress indicator over time for that run.

### D. TEST RESULTS FOR ADOPTING A CONSTANT LEARNING RATE

This section presents the test results for adopting a constant learning rate.

#### 1) TEST RESULTS FOR CONSTRUCTING GOOGLENET

**FIGURE 9.**
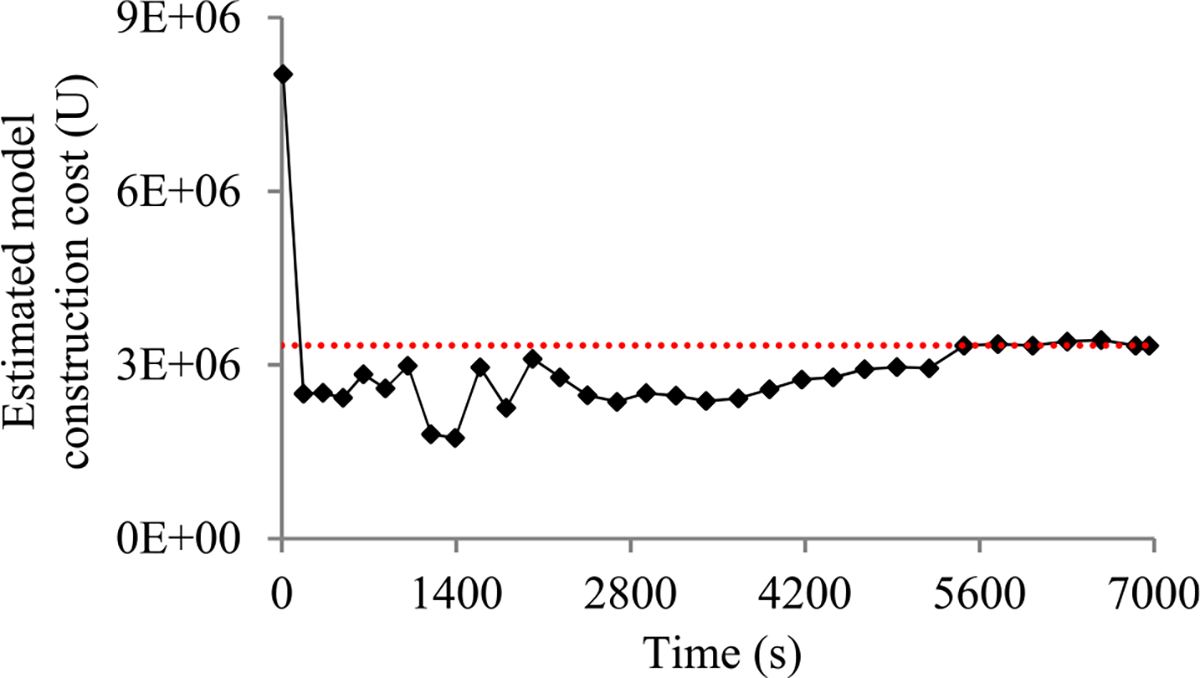
Model construction cost estimated over time (using Adam and a constant learning rate to construct GoogLeNet).

In the test, we used the Adam optimization algorithm and a constant learning rate to construct GoogLeNet. Fig. 9 depicts the progress indicator’s estimated model construction cost over time, with the dotted horizontal line showing the real model construction cost. Before reaching *τ_v_* = 4 validation points within 39 seconds, the progress indicator estimated the model construction cost based upon the largest number of original validation points permitted to train the model, which diverged notably from the real count of original validation points required to train the model. As a result, the estimated model construction cost greatly differed from the real model construction cost. After reaching four or more validation points, the progress indicator refined the estimated model construction cost for it to become more accurate over time.

**FIGURE 10.**
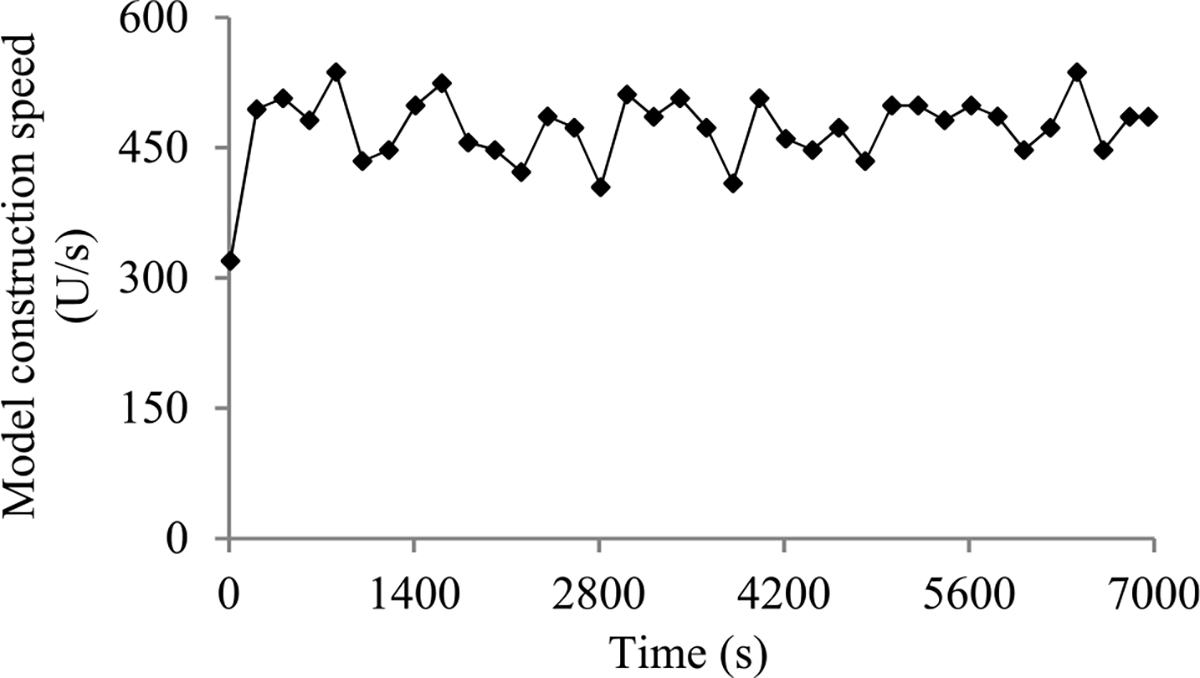
Model construction speed over time (using Adam and a constant learning rate to construct GoogLeNet).

Fig. 10 depicts the model construction speed that the progress indicator observed over time. This speed was relatively stable during the whole model construction process.

**FIGURE 11.**
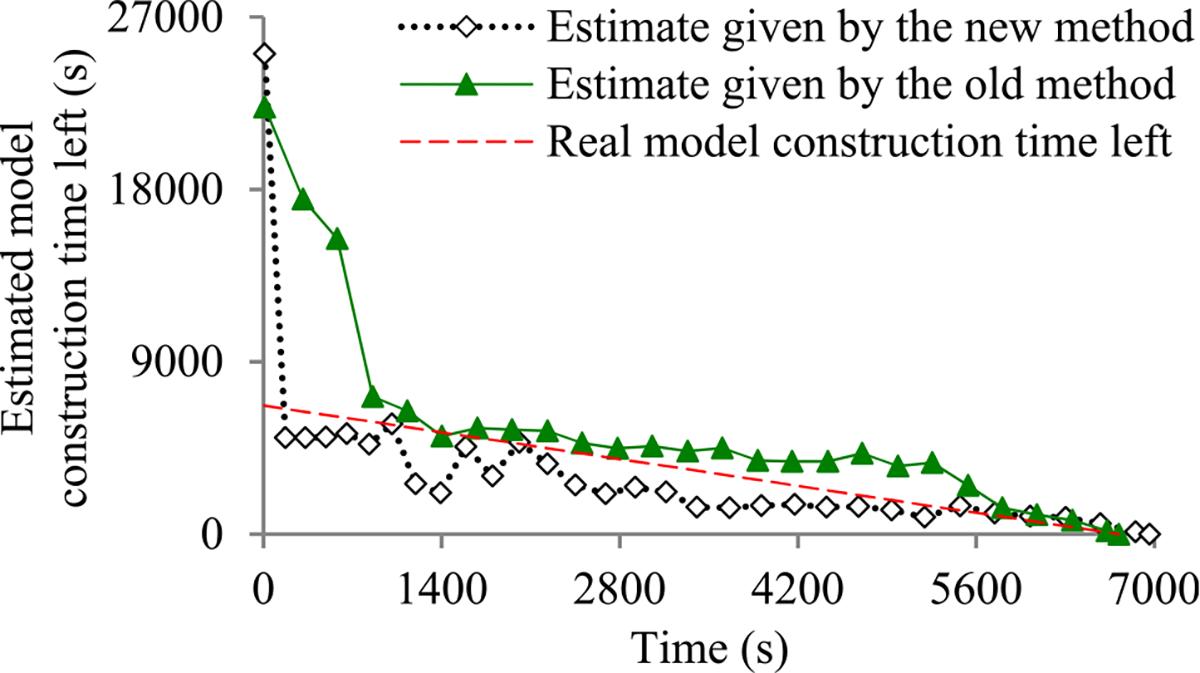
Estimated model construction time left (using Adam and a constant learning rate to construct GoogLeNet).

**FIGURE 12.**
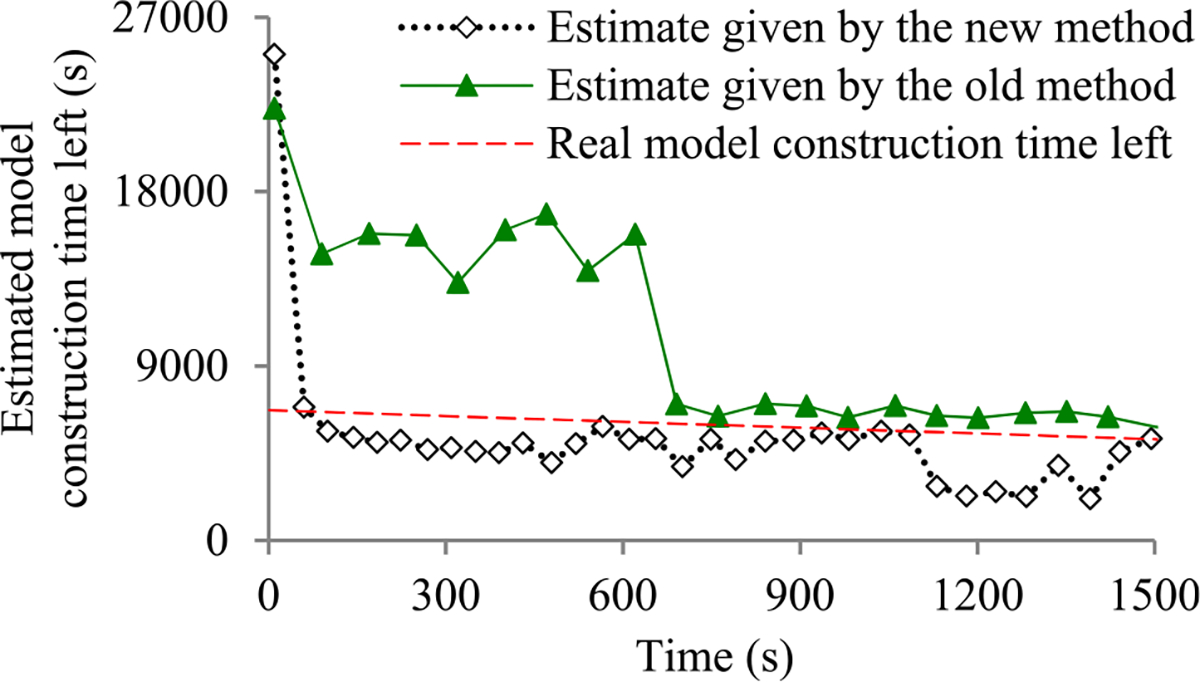
Estimate of the model construction time left at the early stage of model construction (using Adam and a constant learning rate to construct GoogLeNet).

Fig. 11 and 12 depict the remaining model construction time estimated by the old and the new progress indication methods over time, with the dashed line showing the real model construction time left. Before 691 seconds, the old method’s [8] estimate of the model construction time left differed notably from the real model construction time left. The new method reached the stage of giving relatively accurate estimates of the model construction time left much faster than the old method.

**FIGURE 13.**
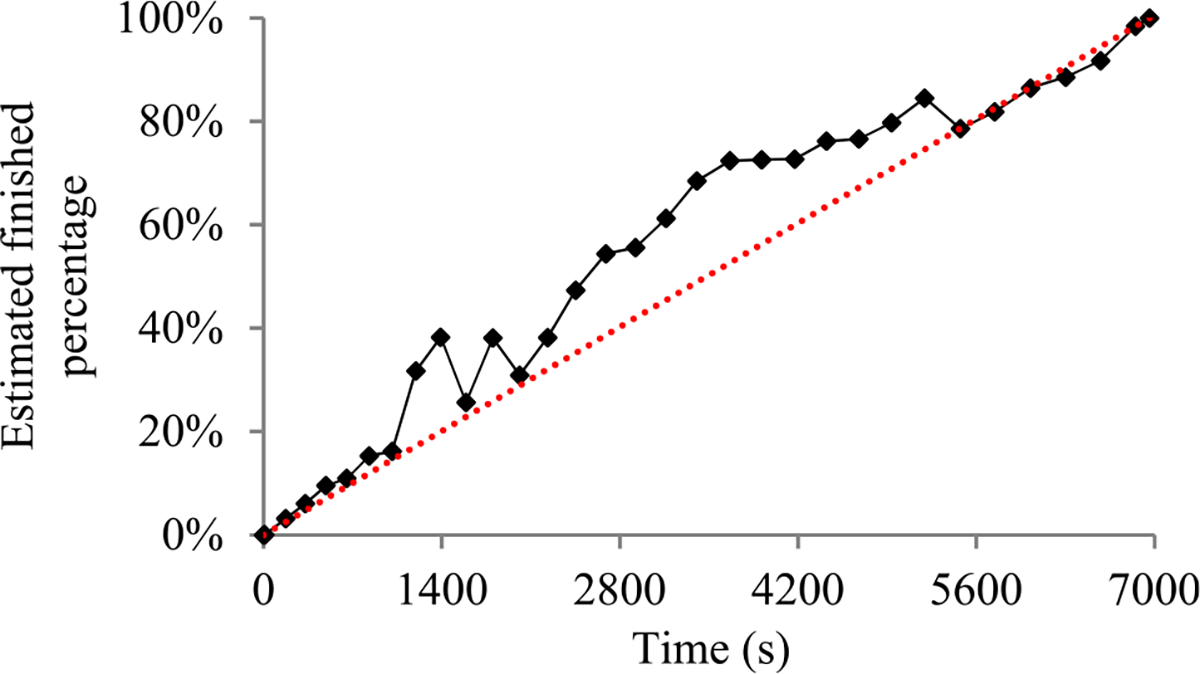
Finished percentage estimated over time (using Adam and a constant learning rate to construct GoogLeNet).

Fig. 13 depicts the progress indicator’s estimate over time of the finished percentage of model construction work. The curve showing the estimated finished percentage is reasonably close to the diagonal dotted line linking the upper right and the lower left corners.

#### 2) TEST RESULTS FOR CONSTRUCTING THE GRU MODEL

In the test, we used the RMSprop optimization algorithm and a constant learning rate to construct the GRU model. We wanted to show that the estimates given by the progress indicator can be decently accurate for distinct kinds of neural networks.

**FIGURE 14.**
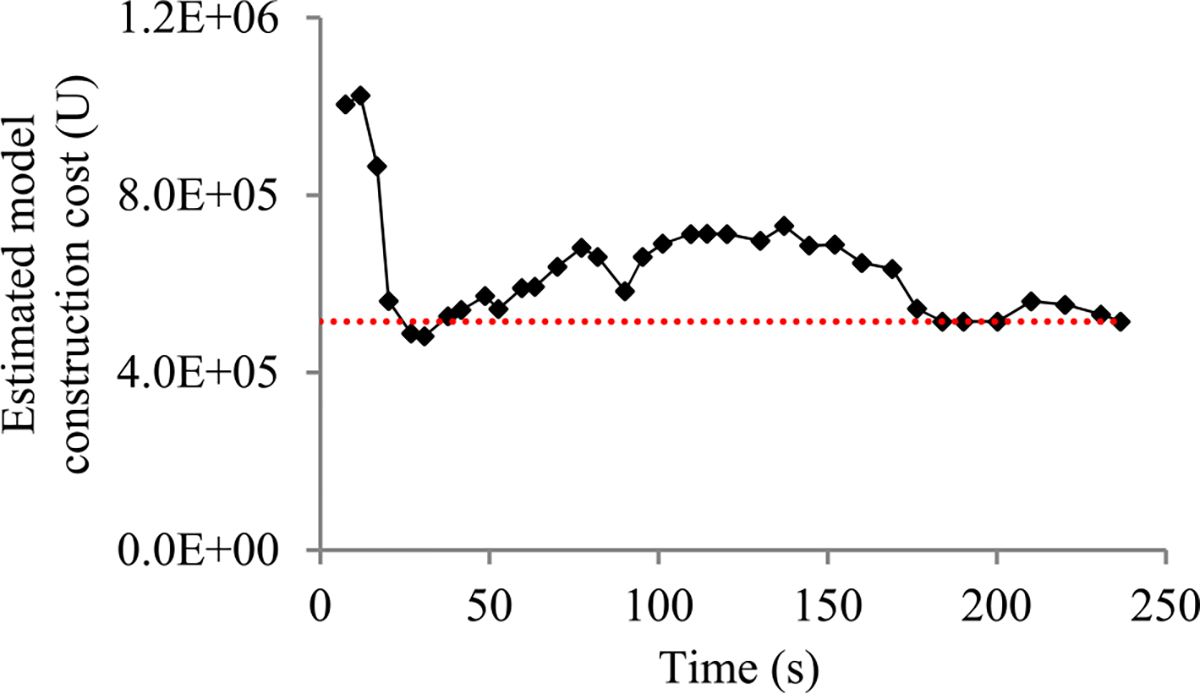
Model construction cost estimated over time (using RMSprop and a constant learning rate to construct the GRU model).

Fig. 14 depicts the progress indicator’s estimated model construction cost over time, with the dotted horizontal line showing the real model construction cost. After we reached *τ_v_* = 4 validation points within 7 seconds, the estimated model construction cost became decently accurate for the rest of the model construction process.

**FIGURE 15.**
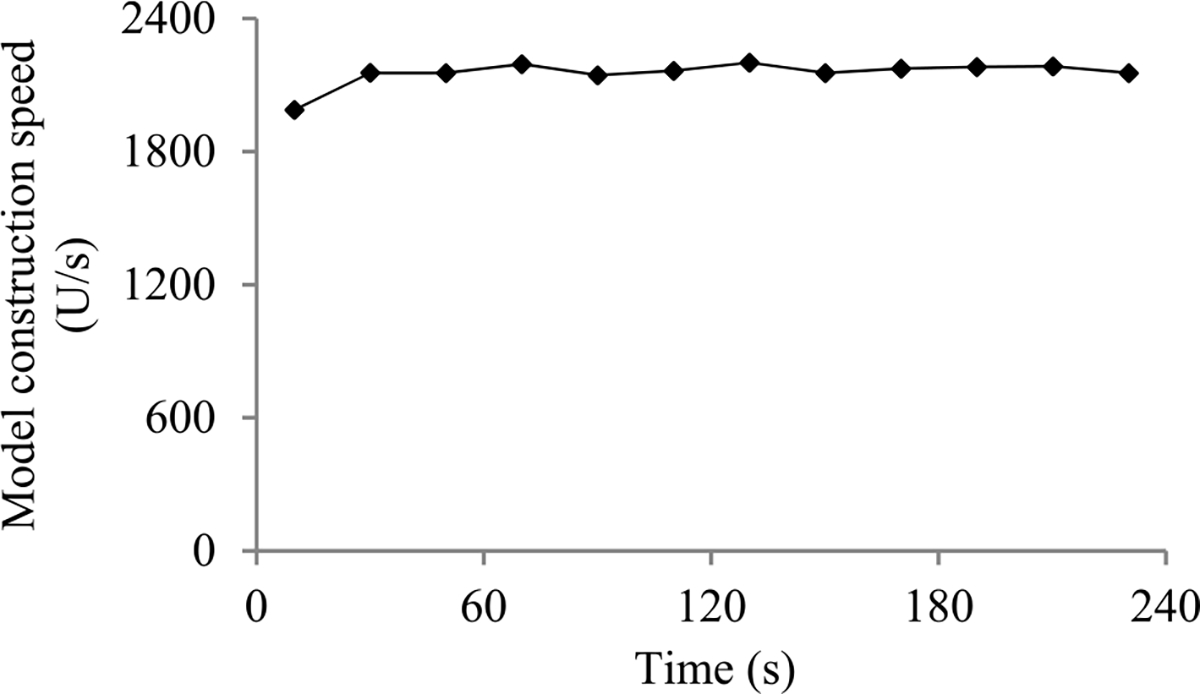
Model construction speed over time (using RMSprop and a constant learning rate to construct the GRU model).

Fig. 15 depicts the model construction speed that the progress indicator observed over time. This speed was relatively stable during the whole model construction process.

**FIGURE 16.**
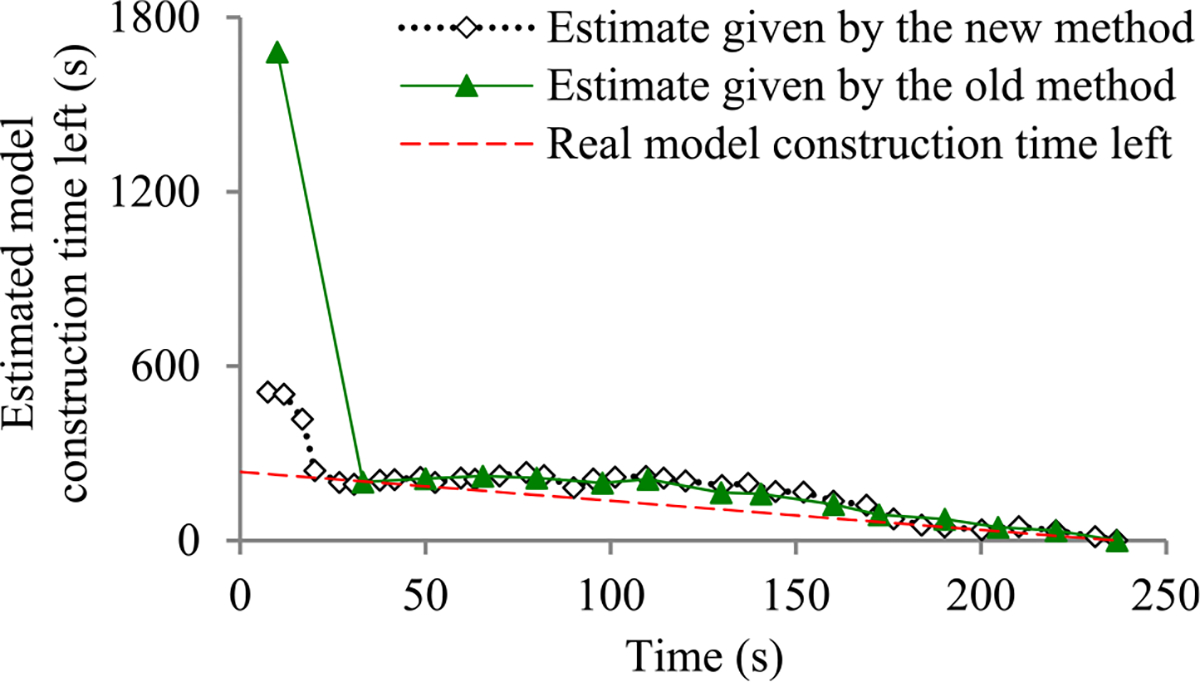
Estimated model construction time left (using RMSprop and a constant learning rate to construct the GRU model).

Fig. 16 depicts the remaining model construction time estimated by the old and the new progress indication methods over time, with the dashed line showing the real model construction time left. The new method reached the stage of giving relatively accurate estimates of the model construction time left much faster than the old method. In fact, the new method’s estimate of the model construction time left was decently accurate during the whole model construction process.

**FIGURE 17.**
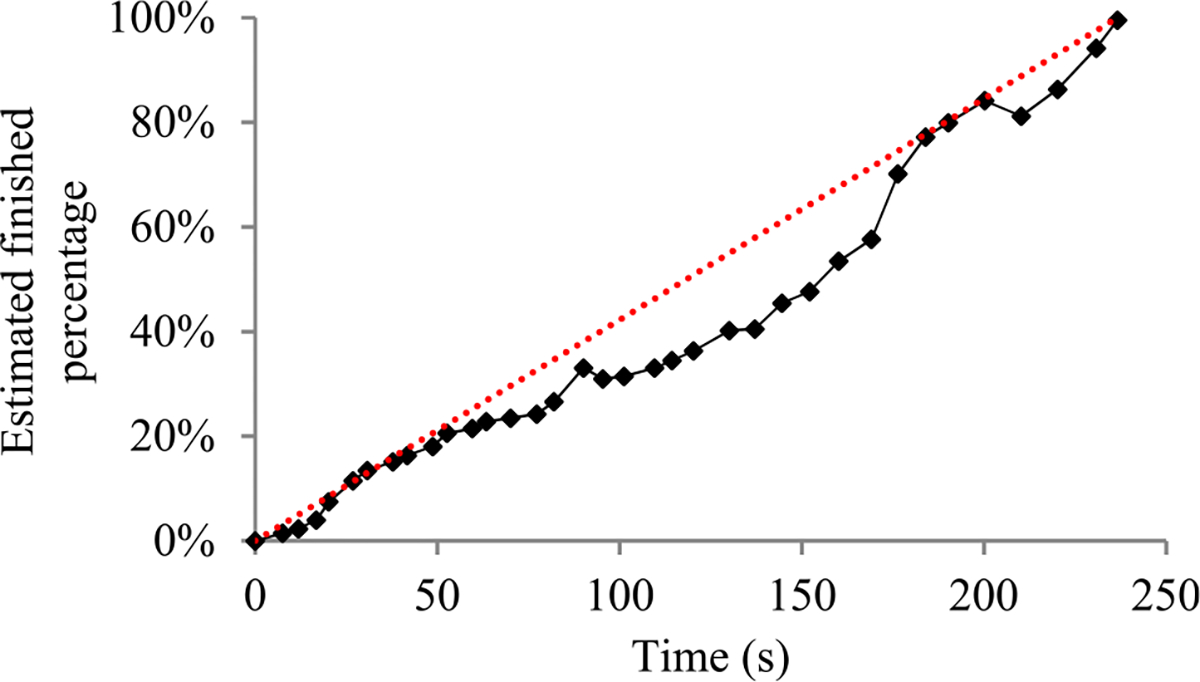
Finished percentage estimated over time (using RMSprop and a constant learning rate to construct the GRU model).

Fig. 17 depicts the progress indicator’s estimate over time of the finished percentage of model construction work. The curve showing the estimated finished percentage is reasonably close to the diagonal dotted line linking the upper right and the lower left corners.

### E. TEST RESULTS FOR APPLYING AN EXPONENTIAL DECAY METHOD TO THE LEARNING RATE

This section presents the test results for applying an exponential decay method to the learning rate. We set the constant *ρ* regulating the decay rate of the learning rate to 0.05.

#### 1) TEST RESULTS FOR CONSTRUCTING GOOGLENET

**FIGURE 18.**
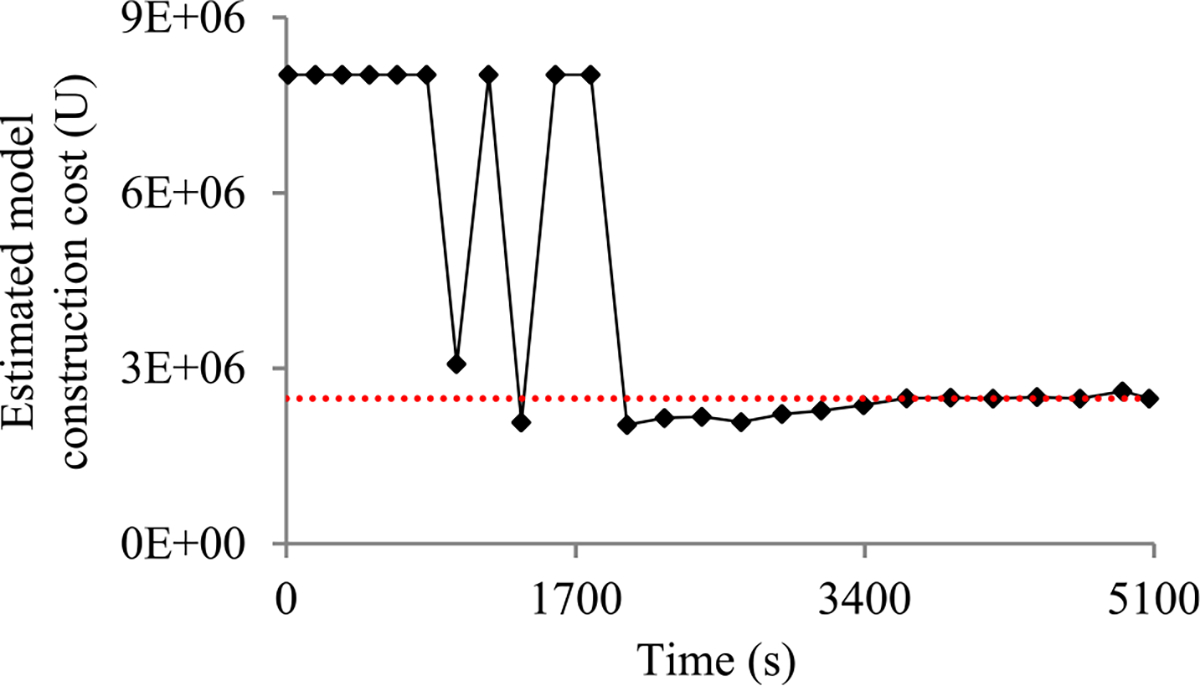
Model construction cost estimated over time (using Adam and applying an exponential decay method to the learning rate to construct GoogLeNet).

**FIGURE 19.**
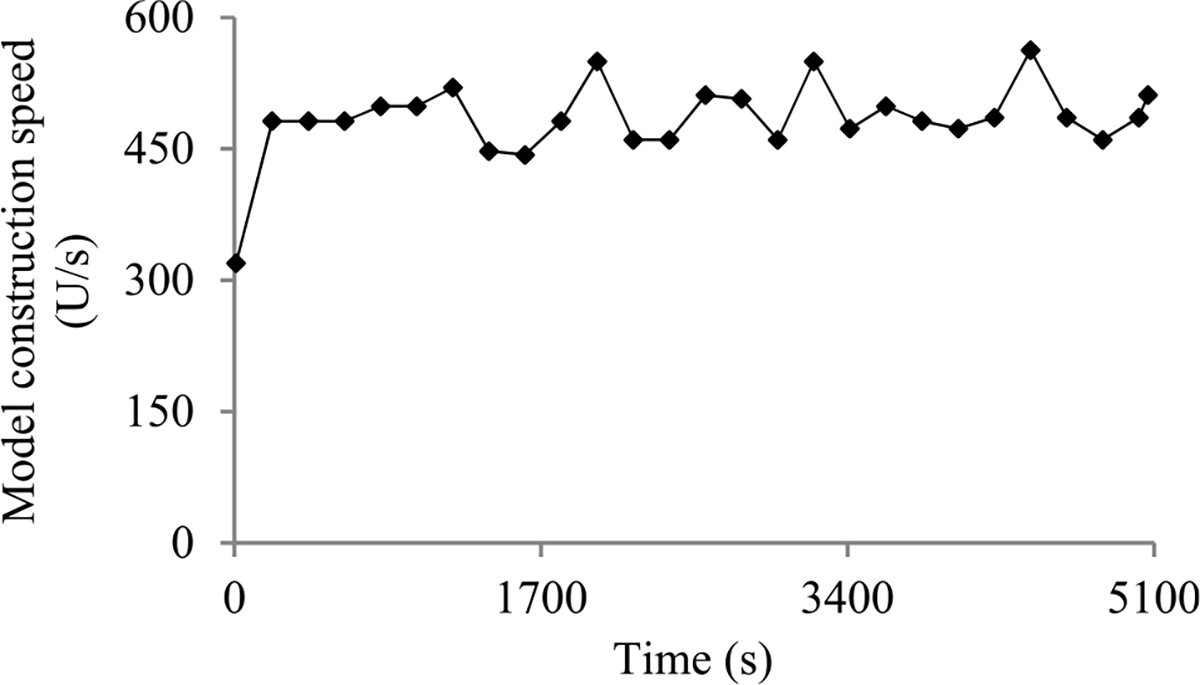
Model construction speed over time (using Adam and applying an exponential decay method to the learning rate to construct GoogLeNet).

**FIGURE 20.**
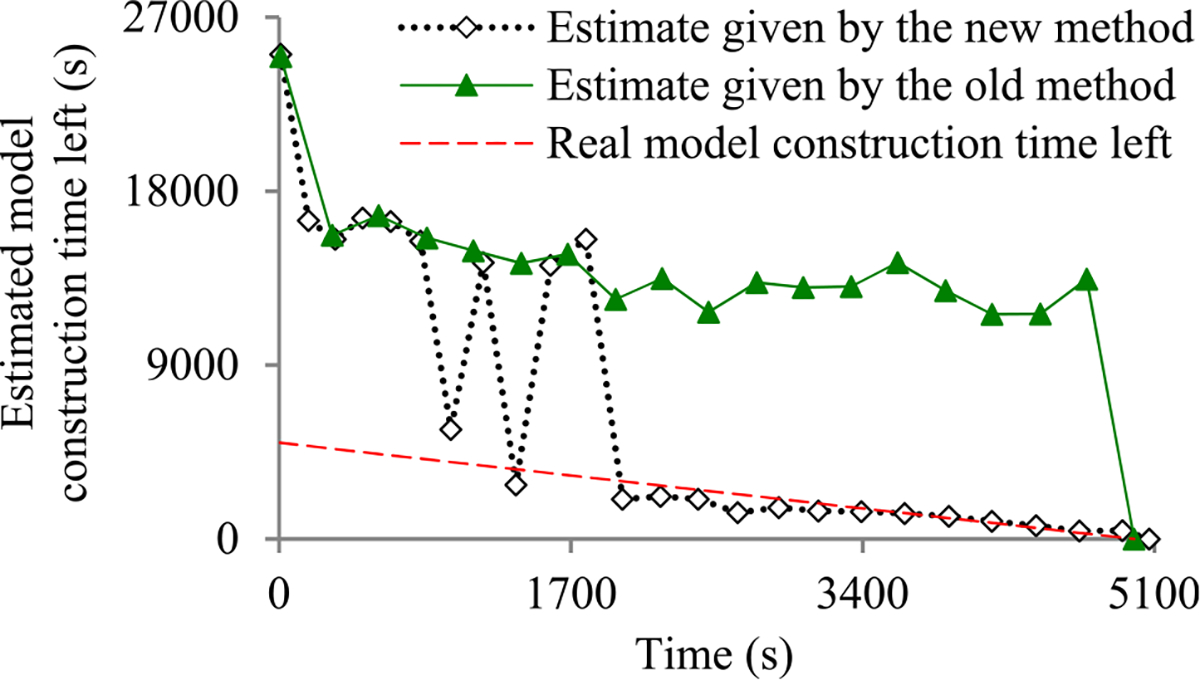
Estimated model construction time left (using Adam and applying an exponential decay method to the learning rate to construct GoogLeNet).

**FIGURE 21.**
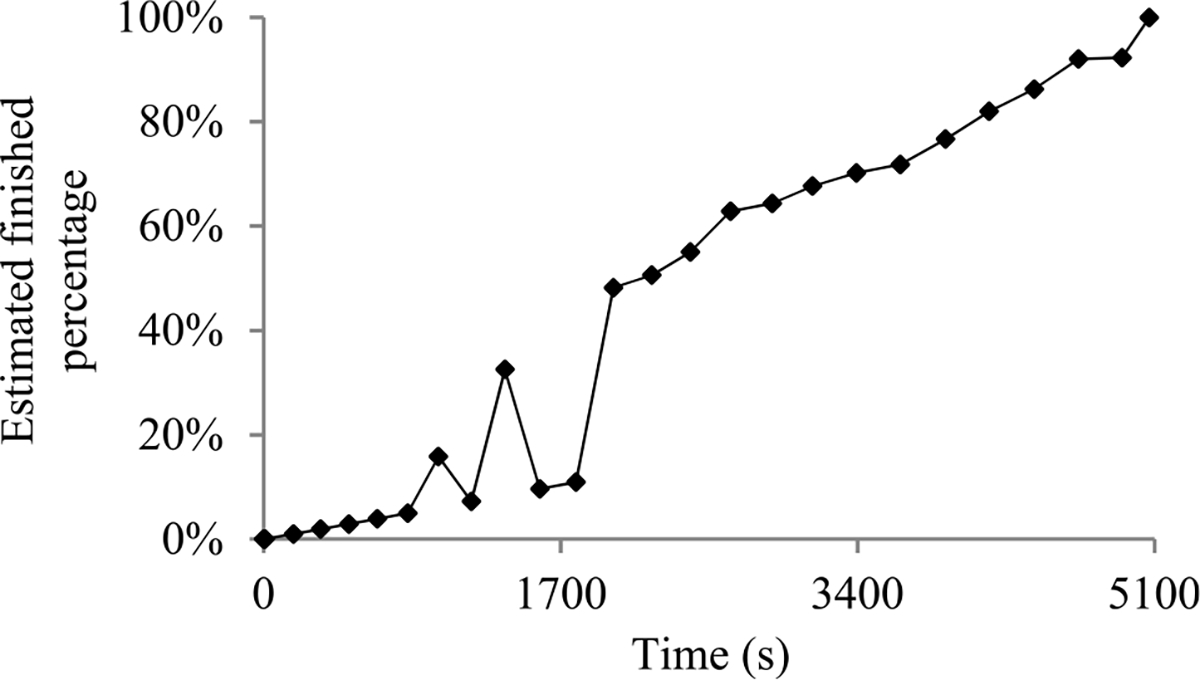
Finished percentage estimated over time (using Adam and applying an exponential decay method to the learning rate to construct GoogLeNet).

In the test, we used the Adam optimization algorithm and applied an exponential decay method to the learning rate to construct GoogLeNet. Fig. 18–21 depict the results for this test. From 0 to 2,002 seconds, the model construction cost estimated by the new progress indication method oscillated and differed notably from the real model construction cost most of the time. This difference led to inaccurate estimates of the model construction time left and the percentage of model construction work finished. After 2,002 seconds, the new progress indication method gave more accurate progress estimates. The new method reached the stage of giving relatively accurate estimates of the model construction time left much faster than the old method.

#### 2) TEST RESULTS FOR CONSTRUCTING THE GRU MODEL

**FIGURE 22.**
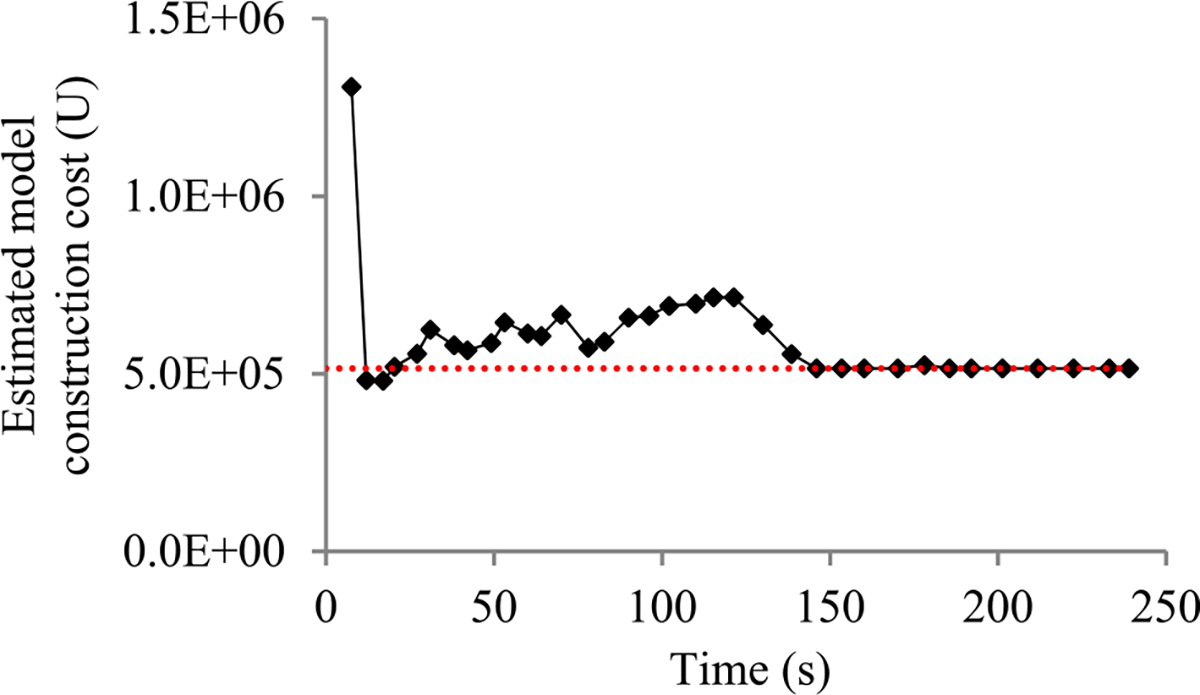
Model construction cost estimated over time (using RMSprop and applying an exponential decay method to the learning rate to construct the GRU model).

**FIGURE 23.**
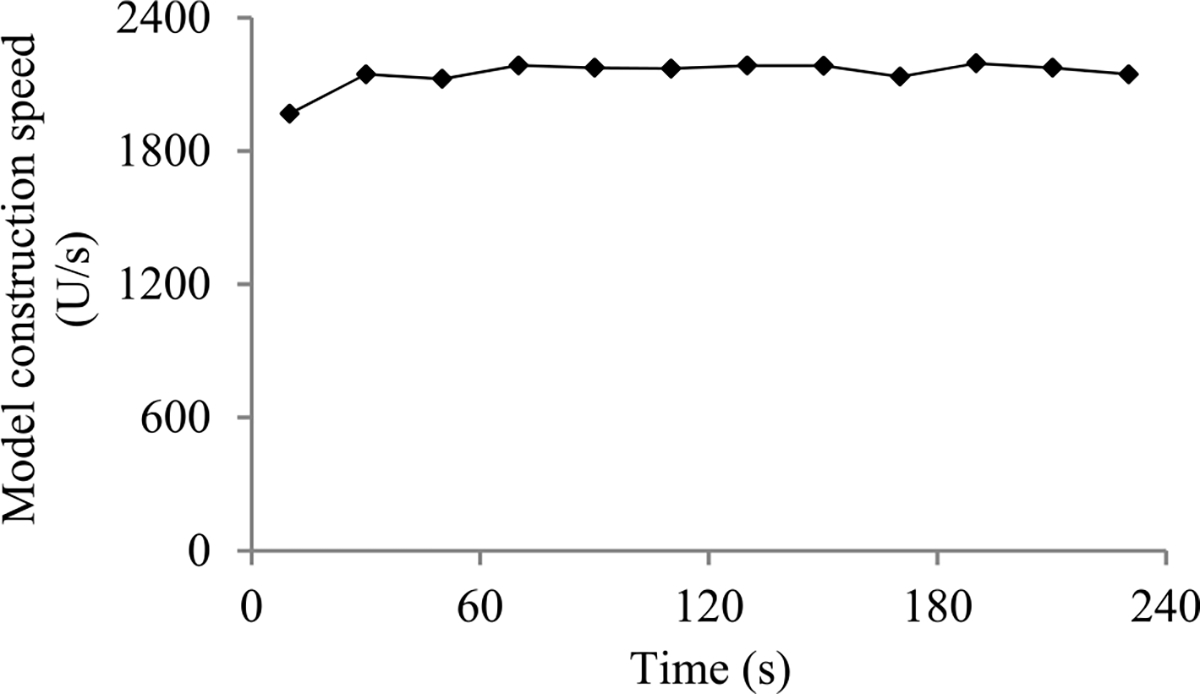
Model construction speed over time (using RMSprop and applying an exponential decay method to the learning rate to construct the GRU model).

**FIGURE 24.**
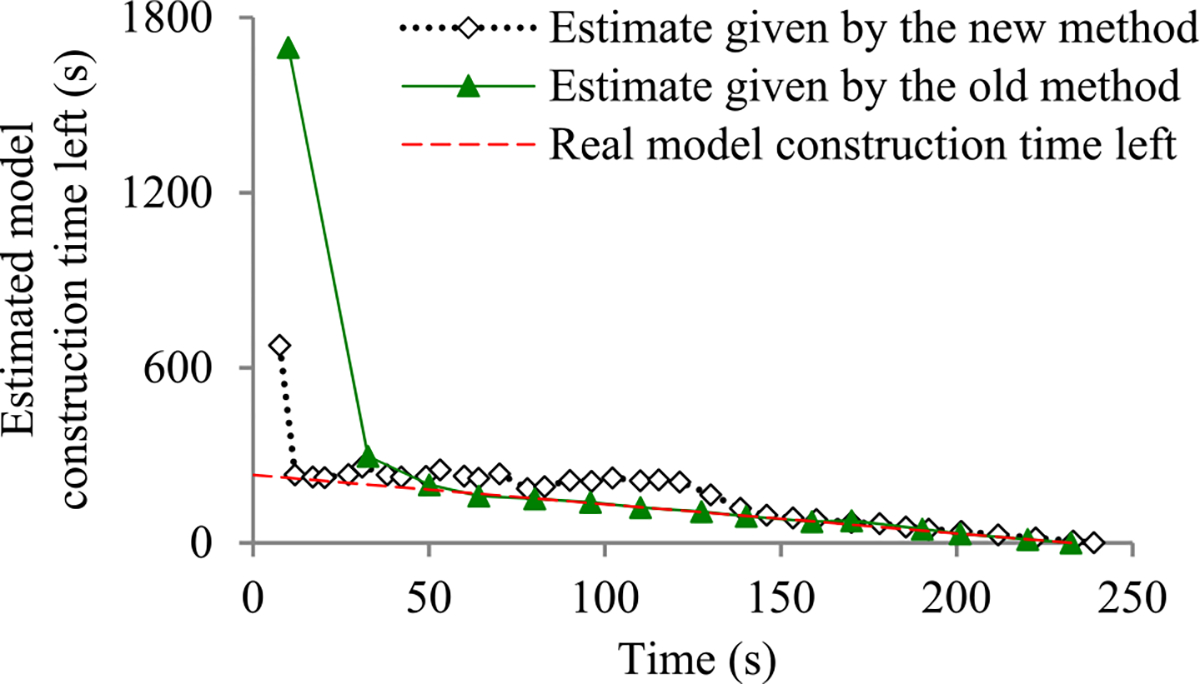
Estimated model construction time left (using RMSprop and applying an exponential decay method to the learning rate to construct the GRU model).

**FIGURE 25.**
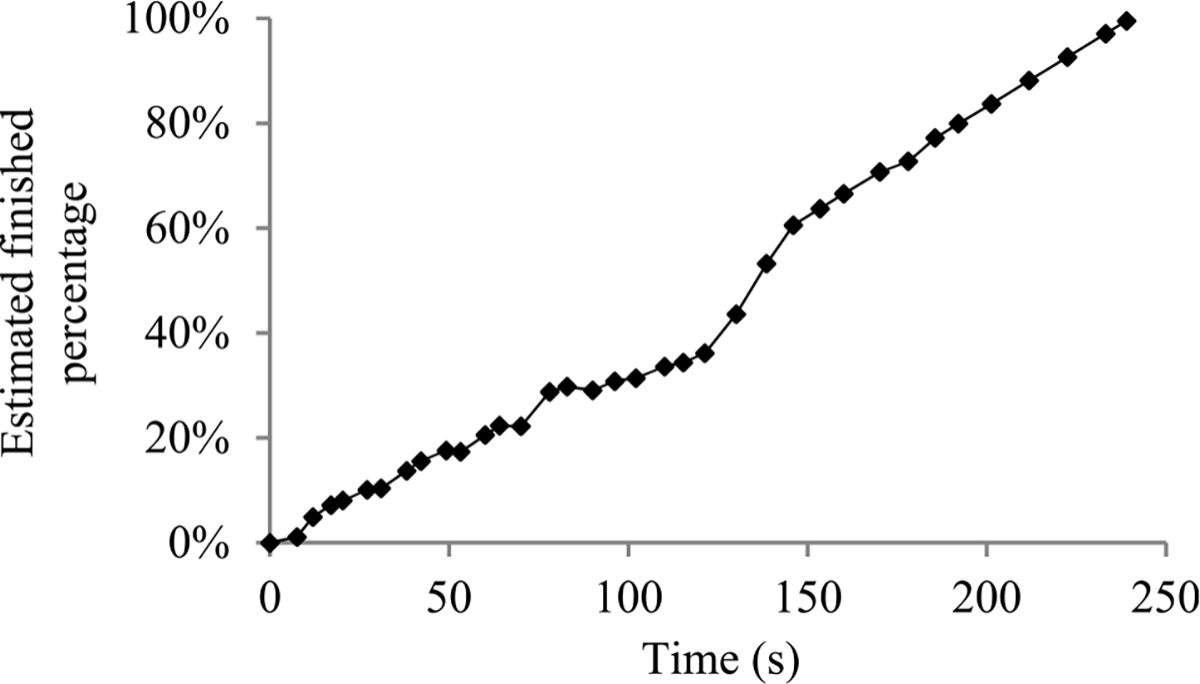
Finished percentage estimated over time (using RMSprop and applying an exponential decay method to the learning rate to construct the GRU model).

In the test, we used the RMSprop optimization algorithm and applied an exponential decay method to the learning rate to construct the GRU model. Fig. 22–25 depict the results for this test, showing that our new progress indication method gave decently accurate estimates during most of the model construction process. The new method reached the stage of giving relatively accurate estimates of the model construction time left much faster than the old method.

### F. TEST RESULTS FOR APPLYING A STEP DECAY METHOD TO THE LEARNING RATE TO CONSTRUCT GOOGLENET

**FIGURE 26.**
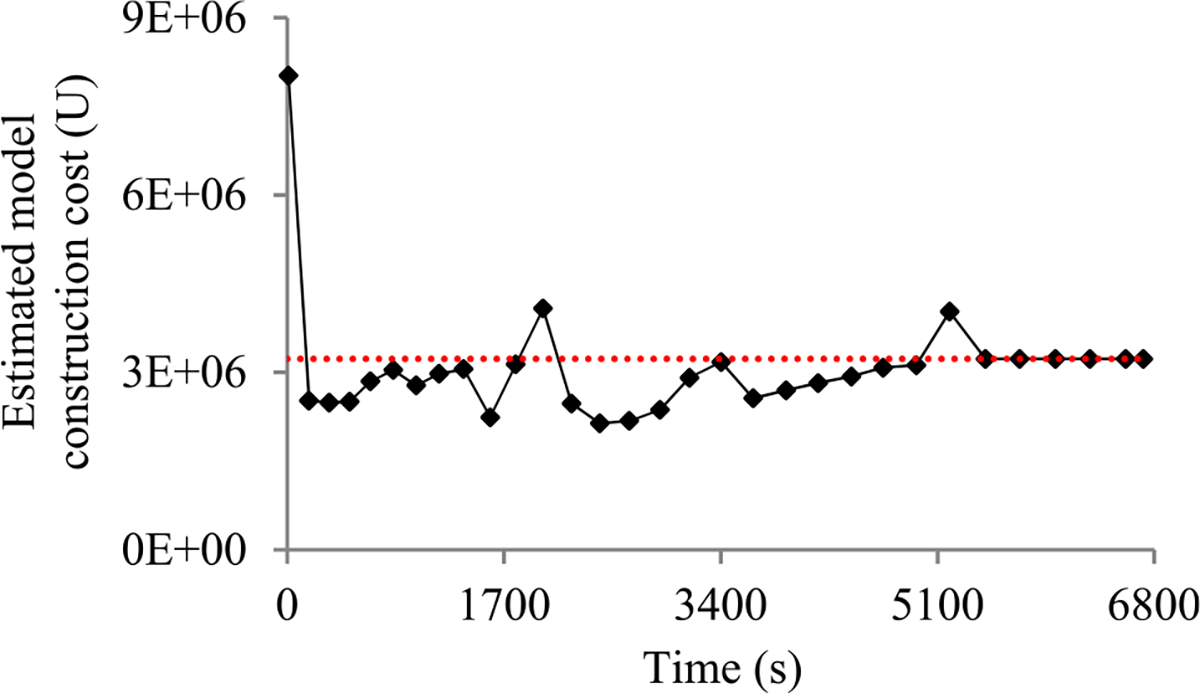
Model construction cost estimated over time (using Adam and applying a step decay method to the learning rate to construct GoogLeNet).

**FIGURE 27.**
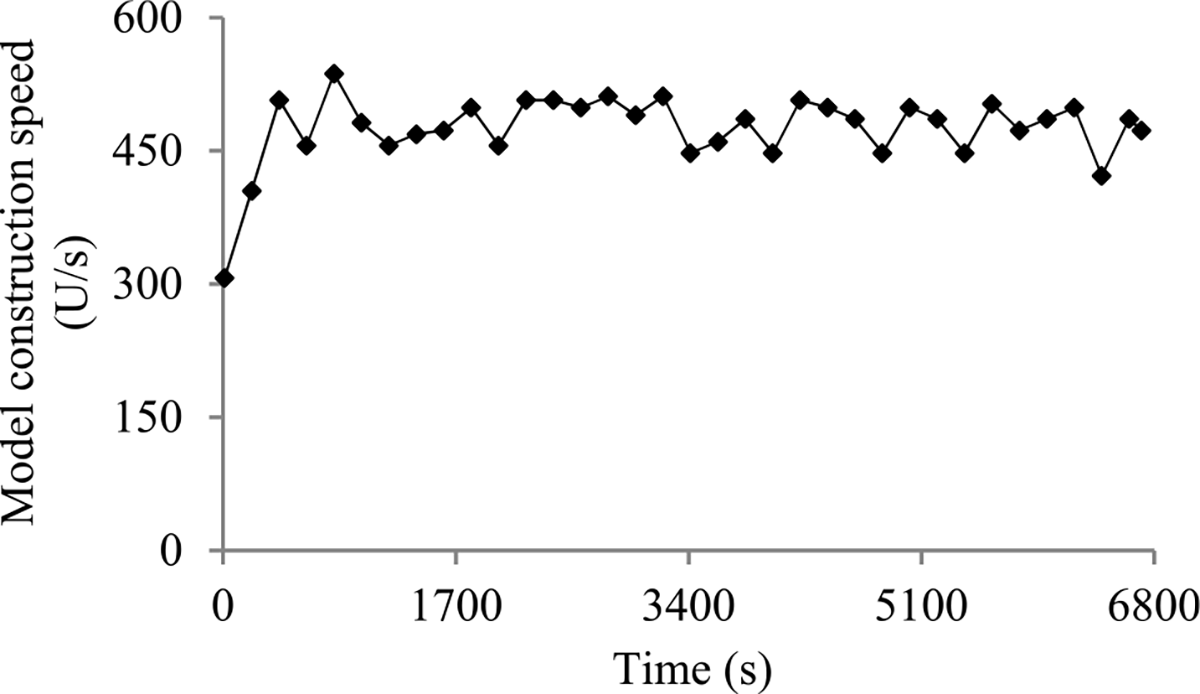
Model construction speed over time (using Adam and applying a step decay method to the learning rate to construct GoogLeNet).

**FIGURE 28.**
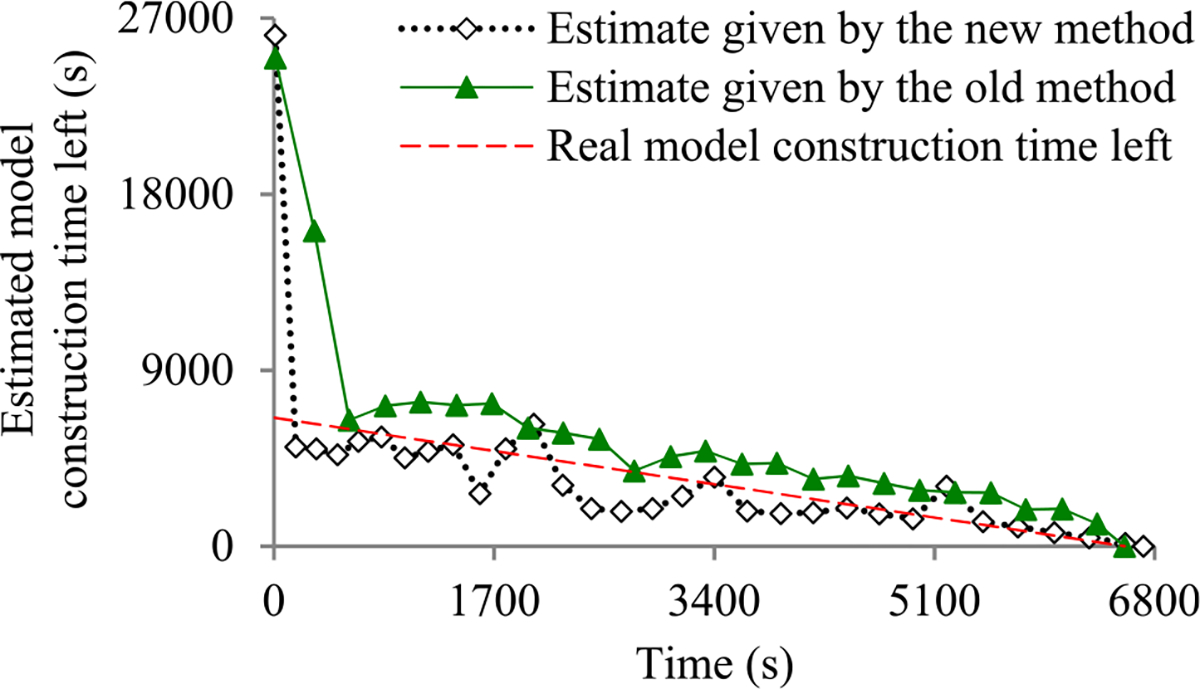
Estimated the model construction time left (using Adam and applying a step decay method to the learning rate to construct GoogLeNet).

**FIGURE 29.**
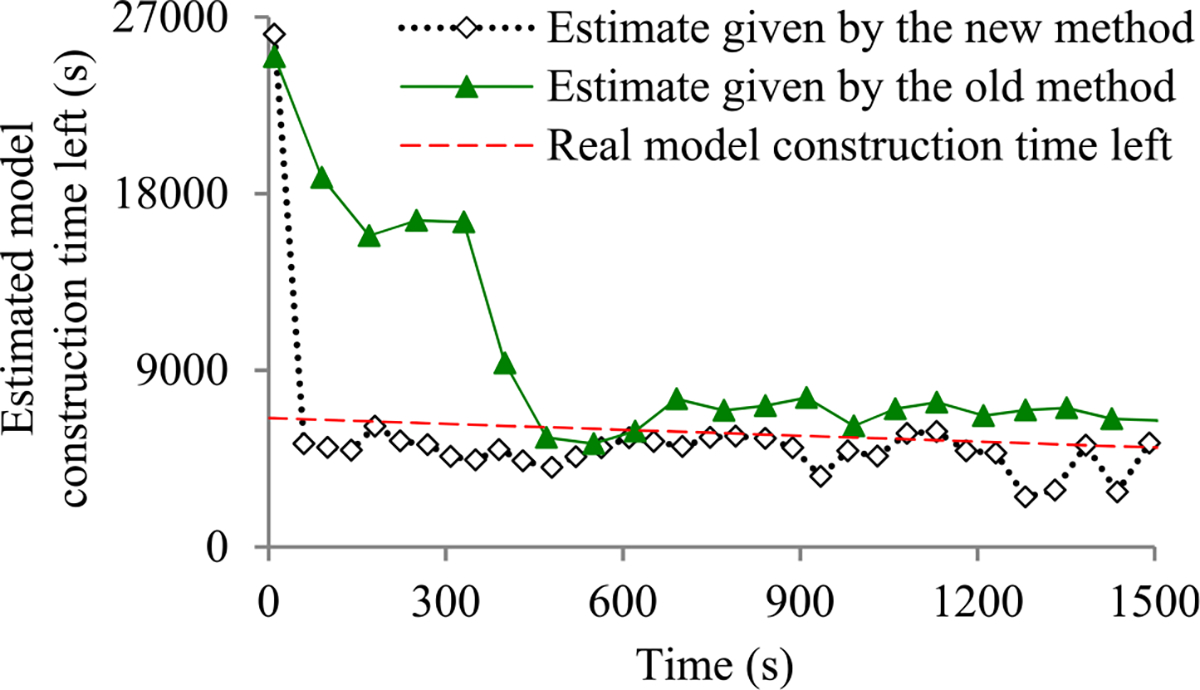
Estimate of the model construction time left at the early stage of model construction (using Adam and applying a step decay method to the learning rate to construct GoogLeNet).

**FIGURE 30.**
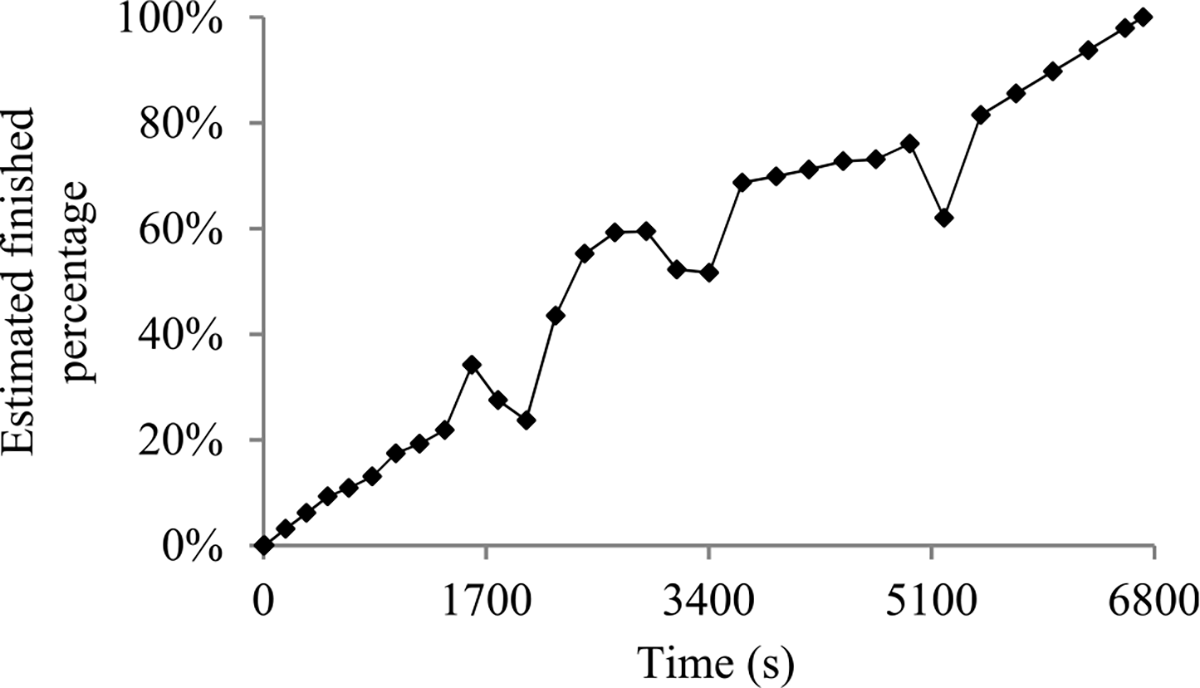
Finished percentage estimated over time (using Adam and applying a step decay method to the learning rate to construct GoogLeNet).

This section presents the test results for adopting the Adam optimization algorithm and applying a step decay method to the learning rate to construct GoogLeNet. We cut the learning rate from 10^−3^ to 10^−4^ at the start of the 64-th epoch, and subsequently to 10^−5^ at the start of the 115-th epoch. In the test, early stopping happened on the first piece of the validation curve. Fig. 26–30 present the test results, which are akin to those presented in Fig. 9–13.

### G. SUMMARY OF THE PERFORMANCE TEST RESULTS

In summary, our experiments show that compared with using our prior progress indication method, using the new method reduces the progress indicator’s prediction error. Moreover, the new method enables us to obtain relatively accurate progress estimates faster with a low overhead.

## V. RELATED WORK

This section provides a brief review of the related work. Our prior paper [6] provides a detailed discussion of the related work.

### A. SOPHISTICATED PROGRESS INDICATORS

Several research groups have proposed sophisticated progress indicators for static program analysis [39], software model checking [40], program compilation [41], database queries [7], [38], [42]–[44], MapReduce jobs [45], [46], subgraph queries [47], and automatic machine learning model selection [48], [49]. In addition, for construction machine learning models, we have created sophisticated progress indicators for random forest, decision tree, as well as neural network [6], [8], [50].

### B. ESTIMATING THE CONSTRUCTION TIME OF DEEP LEARNING MODELS

To estimate the running time of an epoch before the construction of a deep learning model begins, Justus *et al.* [51] developed a meta learning approach that uses multiple features of the computing resources, the present deep learning model, and the training data set employed to construct another deep learning model. That approach projects neither the amount of time nor the count of epochs required to construct a deep learning model.

To project the amount of time required to construct a deep learning model before model construction begins, researchers have developed multiple methods including meta learning employing support vector regression [52], meta learning employing Multivariate Adaptive Regression Splines [53], meta learning employing polynomial regression [54], and Bayesian optimization [55]. The estimates given by these methods are not kept being refined, are often inaccurate, and can diverge greatly from the real model construction time on a loaded computer. In comparison, our progress indication method for deep learning model construction keeps refining its estimates and considers the load on the computer when projecting the model construction time left.

### C. COMPLEXITY ANALYSIS FOR CONSTRUCTING NEURAL NETWORKS

Many researchers have studied the time complexity of constructing a neural network [56, Ch. 24], [57], [58]. However, the time complexity information gives no estimate of the model construction time on a loaded computer and is insufficient for us to develop progress indicators. Typically, time complexity considers neither data properties that affect the model construction cost nor the coefficients and the lower order terms required to predict the model construction cost. A good progress indicator should keep refining its estimated model construction cost during model construction.

### D. RELATIONSHIP BETWEEN THE VARIANCE OF A MACHINE LEARNING MODEL’S ERROR RATE AND THE DATA SET SIZE

For a toy machine learning model not used in the real world, Hutter [59] derived the relationship between the variance of the model’s generalization error and the training set size. In comparison, for deep learning models used in the real world, we derive the relationship between the validation error’s variance and the validation set size.

## VI. DIRECTIONS FOR FUTURE WORK

In this section, we outline some directions for future work.

This work does not give any upper bound for the progress indicator’s projection errors of the model construction cost. To derive such upper bounds in the future, we could employ an approach that is akin to the approach used by Chaudhuri *et al.* [60] for progress indication for executing database queries.

Both our prior work [8] and this work use the same single early stopping condition to do a case study to demonstrate that it is feasible to build sophisticated progress indicators for constructing deep learning models. Besides this early stopping condition, many other early stopping conditions exist [1], [61]–[63]. In the future, we plan to investigate how our present progress indication techniques work for some other popular early stopping conditions and whether our present techniques require any changes to work well for those conditions.

This work focuses on using deep learning for classification. Deep learning can also be used for regression. We could adopt the progress indication method given in our prior paper [8] to handle deep learning regression models. However, as pointed out earlier in this paper, this old method has a shortcoming due to the sparsity of validation points. In the future, we plan to investigate how to revise the new progress indication method given in this paper to handle deep learning regression models. When constructing a deep learning classification model, the validation error given the model’s generalization error follows a discrete distribution linked to a binomial distribution. This is used in Section III-D to derive the relationship between the random noise’s variance and the size of the actual validation set used at the validation point. In comparison, when constructing a deep learning regression model, the validation error given the model’s generalization error follows a continuous distribution. Accordingly, to enable the new progress indication method to handle regression models, we need to derive a different relationship between the random noise’s variance and the size of the actual validation set used at the validation point.

## VII. CONCLUSION

In this paper, we propose a new progress indication method for constructing deep learning models that permits early stopping. By judiciously inserting extra validation points between the original validation points and revising the predicted model construction cost at both the original and the added validation points, this new method could address our prior method’s shortcoming of having a long delay in obtaining relatively accurate progress estimates for the model construction process. Our experimental results show that compared with using our prior method, using this new method not only greatly reduces the progress indicator’s prediction error of the model construction time left, but also enables us to obtain relatively accurate progress estimates faster.

## Supplementary Material

supplemental

## Figures and Tables

**TABLE 1.** The differences between our prior and our new progress indication methods.

Criterion	Our prior progress indication method	Our new progress indication method
Whether extra validation points are inserted between the original validation points (section III-B)	No	Yes
Whether at each validation point, the actual validation set used has the same count of data instances (section III-C)	Yes	No
Whether the relationship between the random noise’s variance and the size of the actual validation set used at the validation point is used (section III-D)	No	Yes
Whether maximum likelihood estimation is used to estimate the trend curve and the variance of the random noise (section III-E)	No	Yes
The minimum number of validation points required to employ the validation curve to re-estimate the count of original validation points required to train the model (section III-A)	3	4

**TABLE 2.** The commonalities between our prior and our new progress indication methods.

Commonality
The validation curve is regarded as the sum of some zero-mean random noise and a smooth trend curve
An inverse power function is used to estimate the trend curve
The approach to conduct Monte Carlo simulation to estimate the count of original validation points required to train the model
The approach to monitor the present model construction speed
The approach to estimate the model construction time left based upon the projected model construction cost left and the present model construction speed

**TABLE 3.** The data sets that we used to test our progress indication method.

Name	Count of data instances that are in the validation set	Count of data instances that are in the training set	Count of classes	Data instance size

CIFAR-10	10,000	50,000	10	image size: 32×32
Feature Set C, 48-h data	6,845	20,532	20	sequence length: 48

**TABLE 4.** For each of GoogLeNet and the GRU model, the *n*_0_, *q*, and *V′* set by the approach given in Section III-B.

Model	*n_0_*	*q*	*V′*

GoogLeNet	11	0.93	726
GRU	5	0.93	683

**TABLE 5.** For each of the 24 tests, the mean as well as the standard deviation of the average prediction error over the five runs for each of the three progress indication methods.

Deep learning model	Learning rate decay method	Optimization algorithm	Average prediction error		
			Progress indication method 1	Progress indication method 2	Progress indication method 3

GoogLeNet	Using a constant learning rate	Adam	0.50±0.10	**0.45**±0.12	0.51±0.14
		RMSprop	0.53±0.25	**0.42**±0.11	**0.42**±0.13
		SGD	0.18±0.03	0.30±0.01	**0.11**±0.01
		AdaGrad	0.17±0.07	0.41±0.02	**0.15**±0.02

	Exponential decay method	Adam	2.46±1.20	1.46±0.66	**0.89**±0.26
		RMSprop	1.20±0.51	0.79±0.19	**0.66**±0.05
		SGD	1.32±0.53	0.97±0.31	**0.70**±0.20
		AdaGrad	1.22±0.29	0.80±0.16	**0.58**±0.09

	Step decay method	Adam	0.45±0.06	0.45±0.07	**0.44**±0.11
		RMSprop	0.73±0.50	**0.54**±0.14	0.57±0.14
		SGD	0.40±0.04	0.49±0.05	**0.34**±0.09
		AdaGrad	**0.35**±0.04	0.44±0.05	0.52±0.09

GRU	Using a constant learning rate	Adam	1.94±0.67	0.54±0.08	**0.48**±0.05
		RMSprop	1.55±0.53	0.60±0.17	**0.52**±0.19
		SGD	0.65±0.08	**0.43**±0.08	0.58±0.12
		AdaGrad	0.93±0.60	0.52±0.03	**0.48**±0.08

	Exponential decay method	Adam	2.40±1.17	0.60±0.18	**0.44**±0.13
		RMSprop	1.27±0.22	0.44±0.13	**0.25**±0.09
		SGD	1.39±0.25	0.93±0.15	**0.51**±0.07
		AdaGrad	1.45±0.62	0.66±0.58	**0.42**±0.26

	Step decay method	Adam	1.94±0.60	0.55±0.18	**0.46**±0.17
		RMSprop	1.59±0.17	0.51±0.07	**0.47**±0.13
		SGD	0.57±0.10	**0.41**±0.08	0.55±0.12
		AdaGrad	1.99±0.50	0.63±0.21	**0.45**±0.16

Over all runs in all tests			1.13±0.84	0.60±0.33	**0.48**±0.21

## LIST OF SYMBOLS

⌈ ⌉: Ceiling function
⌊ ⌋: Floor function
⌊ ⌉: Nearest integer function
a: Scaling factor used in the inverse power function
b: Exponent used in the inverse power function
b_max_: Largest number of batches permitted to train the model
b_l_: Lower bound of the validation error at any validation point
${\hat{b}}_{l}$: Estimate of *b*_*l*_
b_u_: Upper bound of the validation error at any validation point
${\hat{b}}_{u}$: Estimate of *b*_*u*_
B: Count of training instances used in every batch
c: Bias term used in the inverse power function
*c*_0_: Model construction cost that has been incurred when we finish the work at the first original validation point, excluding the progress indicator’s overhead of calculating the validation errors at the added validation points
c_j_: Count of validation instances that are misclassified by the model and in the actual validation set used at the *j*-th validation point
c_v_: Cost to calculate the validation error at the first original validation point
C: Upper threshold of the model construction cost that has been incurred when we finish the work at the fourth validation point
e_j_: The model’s generalization error at the *j*-th validation point
${\hat{e}}_{j}$: Validation error of the model at the *j*-th validation point
${\tilde{e}}_{j}$: Validation error of the model at the *j*-th original validation point
*f*(*q*): A function of *q*
g: Count of batches of model construction between two successive original validation points
*h*(*n*): Present validation point’s sequence number on the present piece of the validation curve
l_j_: Count of validation points that are on the *j*-th piece of the validation curve
*L*(***θ*** | ***y***): Likelihood function: the probability of ***y*** as a function of the parameters ***θ***
m_e_: Largest number of epochs permitted to train the model
n: Present validation point’s sequence number
*n*_0_: Count of validation points added before the first original validation point
n_j_: Count of validation points added between the *j*-th and the (*j* + 1)-th original validation points
n_v_: Count of original validation points required to train the model
p: Patience
p_j_: Percentage increase in the model construction cost that the progress indicator causes during the period from when model construction starts to the time we finish the work at the *j*-th original validation point
*P*_1_: Maximum allowed percentage increase in the model construction cost that the progress indicator causes during the period from when model construction starts to the time we finish the work at the first original validation point
P_v_: Maximum allowed percentage increase in the model construction cost that the progress indicator causes during the period from when model construction starts to the time we finish the work at the *v*_*max*_-th original validation point
q: Constant regulating the decay rate of *n*_*j*_ (0 ≤ *j* ≤ *v*_*max*_ − 1) in the exponential decay schema
*r*_0_: Beginning learning rate adopted in the exponential decay method
r_j_: Learning rate right before the *j*-th validation point
*s*_*k*−1_: Sequence number of the final validation point that is on the prior piece of the validation curve
U: Unit of work
*v*_*k*−1_: At the final validation point appearing on the prior piece of the validation curve, the projected count of both original and added validation points required to train the model
v_max_: Largest number of original validation points permitted to train the model
V: Count of data instances that are in the full validation set
V_j_: Count of data instances that are in the actual validation set used at the *j*-th validation point
V_min_: Minimum number of data instances needed in the randomly sampled subset of the full validation set used at an added validation point
*V*′: Uniform number of data instances that are in the randomly sampled subset of the full validation set used at each added validation point
w: Count of validation points used to estimate *a*, *b*, *c*, and *λ*
*w*′: Largest number of validation points permitted to estimate *a*, *b*, *c*, and *λ*
x_j_: Normalized number of batches of model construction finished before the *j*-th validation point
z: Constant regulating the decay rate of *n*_*j*_ (0 ≤ *j* ≤ *v*_*max*_ − 1) in the linear decay schema
α: The beta distribution’s first shape parameter
β: The beta distribution’s second shape parameter
B(*α*, *β*): Normalization constant in the probability density function of the beta distribution
δ: min_delta
ε_j_: Random noise at the *j*-th validation point
λ: Ratio of the variance of the model’s generalization error to the square of the learning rate
μ_j_: Mean of the model’s generalization error at the *j*-th validation point
$\mu_{j}^{\prime }$: Mean of the beta distribution linking to the *j*-th validation point
${\tilde{\mu }}_{j}$: Mean of a normal distribution linking to the *j*-th validation point
ρ: Constant regulating the decay rate of the learning rate in the exponential decay method
$\sigma_{j}^{2}$: Variance of the model’s generalization error at the *j*-th validation point
$\sigma_{j}^{\prime 2}$: Variance of the beta distribution linking to the *j*-th validation point
${\tilde{\sigma }}_{j}^{2}$: Variance of a normal distribution linking to the *j*-th validation point
τ_v_: Minimum number of validation points required to employ the validation curve to re-estimate the count of original validation points required to train the model
Φ(): Cumulative distribution function of the standard normal distribution
