# Supplementary material for: Improving the Accuracy of Progress Indication for Constructing Deep Learning Models
Source: IEEE Access. Author manuscript; Available in PMC 2022 Jul 21. (PMC9302923; doi:10.1109/access.2022.3181493)
Supplement: supplemental [file NIHMS1817767-supplement-supplemental.pdf]

APPENDIX

The Appendix presents the test results not covered in Section IV.

A. OTHER TEST RESULTS FOR ADOPTING A CONSTANT LEARNING RATE

1) TEST RESULTS FOR CONSTRUCTING GOOGLENET

Adopting the RMSprop optimization algorithm

In the test, we used the RMSprop optimization algorithm and a constant learning rate to construct GoogLeNet. Fig. 31-35 present the test results, which are akin to those presented in Fig. 9-13.

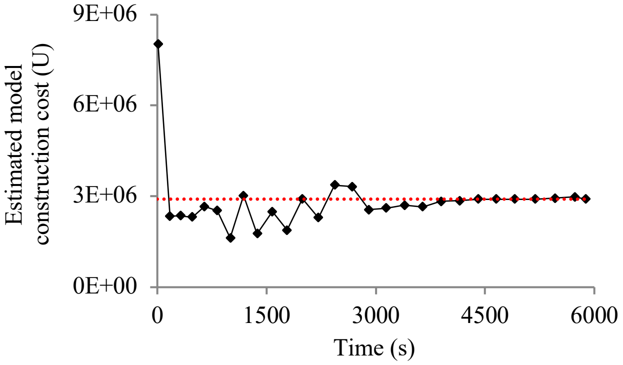

FIGURE 31. Model construction cost estimated over time (using RMSprop and a constant learning rate to construct GoogLeNet).

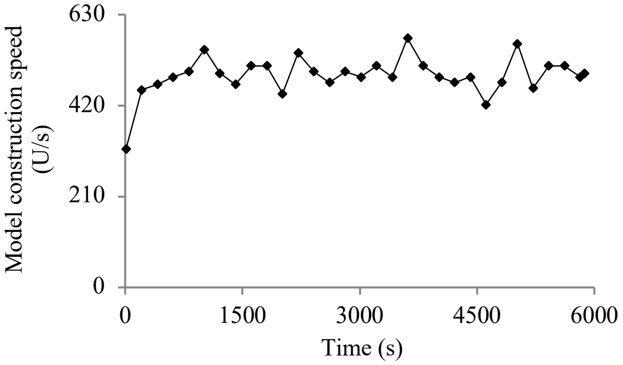

FIGURE 32. Model construction speed over time (using RMSprop and a constant learning rate to construct GoogLeNet).

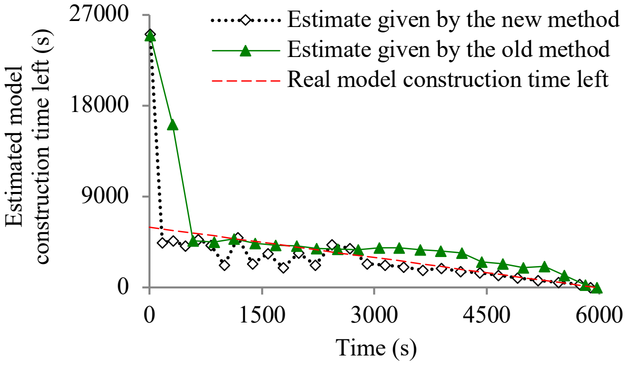

FIGURE 33. Estimated model construction time left (using RMSprop and a constant learning rate to construct GoogLeNet).

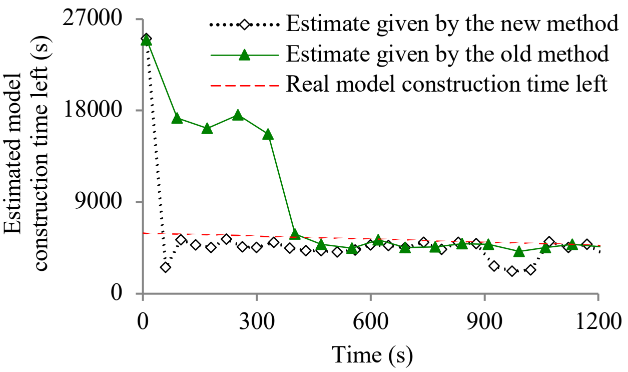

FIGURE 34. Estimate of the model construction time left at the early stage of model construction (using RMSprop and a constant learning rate to construct GoogLeNet).

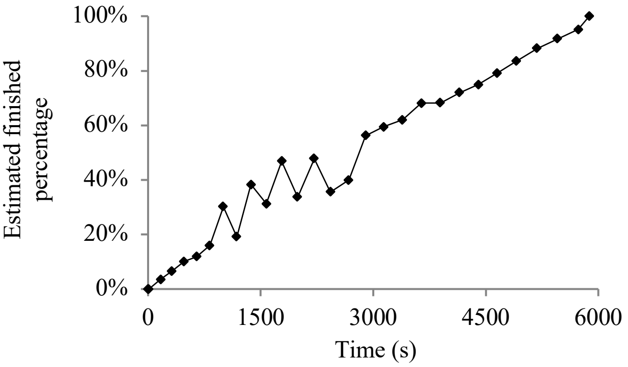

FIGURE 35. Finished percentage estimated over time (using RMSprop and a constant learning rate to construct GoogLeNet).

Adopting the SGD optimization algorithm

In the test, we used the SGD optimization algorithm and a constant learning rate to construct GoogLeNet. Fig. 36-39 show the test results. During the model construction process, the early stopping condition was never fulfilled. Our progress indicator correctly foresaw this and gave accurate estimates during most of the model construction process.

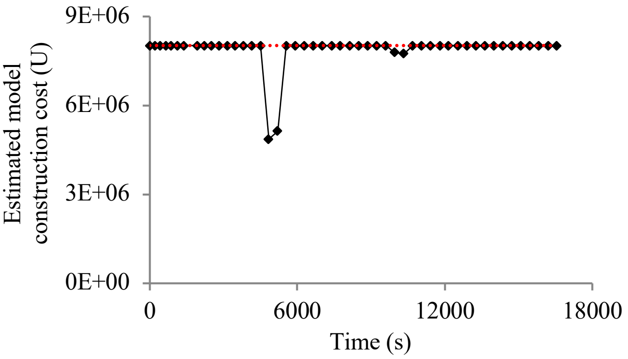

FIGURE 36. Model construction cost estimated over time (using SGD and a constant learning rate to construct GoogLeNet).

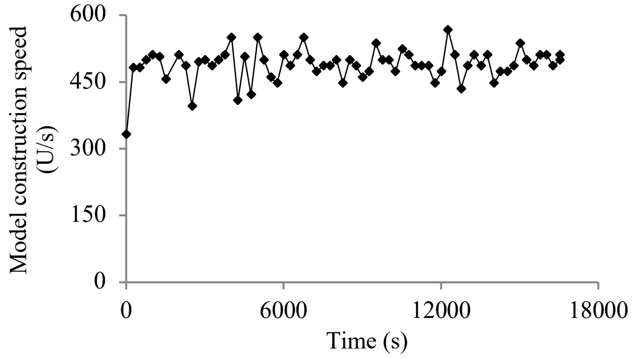

FIGURE 37. Model construction speed over time (using SGD and a constant learning rate to construct GoogLeNet).

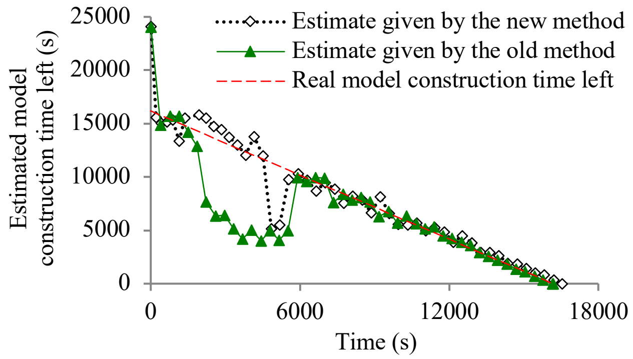

FIGURE 38. Estimated model construction time left (using SGD and a constant learning rate to construct GoogLeNet).

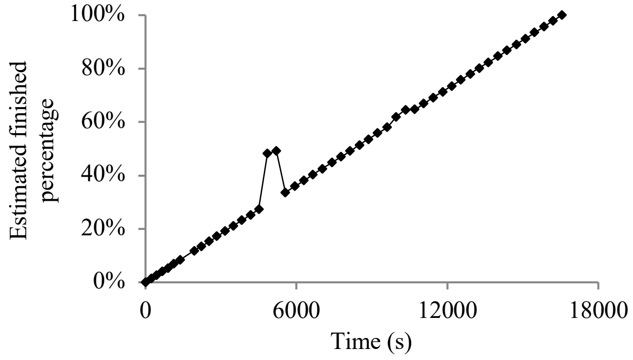

FIGURE 39. Finished percentage estimated over time (using SGD and a constant learning rate to construct GoogLeNet).

Adopting the AdaGrad optimization algorithm

In the test, we used the AdaGrad optimization algorithm and a constant learning rate to construct GoogLeNet. Fig. 40-43 present the test results, which are akin to those presented in Fig. 36-39.

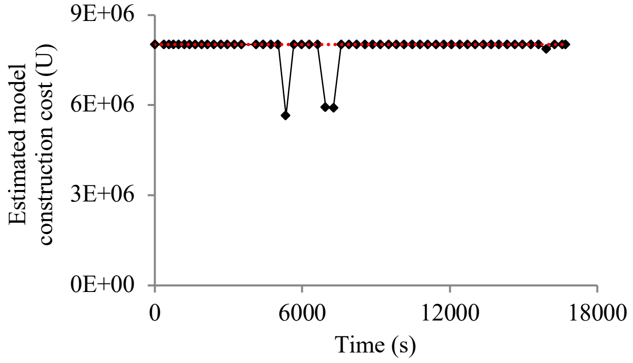

FIGURE 40. Model construction cost estimated over time (using AdaGrad and a constant learning rate to construct GoogLeNet).

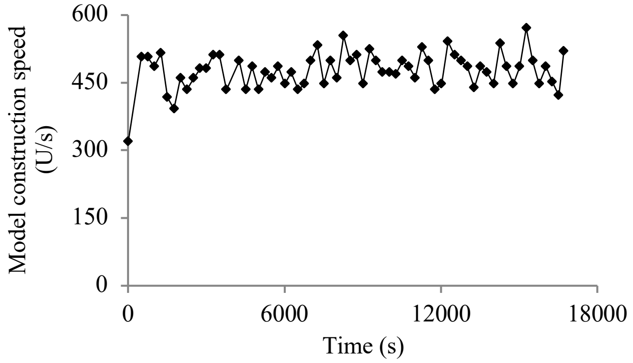

FIGURE 41. Model construction speed over time (using AdaGrad and a constant learning rate to construct GoogLeNet).

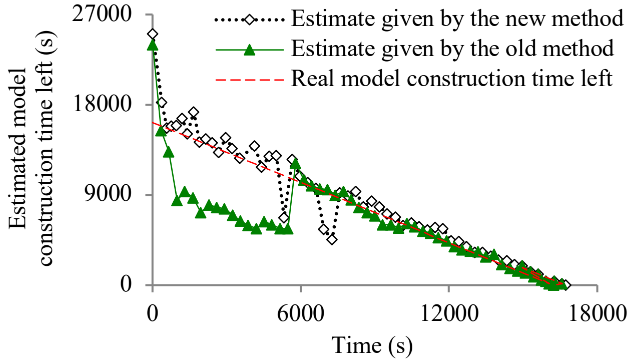

FIGURE 42. Estimated model construction time left (using AdaGrad and a constant learning rate to construct GoogLeNet).

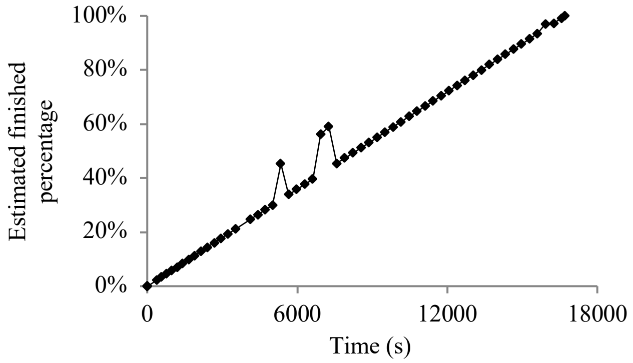

FIGURE 43. **Finished percentage estimated over time (using AdaGrad and a constant learning rate to construct GoogLeNet).**

2) TEST RESULTS FOR CONSTRUCTING THE GRU MODEL

Adopting the Adam optimization algorithm

In the test, we used the Adam optimization algorithm and a constant learning rate to construct the GRU model. Fig. 44-47 present the test results, which are akin to those presented in Fig. 14-17.

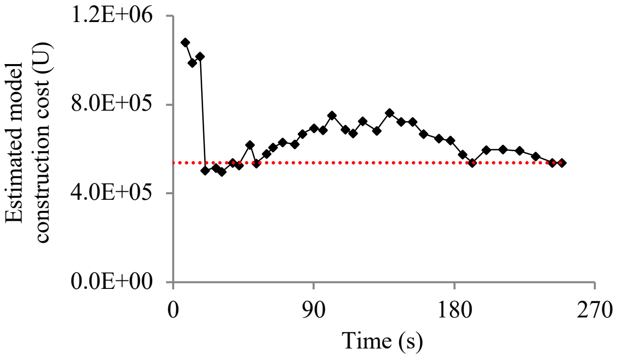

FIGURE 44. **Model construction cost estimated over time (using Adam and a constant learning rate to construct the GRU model).**

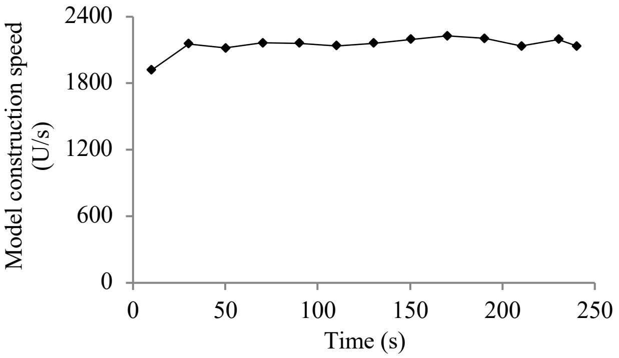

FIGURE 45. **Model construction speed over time (using Adam and a constant learning rate to construct the GRU model).**

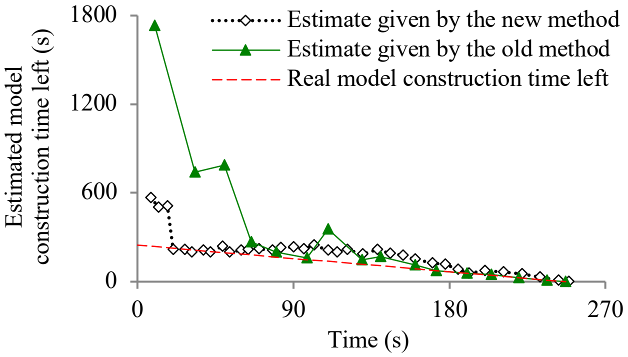

FIGURE 46. **Estimated model construction time left (using Adam and a constant learning rate to construct the GRU model).**

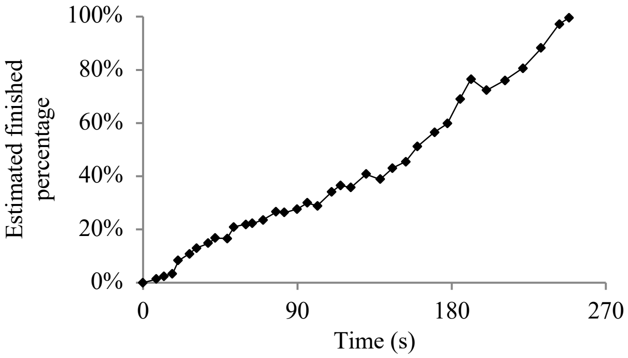

FIGURE 47. **Finished percentage estimated over time (using Adam and a constant learning rate to construct the GRU model).**

Adopting the SGD optimization algorithm

In the test, we used the SGD optimization algorithm and a constant learning rate to construct the GRU model. Fig. 48-52 present the test results, which are relatively akin to those presented in Fig. 14-17.

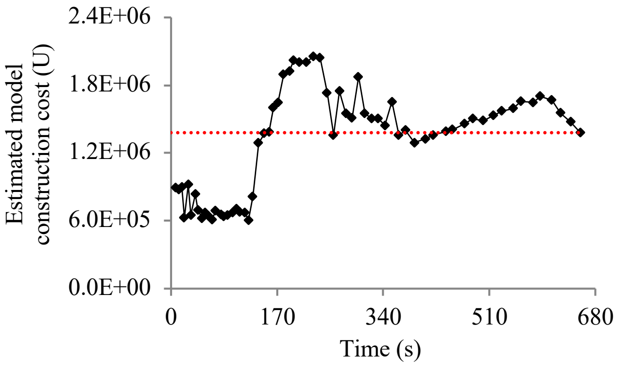

FIGURE 48. **Model construction cost estimated over time (using SGD and a constant learning rate to construct the GRU model).**

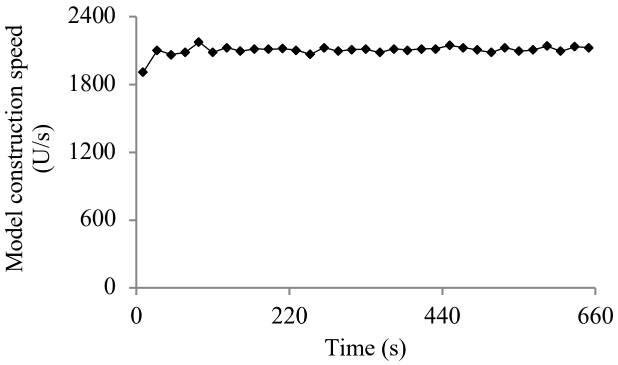

FIGURE 49. **Model construction speed over time (using SGD and a constant learning rate to construct the GRU model).**

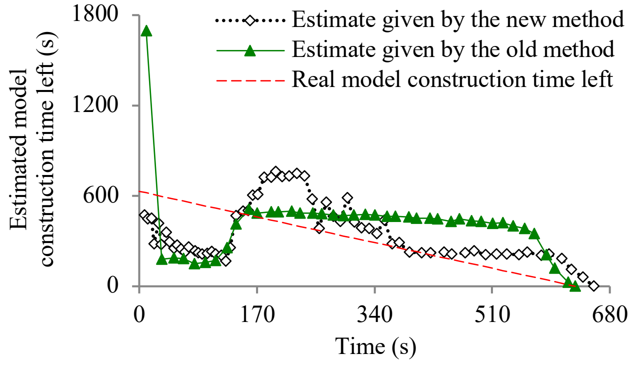

FIGURE 50. Estimated model construction time left (using SGD and a constant learning rate to construct the GRU model).

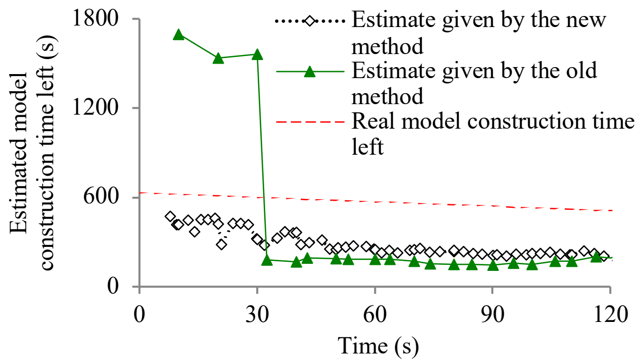

FIGURE 51. Estimate of the model construction time left at the early stage of model construction (using SGD and a constant learning rate to construct the GRU model).

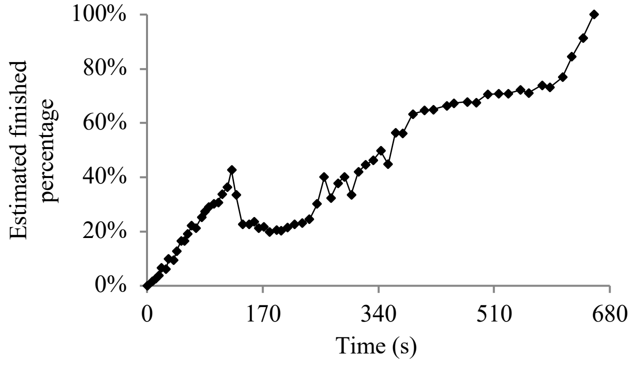

FIGURE 52. Finished percentage estimated over time (using SGD and a constant learning rate to construct the GRU model).

Adopting the AdaGrad optimization algorithm

In the test, we used the AdaGrad optimization algorithm and a constant learning rate to construct the GRU model. Fig. 53-56 present the test results and show that our progress indicator gave decently accurate estimates during most of the model construction process.

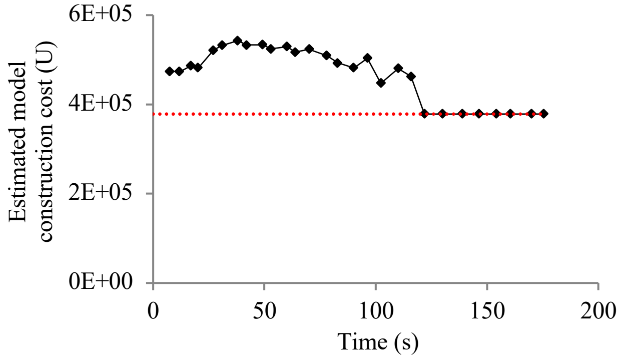

FIGURE 53. Model construction cost estimated over time (using AdaGrad and a constant learning rate to construct the GRU model).

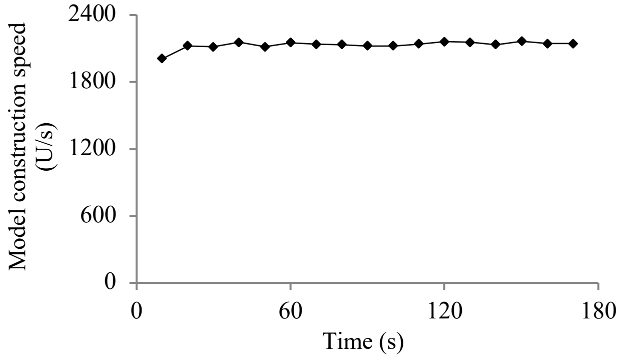

FIGURE 54. Model construction speed over time (using AdaGrad and a constant learning rate to construct the GRU model).

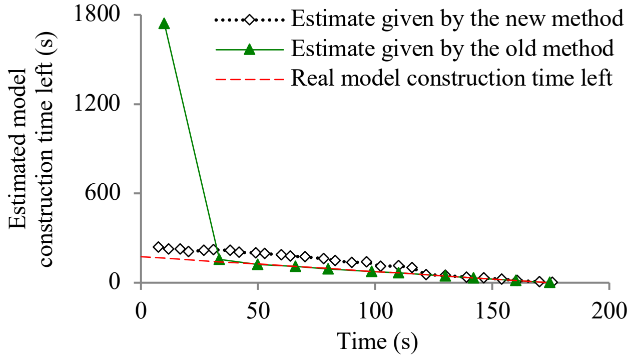

FIGURE 55. Estimated model construction time left (using AdaGrad and a constant learning rate to construct the GRU model).

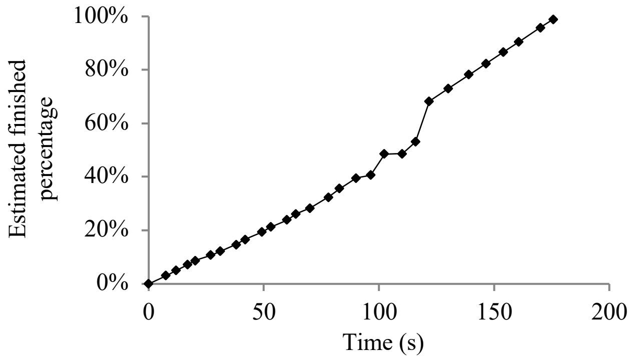

FIGURE 56. **Finished percentage estimated over time (using AdaGrad and a constant learning rate to construct the GRU model).**

B. OTHER TEST RESULTS FOR APPLYING AN EXPONENTIAL DECAY METHOD TO THE LEARNING RATE

1) TEST RESULTS FOR CONSTRUCTING GOOGLENET

Adopting the RMSprop optimization algorithm

In the test, we used the RMSprop optimization algorithm and applied an exponential decay method to the learning rate to construct GoogLeNet. Fig. 57-60 present the test results, which are akin to those presented in Fig. 18-21.

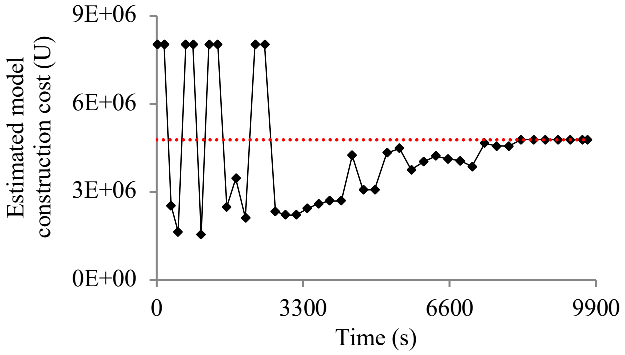

FIGURE 57. **Model construction cost estimated over time (using RMSprop and applying an exponential decay method to the learning rate to construct GoogLeNet).**

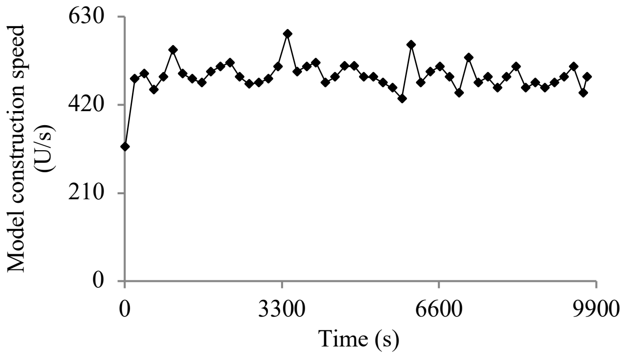

FIGURE 58. **Model construction speed over time (using RMSprop and applying an exponential decay method to the learning rate to construct GoogLeNet).**

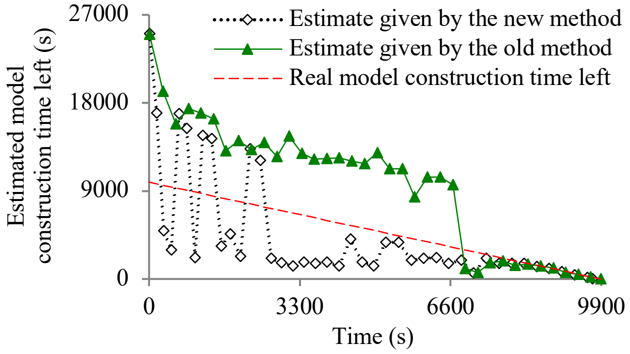

FIGURE 59. **Estimated model construction time left (using RMSprop and applying an exponential decay method to the learning rate to construct GoogLeNet).**

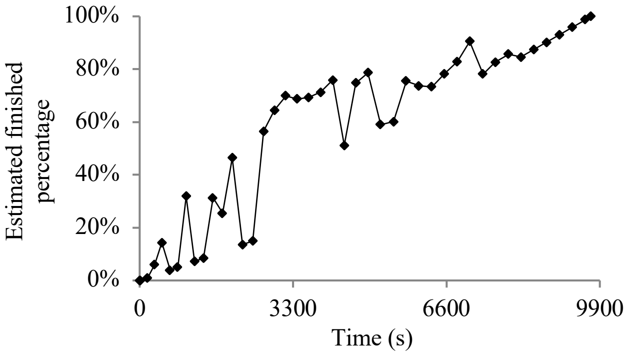

FIGURE 60. **Finished percentage estimated over time (using RMSprop and applying an exponential decay method to the learning rate to construct GoogLeNet).**

Adopting the SGD optimization algorithm

In the test, we used the SGD optimization algorithm and applied an exponential decay method to the learning rate to construct GoogLeNet. Fig. 61-64 present the test results, which are akin to those presented in Fig. 18-21.

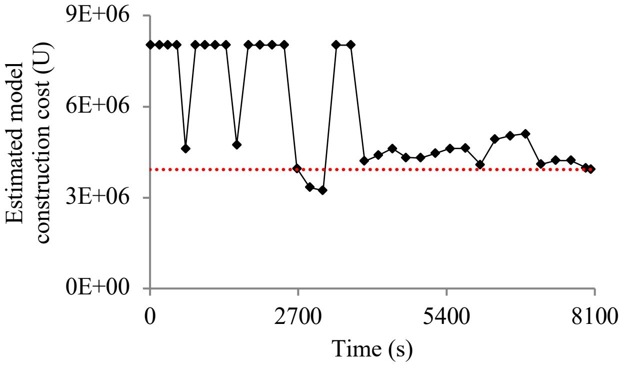

FIGURE 61. **Model construction cost estimated over time (using SGD and applying an exponential decay method to the learning rate to construct GoogLeNet).**

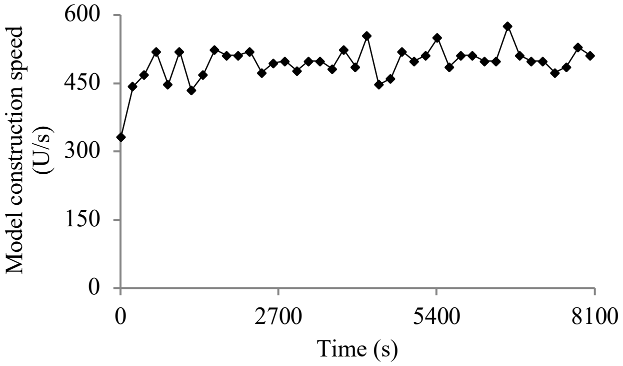

FIGURE 62. **Model construction speed over time (using SGD and applying an exponential decay method to the learning rate to construct GoogLeNet).**

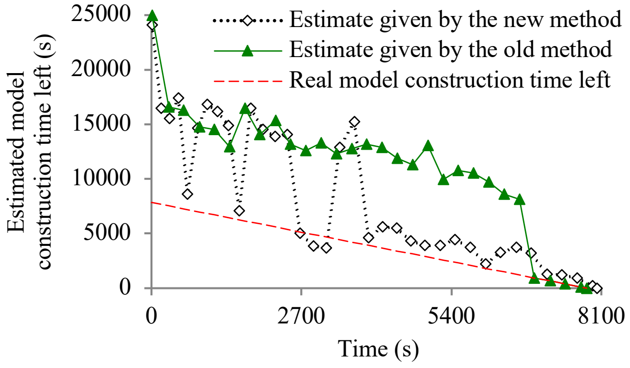

FIGURE 63. **Estimated model construction time left (using SGD and applying an exponential decay method to the learning rate to construct GoogLeNet).**

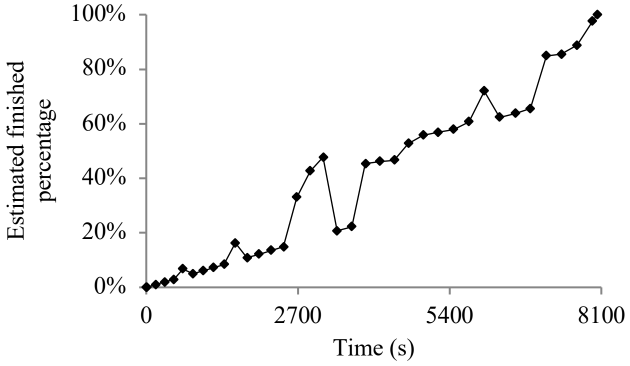

FIGURE 64. **Finished percentage estimated over time (using SGD and applying an exponential decay method to the learning rate to construct GoogLeNet).**

Adopting the AdaGrad optimization algorithm

In the test, we used the AdaGrad optimization algorithm and applied an exponential decay method to the learning rate to construct GoogLeNet. Fig. 65-68 present the test results, which are akin to those presented in Fig. 18-21.

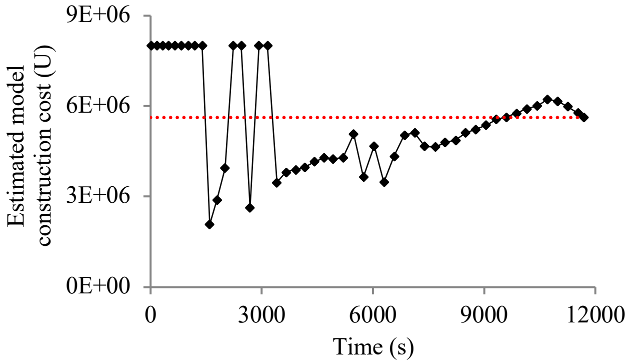

FIGURE 65. **Model construction cost estimated over time (using AdaGrad and applying an exponential decay method to the learning rate to construct GoogLeNet).**

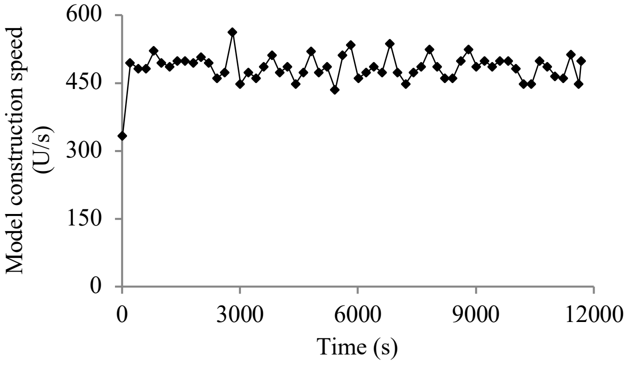

FIGURE 66. **Model construction speed over time (using AdaGrad and applying an exponential decay method to the learning rate to construct GoogLeNet).**

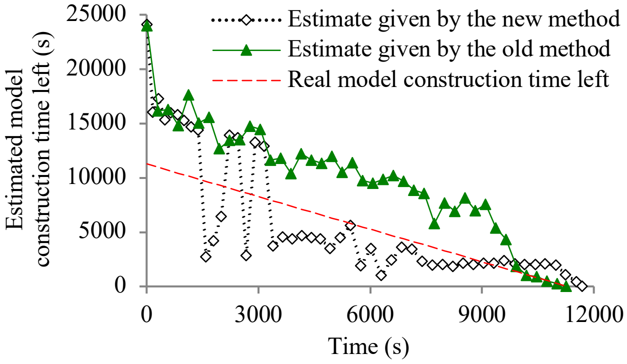

FIGURE 67. **Estimated model construction time left (using AdaGrad and applying an exponential decay method to the learning rate to construct GoogLeNet).**

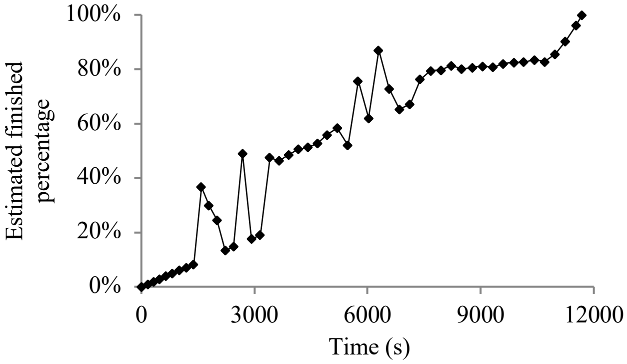

FIGURE 68. **Finished percentage estimated over time (using AdaGrad and applying an exponential decay method to the learning rate to construct GoogLeNet).**

1) TEST RESULTS FOR CONSTRUCTING THE GRU MODEL

Adopting the Adam optimization algorithm

In the test, we used the Adam optimization algorithm and applied an exponential decay method to the learning rate to construct the GRU model. Fig. 69-72 present the test results, which are akin to those presented in Fig. 22-25.

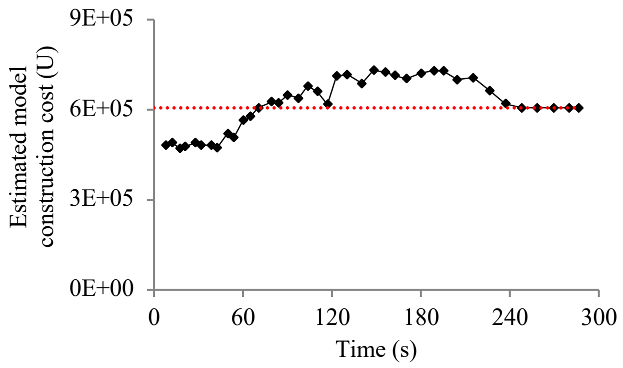

FIGURE 69. Model construction cost estimated over time (using Adam and applying an exponential decay method to the learning rate to construct the GRU model).

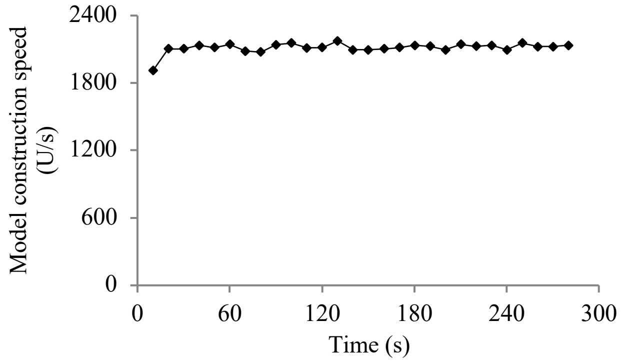

FIGURE 70. Model construction speed over time (using Adam and applying an exponential decay method to the learning rate to construct the GRU model).

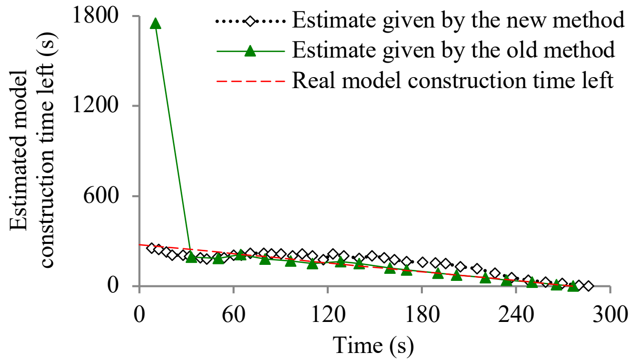

FIGURE 71. Estimated model construction time left (using Adam and applying an exponential decay method to the learning rate to construct the GRU model).

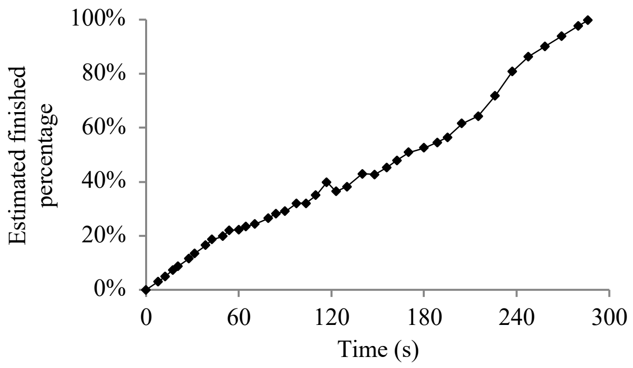

FIGURE 72. Finished percentage estimated over time (using Adam and applying an exponential decay method to the learning rate to construct the GRU model).

Adopting the SGD optimization algorithm

In the test, we used the SGD optimization algorithm and applied an exponential decay method to the learning rate to construct the GRU model. The test results are presented in Fig. 73-76.

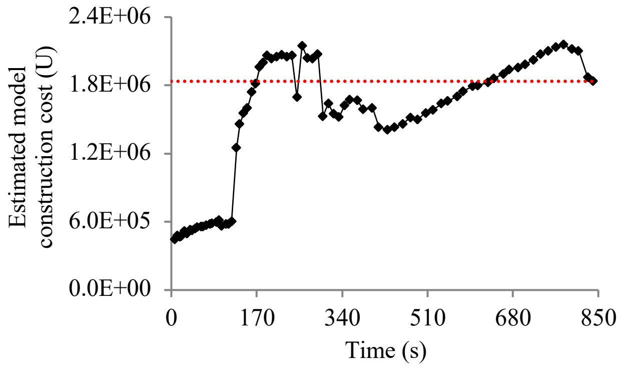

FIGURE 73. Model construction cost estimated over time (using SGD and applying an exponential decay method to the learning rate to construct the GRU model).

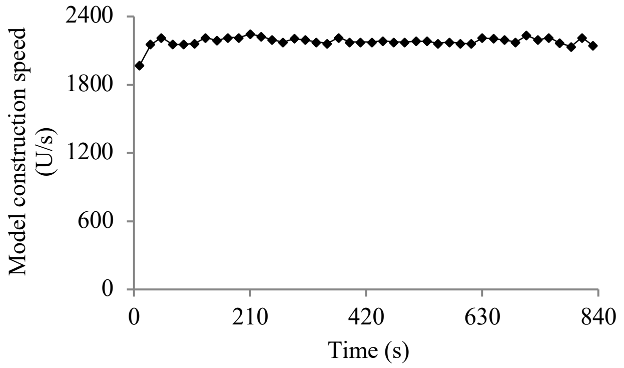

FIGURE 74. Model construction speed over time (using SGD and applying an exponential decay method to the learning rate to construct the GRU model).

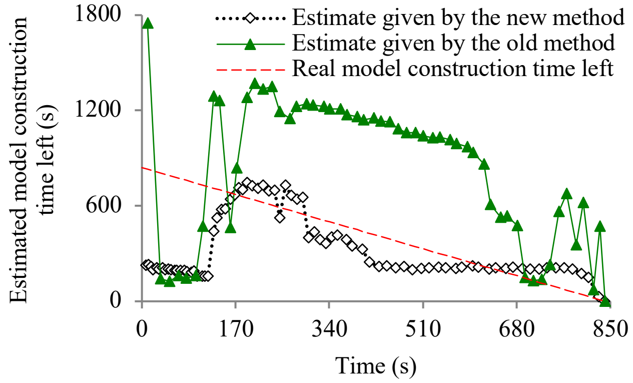

FIGURE 75. Estimated model construction time left (using SGD and applying an exponential decay method to the learning rate to construct the GRU model).

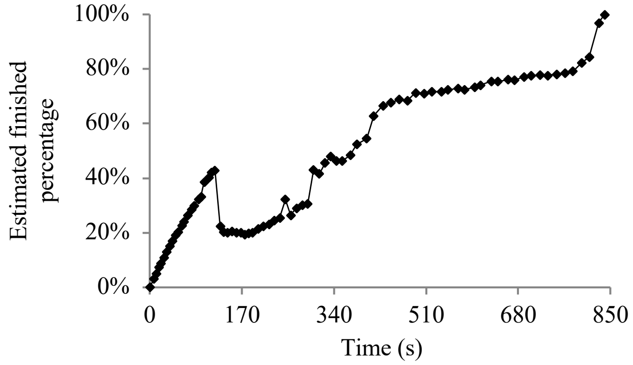

FIGURE 76. Finished percentage estimated over time (using SGD and applying an exponential decay method to the learning rate to construct the GRU model).

Adopting the AdaGrad optimization algorithm

In the test, we used the AdaGrad optimization algorithm and applied an exponential decay method to the learning rate to construct the GRU model. Fig. 77-80 present the test results, which are akin to those presented in Fig. 22-25.

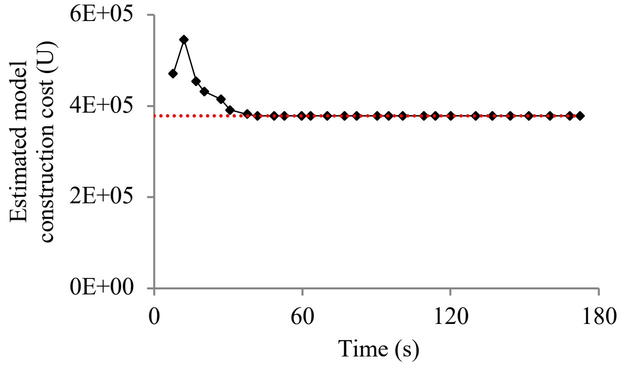

FIGURE 77. Model construction cost estimated over time (using AdaGrad and applying an exponential decay method to the learning rate to construct the GRU model).

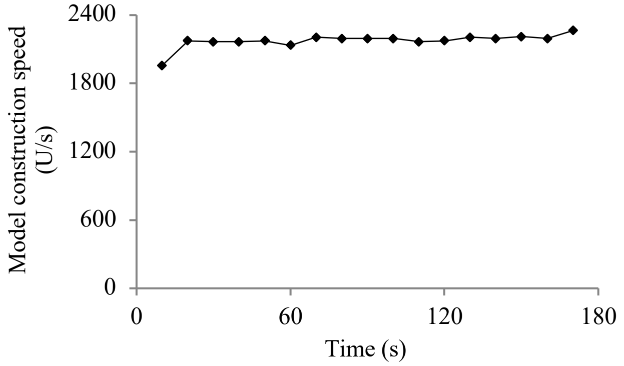

FIGURE 78. Model construction speed over time (using AdaGrad and applying an exponential decay method to the learning rate to construct the GRU model).

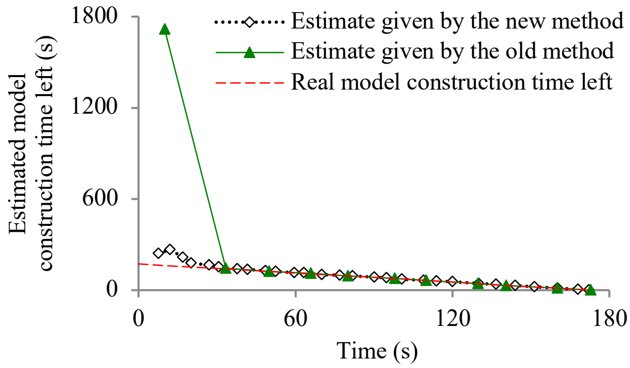

FIGURE 79. Estimated model construction time left (using AdaGrad and applying an exponential decay method to the learning rate to construct the GRU model).

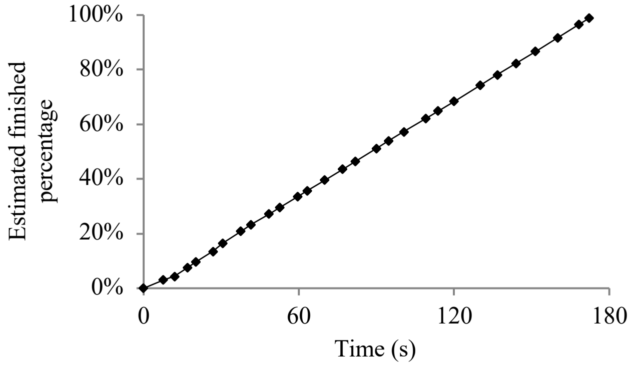

FIGURE 80. Finished percentage estimated over time (using AdaGrad and applying an exponential decay method to the learning rate to construct the GRU model).

C. OTHER TEST RESULTS FOR APPLYING A STEP DECAY METHOD TO THE LEARNING RATE

Recall that in the tests that applied a step decay method to the learning rate, we cut the learning rate from 10^{-3} to 10^{-4} at the start of the 64-th epoch, and subsequently to 10^{-5} at the start of the 115-th epoch. In every figure of this section, if applicable, we employ a dash-dotted vertical line to show the time at which a learning rate decay took place.

2) TEST RESULTS FOR CONSTRUCTING GOOGLENET

Adopting the RMSprop optimization algorithm

In the test, we used the RMSprop optimization algorithm and applied a step decay method to the learning rate to construct GoogLeNet. Early stopping happened on the second piece of the validation curve. Fig. 81-85 show the test results.

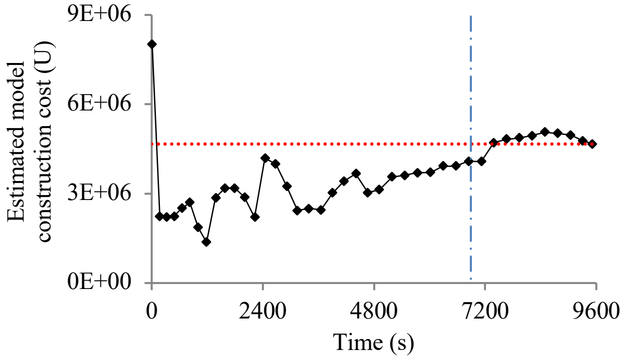

FIGURE 81. Model construction cost estimated over time (using RMSprop and applying a step decay method to the learning rate to construct GoogLeNet).

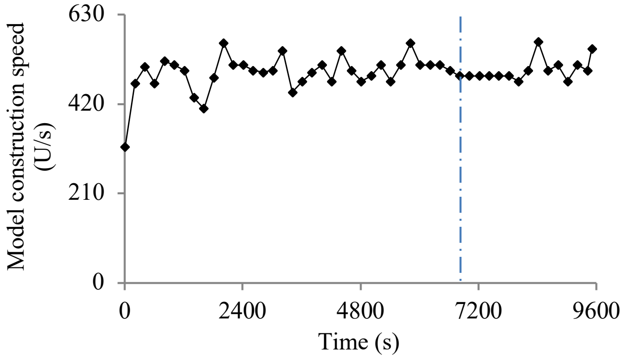

FIGURE 82. Model construction speed over time (using RMSprop and applying a step decay method to the learning rate to construct GoogLeNet).

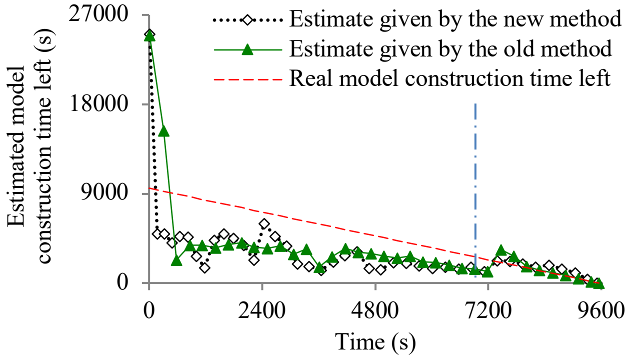

FIGURE 83. Estimated model construction time left (using RMSprop and applying a step decay method to the learning rate to construct GoogLeNet).

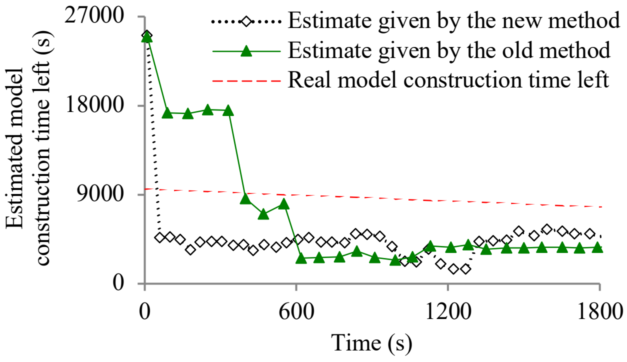

FIGURE 84. Estimate of the model construction time left at the early stage of model construction (using RMSprop and applying a step decay method to the learning rate to construct GoogLeNet).

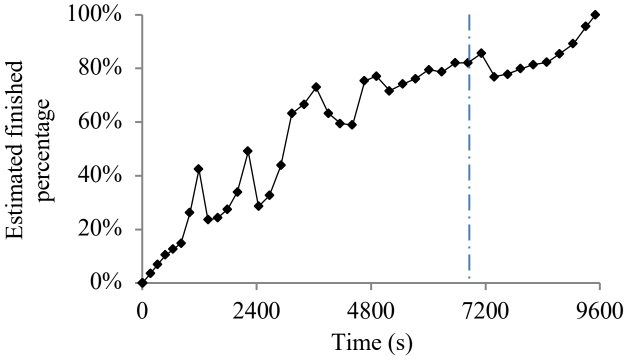

FIGURE 85. Finished percentage estimated over time (using RMSprop and applying a step decay method to the learning rate to construct GoogLeNet).

Adopting the SGD optimization algorithm

In the test, we used the SGD optimization algorithm and applied a step decay method to the learning rate to construct GoogLeNet. Early stopping happened on the second piece of the validation curve. Fig. 86-89 display the test results.

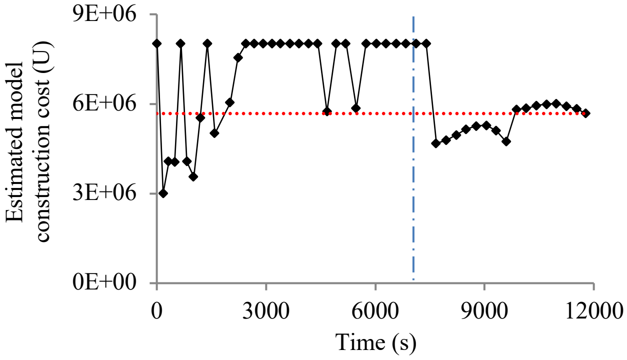

FIGURE 86. Model construction cost estimated over time (using SGD and applying a step decay method to the learning rate to construct GoogLeNet).

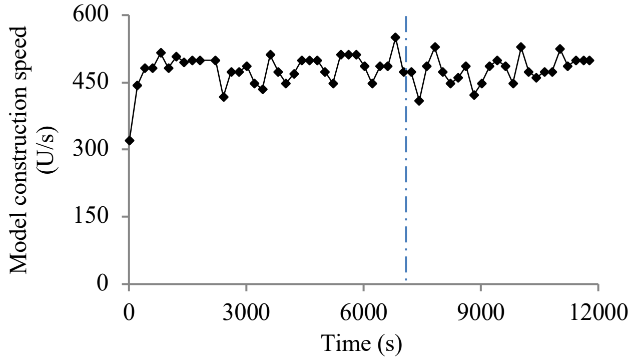

FIGURE 87. **Model construction speed over time (using SGD and applying a step decay method to the learning rate to construct GoogLeNet).**

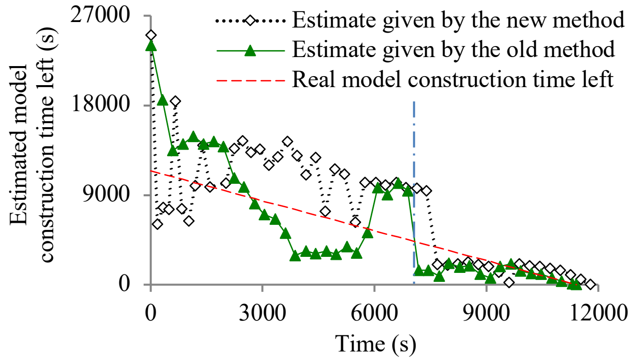

FIGURE 88. **Estimated model construction time left (using SGD and applying a step decay method to the learning rate to construct GoogLeNet).**

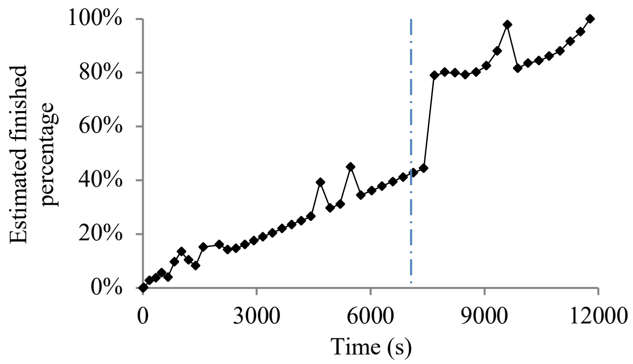

FIGURE 89. **Finished percentage estimated over time (using SGD and applying a step decay method to the learning rate to construct GoogLeNet).**

Adopting the AdaGrad optimization algorithm

In the test, we used the AdaGrad optimization algorithm and applied a step decay method to the learning rate to construct GoogLeNet. Early stopping happened on the third piece of the validation curve. Fig. 90-93 present the test results, which are akin to those presented in Fig. 86-89.

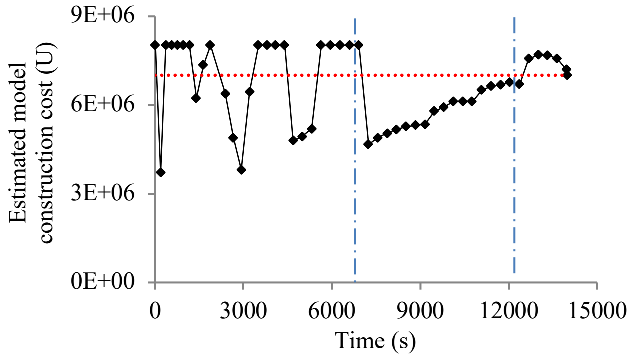

FIGURE 90. **Model construction cost estimated over time (using AdaGrad and applying a step decay method to the learning rate to construct GoogLeNet).**

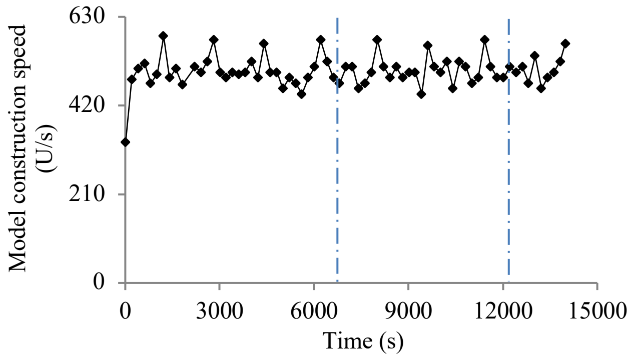

FIGURE 91. **Model construction speed over time (using AdaGrad and applying a step decay method to the learning rate to construct GoogLeNet).**

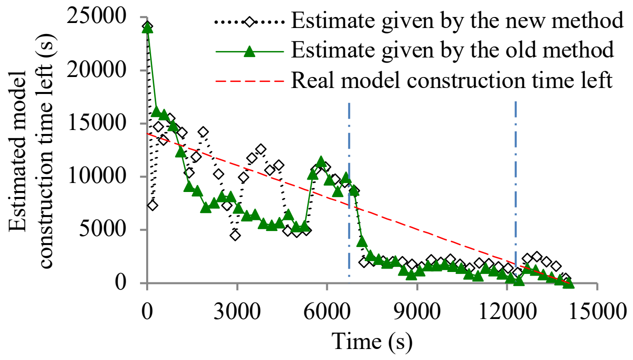

FIGURE 92. **Estimated model construction time left (using AdaGrad and applying a step decay method to the learning rate to construct GoogLeNet).**

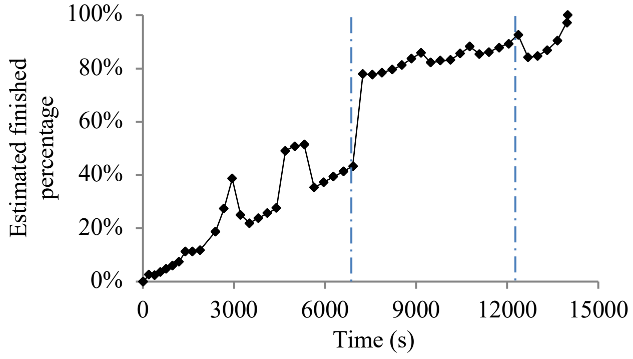

FIGURE 93. **Finished percentage estimated over time (using AdaGrad and applying a step decay method to the learning rate to construct GoogLeNet).**

3) TEST RESULTS FOR CONSTRUCTING THE GRU MODEL

Adopting the Adam optimization algorithm

In the test, we used the Adam optimization algorithm and applied a step decay method to the learning rate to construct the GRU model. Early stopping happened on the first piece of the validation curve. Fig. 94-97 present the test results and show that our progress indicator gave decently accurate estimates during most of the model construction process.

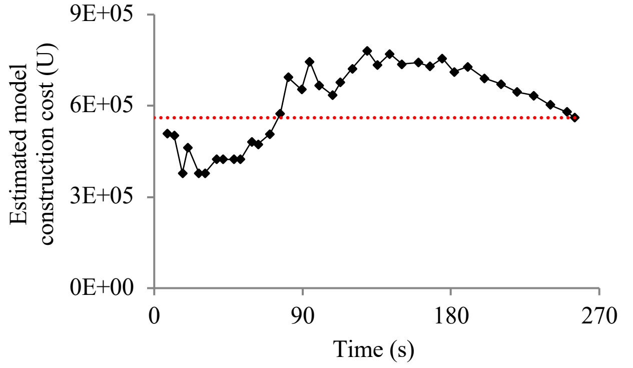

FIGURE 94. **Model construction cost estimated over time (using Adam and applying a step decay method to the learning rate to construct the GRU model).**

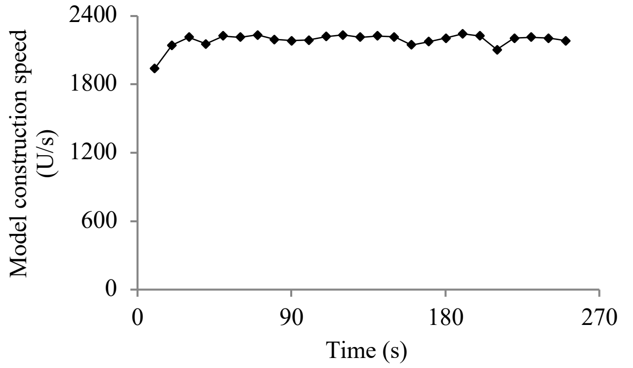

FIGURE 95. **Model construction speed over time (using Adam and applying a step decay method to the learning rate to construct the GRU model).**

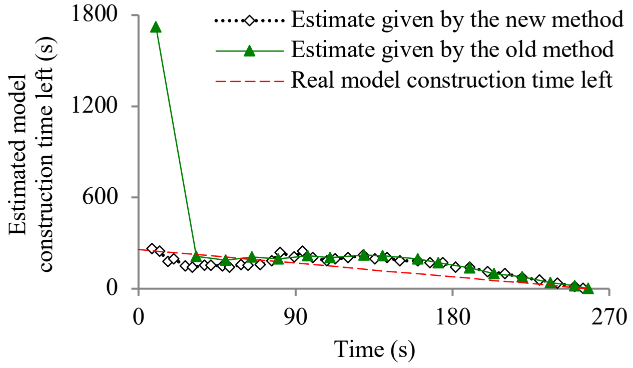

FIGURE 96. **Estimated model construction time left (using Adam and applying a step decay method to the learning rate to construct the GRU model).**

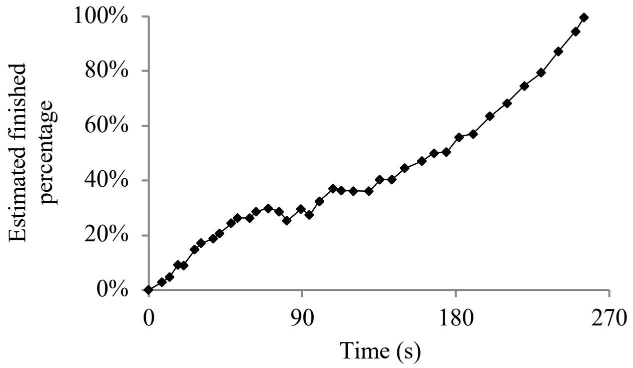

FIGURE 97. **Finished percentage estimated over time (using Adam and applying a step decay method to the learning rate to construct the GRU model).**

Adopting the RMSprop optimization algorithm

In the test, we used the SGD optimization algorithm and applied a step decay method to the learning rate to construct the GRU model. Early stopping happened on the first piece of the validation curve. Fig. 98-101 present the test results, which are akin to those presented in Fig. 94-97.

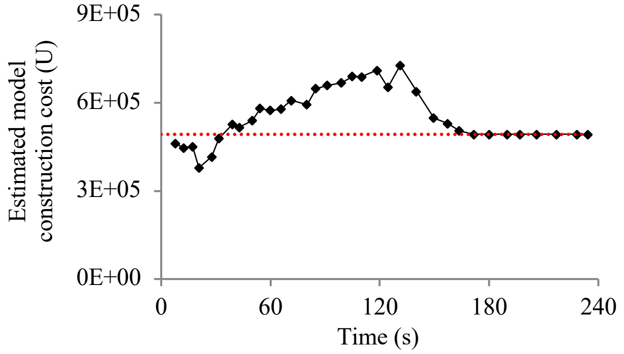

FIGURE 98. **Model construction cost estimated over time (using RMSprop and applying a step decay method to the learning rate to construct the GRU model).**

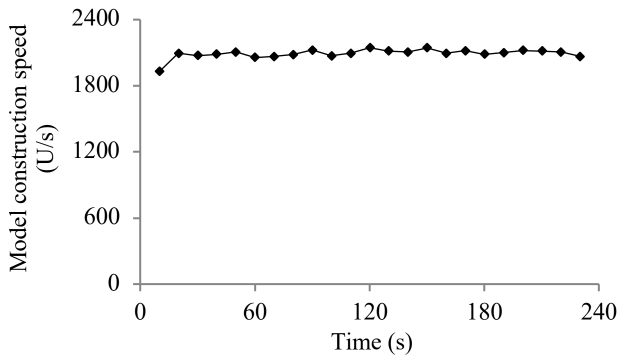

FIGURE 99. **Model construction speed over time (using RMSprop and applying a step decay method to the learning rate to construct the GRU model).**

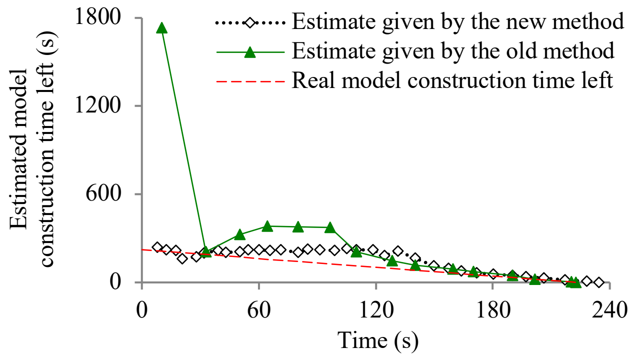

FIGURE 100. **Estimated model construction time left (using RMSprop and applying a step decay method to the learning rate to construct the GRU model).**

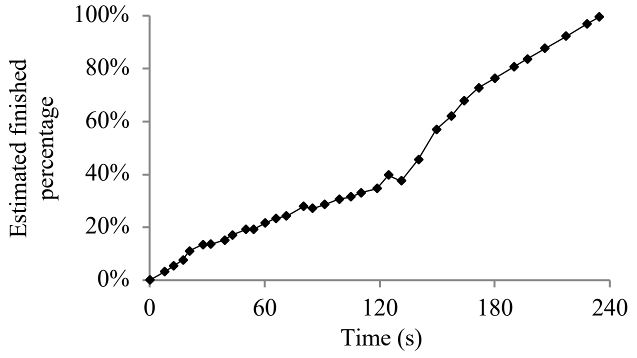

FIGURE 101. **Finished percentage estimated over time (using RMSprop and applying a step decay method to the learning rate to construct the GRU model).**

Adopting the SGD optimization algorithm

In the test, we used the SGD optimization algorithm and applied a step decay method to the learning rate to construct the GRU model. Early stopping happened on the second piece of the validation curve. The test results are presented in Fig. 102-105.

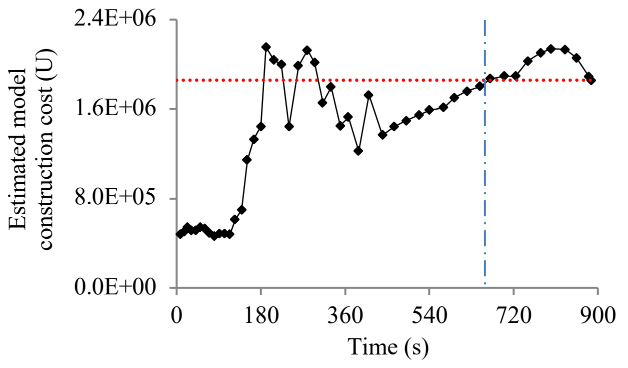

FIGURE 102. **Model construction cost estimated over time (using SGD and applying a step decay method to the learning rate to construct the GRU model).**

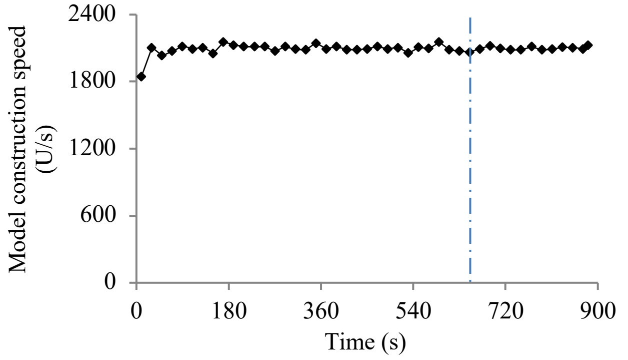

FIGURE 103. **Model construction speed over time (using SGD and applying a step decay method to the learning rate to construct the GRU model).**

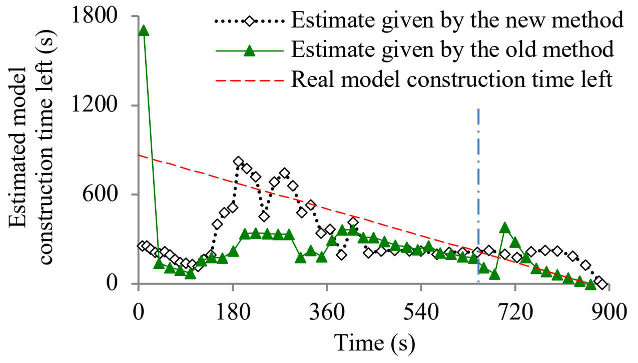

FIGURE 104. **Estimated model construction time left (using SGD and applying a step decay method to the learning rate to construct the GRU model).**

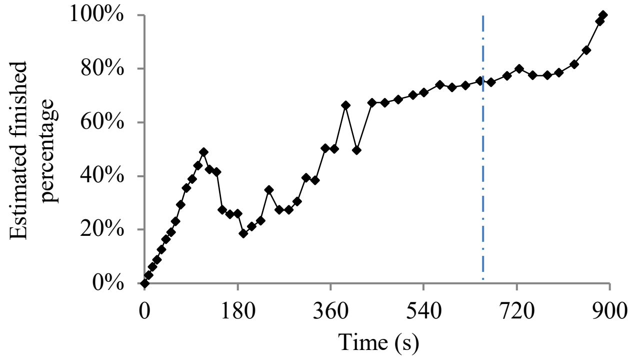

FIGURE 105. Finished percentage estimated over time (using SGD and applying a step decay method to the learning rate to construct the GRU model).

Adopting the AdaGrad optimization algorithm

In the test, we used the AdaGrad optimization algorithm and applied a step decay method to the learning rate to construct the GRU model. Early stopping happened on the first piece of the validation curve. Fig. 106-109 present the test results, which are akin to those presented in Fig. 94-97.

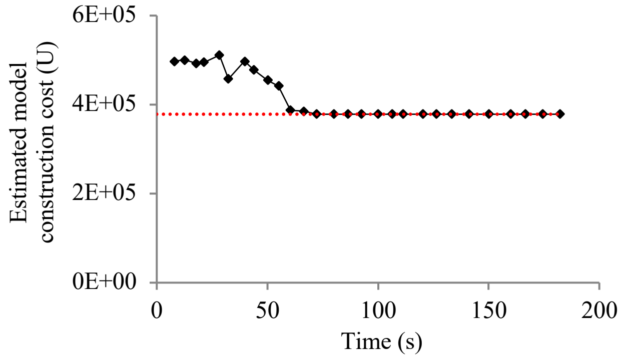

FIGURE 106. Model construction cost estimated over time (using AdaGrad and applying a step decay method to the learning rate to construct the GRU model).

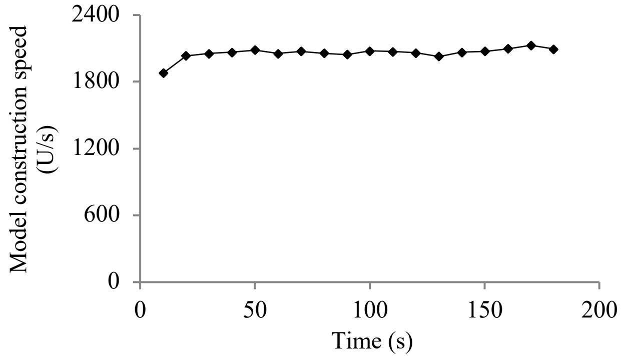

FIGURE 107. Model construction speed over time (using AdaGrad and applying a step decay method to the learning rate to construct the GRU model).

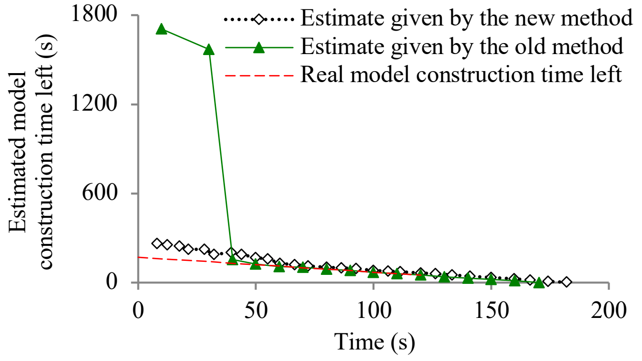

FIGURE 108. Estimated model construction time left (using AdaGrad and applying a step decay method to the learning rate to construct the GRU model).

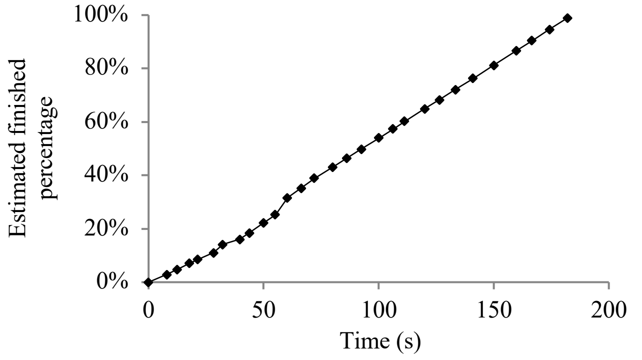

FIGURE 109. Finished percentage estimated over time (using AdaGrad and applying a step decay method to the learning rate to construct the GRU model).
